# Synthesis, crystal structures and properties of carbazole-based [6]helicenes fused with an azine ring

**DOI:** 10.3762/bjoc.17.2

**Published:** 2021-01-04

**Authors:** Daria I Tonkoglazova, Anna V Gulevskaya, Konstantin A Chistyakov, Olga I Askalepova

**Affiliations:** 1 Department of Chemistry, Southern Federal University, Zorge str., 7, Rostov-on-Don 344090, Russian Federation; 2 I. Ya. Postovsky Institute of Organic Synthesis, Ural Branch of the Russian Academy of Sciences, S. Kovalevskaya Str., 22, Yekaterinburg 620219, Russian Federation

**Keywords:** azine-fused helicenes, carbazole-based [6]helicenes, helical structures

## Abstract

Novel carbazole-based [6]helicenes fused with an azine ring (pyridine, pyrazine or quinoxaline) have been prepared through a five-step synthetic sequence in good overall yields. Commercially available 2,3-dihaloazines were used as starting materials. To discern the effect of merging an azine moiety within a helical skeleton, the X-ray structures, UV–vis absorption and fluorescence spectra of the helicenes were investigated and compared to that of the parent carbazole-based [6]helicene (7*H*-phenanthro[3,4-*c*]carbazole).

## Introduction

[*n*]Helicenes are polycyclic aromatic molecules with nonplanar screw-shaped helical skeletons formed by *n*-*ortho*-fused benzene or other aromatic rings. Their helical structure is a consequence of the steric repulsion of the terminal aromatic nuclei. The steric strain releases by adopting either the *P-* or *M-*helix configuration. The helically extended π-conjugated system, axial chirality and associated with these structural peculiarities unique optical and electronic properties of helicenes have attracted scientific interest for decades [1–11]. Compared to other planar π-conjugated systems, helicenes are more thermally stable and soluble in common organic solvents [9]. This fact together with the exceptionally high values of specific optical rotation and strong circular dichroism have led to promising applications of helicenes. The latter have been studied with respect to conductivity [12–15], nonlinear optics [16–17], circularly polarized luminescence [18–24], organocatalysis [25–29], conformational analysis [30], chirality sensing [31], chemical sensors [32], DNA-intercalators [33–34] etc.

Besides typical carbohelicenes, heterohelicenes, incorporating one or more heteroaromatic units in the skeleton, have also gained increasing attention [1–11]. The presence of heteroatoms (S, N, O, P) in the fused polycyclic π-systems additionally contributes to altering electronic structure and helps to fine tune optoelectronic properties [1–11,20,35–37]. The last decades highlighted heterohelicenes, incorporating one or two carbazole fragments, as a very attractive class of molecules [38–51]. This is not surprising, taking into account the excellent thermal stability, the strong electron-donating nature, a good hole-transporting ability of the carbazole unit and, as a consequence, numerous applications of the carbazole-based electroactive materials [52–54]. Carbazole-based [6]helicenes [42] and [7]helicenes [50] showed deep blue electroluminescence and have been investigated in OLED devices. Some carbazole-based [5]- and [6]helicenes have been used as visible light photoinitiators for cationic and radical polymerization [41]. [7]Helicenes of this group demonstrated a relatively high electron affinity and could be good candidates for electron-injection hole-blocking layers [39]. Donor–acceptor hybride [6]helicenes, consisting of carbazole and phenanthridine cores, are interesting as hole-transporting compounds [47]. At last, carbazole-based heterohelicenes were found in nature, for example, purpurone [55] isolated from the marine sponge *Iotrochota sp*. and having an inhibitory effect on the ATP-citrate lyase, and the marine alkaloid ningalin D produced by *Didemnum sp.*, Dictyodendrins [56] isolated from *Dictyodendrilla sp.* and displaying inhibitory activities towards telomerases.

**Scheme 1:**
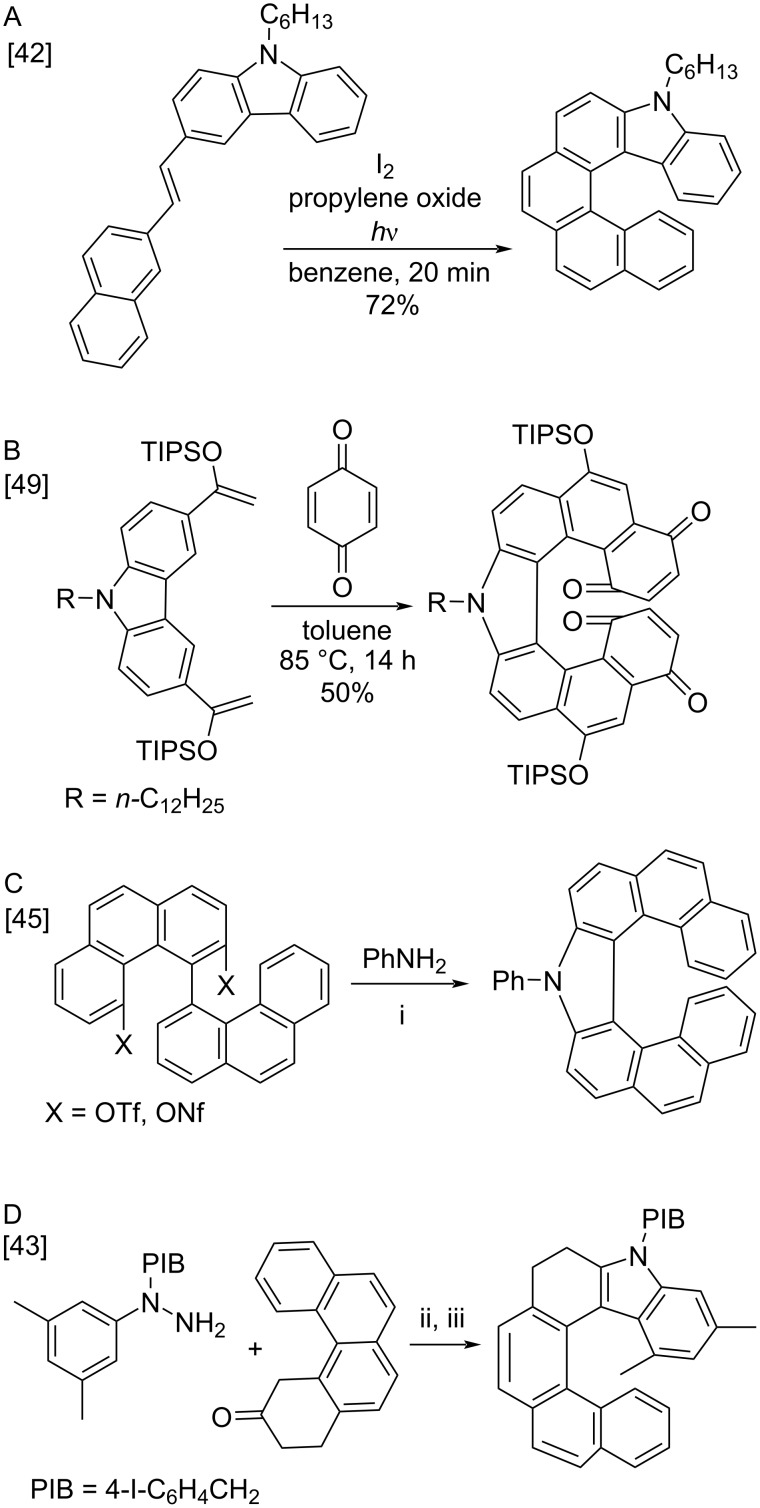
Overview of the synthetic methods for the carbazole-based heterohelicenes. i) Pd_2_dba_3_, xantphos, K_3_PO_4_, xylene, 100 °C, 123 h, 64–88% ii) SPINOL-derived phosphoric acid, Amberlite CG50, CH_2_Cl_2_, −7 °C, 72 h, 74%; iii) chloranil, diphenyl phosphate, CHCl_3_, 50 °C, 5 h, 76%.

The classical synthetic approach for carbohelicenes is the oxidative photocyclization of stilbene derivatives [1–11]. The latter are generally available via the Wittig, Heck-type or McMurry couplings. It is also a useful way to synthesize heterohelicenes, in particular, carbazole-based helicenes [39–42,44,47–48,50–51] (Scheme 1A). However, photocyclization of the stilbene substrates, having two non-equivalent *ortho* positions, leads to the formation of isomeric polynuclear molecules, which are often difficult to separate. Another drawback of this method is the difficulty of scaling, since the reaction requires strong dilution to prevent the [2π + 2π] dimerization of the starting stilbene. Among other approaches to the carbazole-based helicenes are the Diels–Alder reaction of silyl enol ethers of 3,6-diacetylcarbazole with *p*-benzoquinone (Scheme 1B) [49], the double Buchwald–Hartwig amination of 4,4'-biphenanthrene derivatives (Scheme 1C) [45] and a enantioselective Fischer indolization–oxidation protocol (Scheme 1D) [43]. Each method is not without drawbacks such as hardly available starting materials, rather expensive catalysts, harsh reaction conditions or low product yields.

**Scheme 2:**
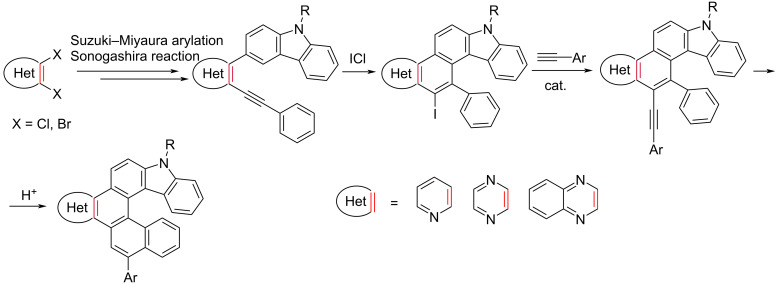
Synthetic strategy for the carbazole-based [6]helicenes fused with an azine ring.

Transition-metal-catalyzed, electrophile-induced and oxidative radical cyclizations of *ortho*-alkynylated biaryls are widely used for the synthesis of polynuclear aromatics [57–69]. Recently, we have described a versatile method for the preparation of aza[4]helicenes [70], diaza[4]helicenes [70–71] and azine-fused [5]helicenes [72] through a five-step synthetic sequence, using commercially available 2,3-dihaloazines as starting materials. Based on this approach, we synthesized carbazole-based [6]helicenes fused with an azine ring (quinoxaline, pyrazine or pyridine ones). Scheme 2 represents the current work. We envisaged that combining the carbazole unit with the readily available azine building block would result in the formation of donor–acceptor hybrid helicenes with interesting electronic properties.

## Results and Discussion

### Synthesis

In accordance with the above strategy, we first synthesized *ortho*-halogen alkynylazines **1a**,**b** via the Sonogashira reaction of commercially available 2,3-dichloroquinoxaline and 2,3-dichloropyrazine with phenylacetylene using a known procedure [70]. Coupling of compounds **1a**,**b** with 9-ethyl-3-(4,4,5,5-tetramethyl-1,3,2-dioxaborolan-2-yl)-9*H*-carbazole in the Pd(PPh_3_)_4_/K_2_CO_3_/1,4-dioxane/H_2_O catalytic system for 24 h at 100 °C (method C) afforded the desired 3-alkynyl-2-carbazolylazines **2a**,**b** in 82–96% yields (Table 1). Other catalytic systems Pd(PPh_3_)_4_/K_3_PO_4_/THF (method A) and Pd(PPh_3_)_4_/K_3_PO_4_/1,4-dioxane (method B) were less effective.

**Table 1:** Suzuki coupling of compounds **1a**,**b** with 9-ethyl-3-(4,4,5,5-tetramethyl-1,3,2-dioxaborolan-2-yl)-9*H*-carbazole.

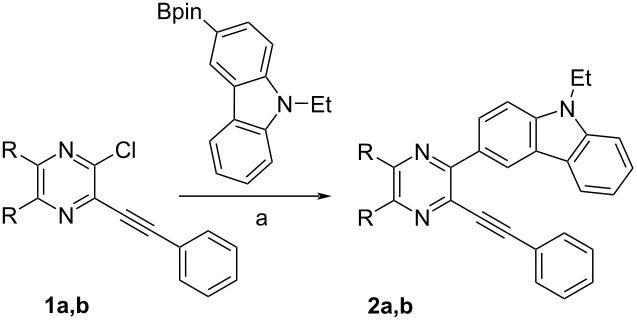			

Entry	R, R	Product	Yield, % (method)

1	-(CH=CH)_2_-	**2a**	36 (A) 44 (B) 96 (C)
2	H, H	**2b**	82 (C)

^a^Method A: Pd(PPh_3_)_4_, K_3_PO_4_, THF, reflux, 24 h, argon; method B: Pd(PPh_3_)_4_, K_3_PO_4_, 1,4-dioxane, 100 °C, 24 h, argon; method C: Pd(PPh_3_)_4_, K_2_CO_3_, 1,4-dioxane, H_2_O, 100 °C, 24 h, argon.

**Scheme 3:**
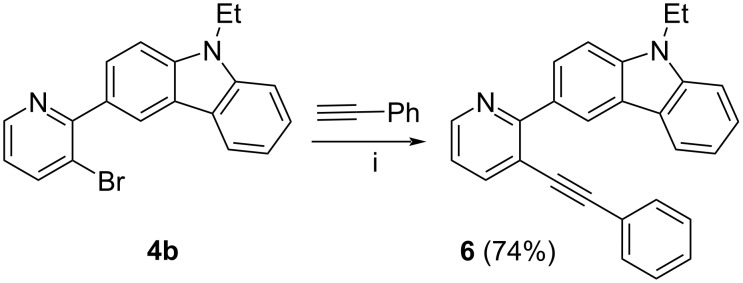
Sonogashira coupling of compound **4b** with phenylacetylene. i) Pd(PPh_3_)_2_Cl_2_, CuI, iPr_2_NH, DMSO, 80 °C, 24 h, argon.

3-Alkynyl-2-carbazolylazines can also be prepared using an alternative synthetic sequence, i.e., the Suzuki–Miyaura arylation–Sonogashira reaction. It should be noted that in the case of 2,3-dibromopyridine it was the only way for us to synthesize the target [4]helicenes [70]. Unfortunately, the coupling of 2,3-dichloroquinoxaline (**3a**) with 9-ethyl-3-(4,4,5,5-tetramethyl-1,3,2-dioxaborolan-2-yl)-9*H*-carbazole in the 5% Pd-C/PPh_3_/K_2_CO_3_/toluene/H_2_O catalytic system at 100 °C for 24 h (method A) gave the corresponding carbazolyl derivative **4a** in 15% yield only (Table 2). The Pd(PPh_3_)_4_/K_2_CO_3_/1,4-dioxane/H_2_O catalytic system (method B) was more effective producing **4a** in 63% yield. Thus, it was not the way for the synthesis of compounds **2a**,**b**. Arylation of 2,3-dibromopyridine using the Pd(PPh_3_)_4_/K_2_CO_3_/1,4-dioxane/H_2_O catalytic system gave a mixture of the corresponding mono- and dicarbazolyl derivatives **4b** (80%) and **5b** (8%). The products were easily separated by column chromatography. 3-Bromo-2-carbazolylpyridine **4b** was then introduced into the Sonogashira reaction with phenylacetylene giving rise to 3-alkynyl-2-carbazolylpyridine **6** in 74% yield (Scheme 3).

**Table 2:** Suzuki coupling of compounds **3** with 9-ethyl-3-(4,4,5,5-tetramethyl-1,3,2-dioxaborolan-2-yl)-9*H*-carbazole.

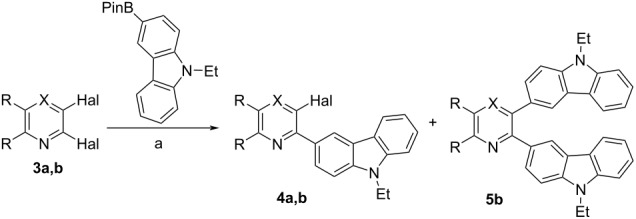					

Entry	X	R, R	Hal	Product	Yield, % (method)

1	N	-(CH=CH)_2_-	Cl	**4a**	15 (A) 63 (B)
2	CH	H, H	Br	**4b** **5b**	80 (B) 8 (B)

^a^Method A: 5% Pd/C, PPh_3_, K_2_CO_3_, H_2_O, toluene, 100 °C, 24 h, argon; method B: Pd(PPh_3_)_4_, K_2_CO_3_, 1,4-dioxane, H_2_O, 100 °C, 24 h, argon.

Electrophilic cyclizations of 3-alkynyl-2-carbazolylazines **2a**,**b** and **6** into azine-fused carbazoles **7a–c** were carried out with ICl in dry acetonitrile at room temperature in the dark (Table 3). In the cases of compounds **2a**,**b**, the reaction was found to be very sensitive to the amount of ICl used. In the case of **2a**, the use of a 1.5-fold excess of ICl led to a hardly separable mixture of compound **7a** and diiodo derivative **8a** (Table 3, entry 1, for the NMR spectrum of the mixture see Supporting Information File 1, Figure S13). Iodine chloride, taken in an equimolar amount, made it possible to obtain the required product **7a** in 65% yield and to remove minor impurities of diiodo derivative **8a** by chromatography (Table 3, entry 2). For the selective synthesis of **7b**, iodine chloride was taken in a small deficit (Table 3, entries 3 and 4). Compound **8b** was synthesized in 97% yield by the treatment with a 3-fold excess of ICl on monoiodide **7b**. The ICl-induced cyclization of the pyridine-based starting compound **6** proceeded smoothly giving rise to product **7c** in 62% (Table 3, entry 5). It should be noted that compounds **7a**, **7b** and **7c** are derivatives of the previously unknown heterocyclic systems 1*H*-carbazolo[3,4-*a*]phenazine, 7*H*-quinoxalino[5,6-*c*]carbazole and 7*H*-quinolino[8,7-*c*]carbazole, respectively (SciFinder data).

**Table 3:** ICl-induced cyclyzation of compounds **2** and **6**.

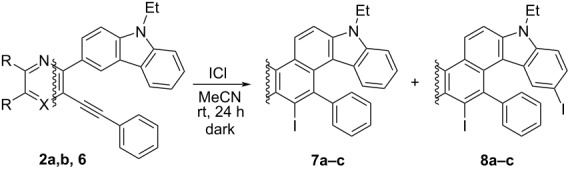					

Entry	X	R, R	ICl, equiv	Product	Yield, %

1	N	-(CH=CH)_2_-	1.5	**7a** + **8a**	92 (total) 7.7:1 ratio
2	N	-(CH=CH)_2_-	1	**7a**	65
3	N	H, H	1	**7b** + **8b**	69 (total) 10:1 ratio
4	N	H, H	0.75	**7b**	75
5	CH	H, H	1	**7c**	62

The fourth step of the synthesis of the target helicenes, namely the Sonogashira coupling of iodides **7a** and **7c** with *p*-tolylacetylene, was carried out in the Pd(PPh_3_)_2_Cl_2_/CuI/Et_3_N/THF catalytic system that proved itself well in the azine-fused [5]helicenes synthesis [72] (Table 4, entries 1 and 3). The corresponding compounds **9a** and **9c** were obtained in 84 and 88% yields, respectively. In the case of **7b**, the reaction was more selective without THF solvent producing alkyne **9b** in 82% yield (Table 4, entry 2). The structure of **9c** was unambiguously proved by X-ray structural analysis (see Supporting Information File 1, Figure S34).

**Table 4:** Sonogashira coupling of compounds **7** with *p*-tolylacetylene.

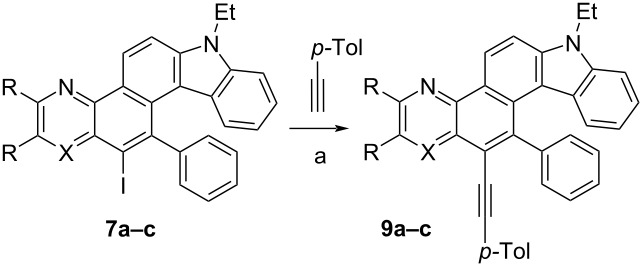					

Entry	X	R, R	Solvent	Product	Yield, %

1	N	-(CH=CH)_2_-	THF	**9a**	84
2	N	H, H	–	**9b**	82
3	CH	H, H	THF	**9c**	88

^a^Pd(PPh_3_)_2_Cl_2_, CuI, Et_3_N, solvent, 85 °C, 24 h, argon.

Earlier, at the final step of a similar synthesis of the azine-fused [5]helicenes, we used trifluoroacetic acid as a cyclizing agent [72]. Unfortunately, only in the case of compound **9c**, heating in trifluoroacetic acid led to isomerization into the required carbazole-based [6]helicene **10c** (Table 5, entry 3). Under these conditions, alkynes **9a** and **9b** produced an unseparable mixture of some products. To our delight, treating **9a** and **9b** with triflic acid in СH_2_Cl_2_ solution at room temperature allowed us to obtain helicenes **10a** and **10b** in high yields (Table 5, entries 1 and 2). Apparently, the presence of the aza group adjacent to the triple bond in compounds **9a** and **9b** causes the observed difference in the reactivity of compounds **9**. Its protonation makes the formation of a key cyclization intermediate **11** difficult.

**Table 5:** Acid-induced isomerization of compounds **9** into carbazole-based [6]helicenes **10**.

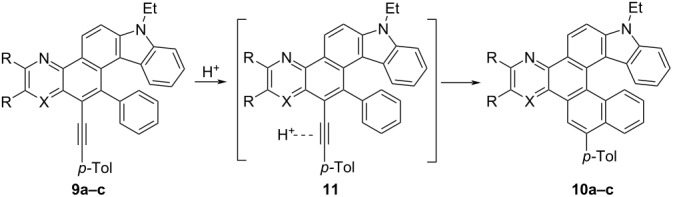					

Entry	X	R, R	Conditions	Product	Yield,%

1	N	-(CH=CH)_2_-	CF_3_SO_3_H, CH_2_Cl_2_, rt, 24 h, dark	**10a**	92
2	N	H, H	CF_3_SO_3_H, CH_2_Cl_2_, rt, 24 h, dark	**10b**	82
3	CH	H, H	CF_3_CO_2_H, 85 °C, 24 h	**10c**	94

It is known that the [*n*]helicenes with at least one five-membered heteroaromatic ring need *n* ≥ 6 to become intrinsically chiral [9–10]. High-performance liquid chromatography on chiral stationary phases confirmed the presence of configurationally stable (*P*)- and (*M*)*-*enantiomers at room temperature in the samples of synthesized helicenes **10a**–**c**. In all cases, separation of enantiomers of **10** was achieved using Kromasil 5-Cellucoat column (4.6 mm × 250 mm), acetonitrile as a mobile phase and UV-detection (see Supporting Information File 1, Figures S38–S40).

### X-ray molecular structures

**Figure 1:**
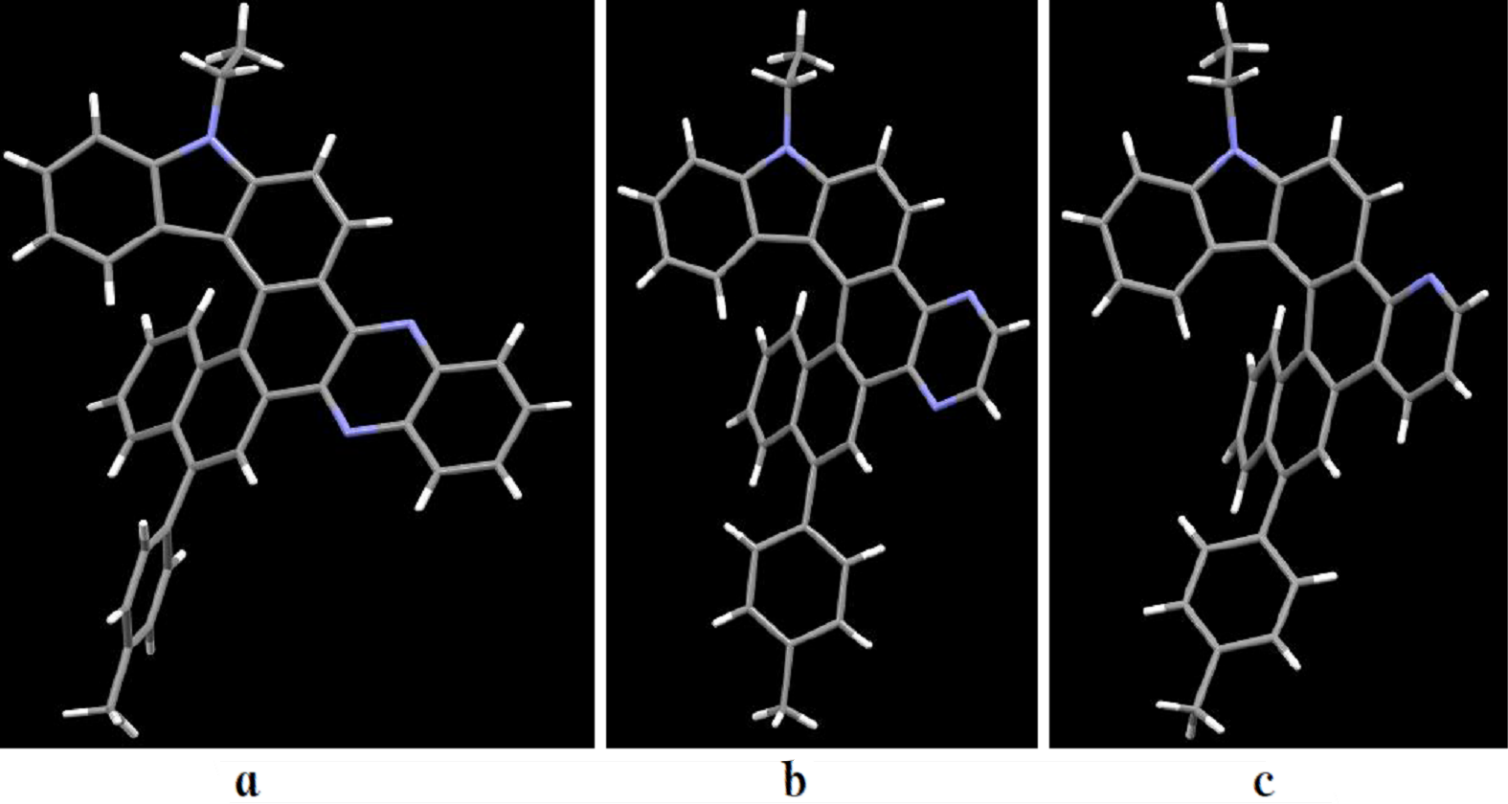
Molecular structure of carbazole-based [6]helicenes **10a** (a), **10b** (b) and **10c** (c) (X-ray data).

The structures of the title compounds **10a–c** were further explored by a single-crystal X-ray diffraction analysis (Figure 1, see also Supporting Information File 1, Figures S35–S37 and Table S1) and compared with that of the carbazole-based [6]helicene [42] (7-hexyl-7*H*-phenanthro[3,4-*c*]carbazole, **12**) to see the effect of the azine ring annelation (Table 6).

**Table 6:** Comparison of X-ray data of the carbazole-based [6]helicenes (atomic numbering does not correspond to IUPAC nomenclature).

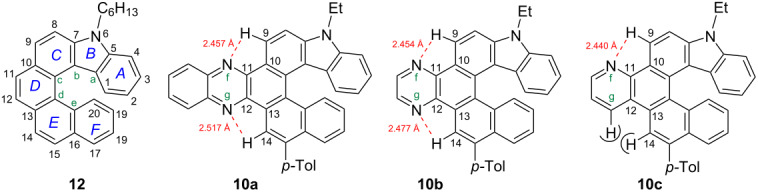							

Helicene	Space group	Bond length, Å		Inner helix angle, ^o^	Torsion angle^a^, ^o^		Interplanar angle^b^, ^o^
	
		Inner helix	Ring D				

		1–a a–b b–c c–d d–e e–20	10–11 11–12 12–13 13–d d–c c–10	1–a–b a–b–c b–c–d c–d–e d–e–20	1–a–b–c a–b–c–d b–c–d–e c–d–e–20	9–10–11–f g–12–13–14	A/F

**12** [42]	*P*2_1_/*c*	1.401 1.434 1.423 1.448 1.459 1.400 (8.565 in total)	1.391 1.343 1.434 1.407 1.448 1.435 (8.458 in total)	135.4 135.3 126.2 124.6 122.3 (643.8 in total)	0.8 17.8 32.2 16.9 (67.7 in total)	–	56.7

**10a**	*P*2_1_/*c*	1.403 1.457 1.423 1.470 1.441 1.415 (8.609 in total)	1.457 1.443 1.462 1.396 1.470 1.413 (8.641 in total)	135.7 135.4 125.0 123.3 121.4 (640.8 in total)	3.1 10.7 37.8 15.6 (67.2 in total)	7.6 2.4	50.5

**10b**^c^	*P*2_1_/*n*	1.408 1.456 1.430 1.464 1.442 1.417 (8.721 in total) (8.617 in total)*	1.449 1.412 1.451 1.400 1.464 1.418 (8.594 in total) (8.596 in total)*	135.8 135.6 125.3 123.7 121.0 (641.4 in total) (640.6 in total)*	0.8 11.3 32.3 21.6 (65.7 in total) (68.4 in total)*	5.3 2.4 3.2* 2.0*	50.6 53.7*

**10c**^a^	*Pca*2_1_	1.402 1.466 1.435 1.452 1.450 1.418 (8.623 in total) (8.603 in total)*	1.461 1.411 1.451 1.401 1.452 1.414 (8.590 in total) (8.605 in total)*	135.6 134.9 125.0 123.5 121.1 (640.1 in total) (640.8 in total)*	3.0 14.0 33.9 22.7 (73.6 in total) (72.3 in total)*	0.9 3.3 0.7* 3.4*	62.3 61.6*

^a^A dihedral angle between the four adjacent inner helix carbon atoms. ^b^An angle between the two terminal aromatic rings A and F of a helicene. ^c^There are two independent molecules in the unit cell. Data for the second molecule are marked with an asterisk *.

The values of the torsion and interplanar angles make it possible to describe and compare the structural specificity of the synthesized [6]helicenes. From the data given in Table 6, it can be seen that the interplanar angle between the terminal rings A and F of the pyridine-fused [6]helicene **10c** is the largest in the series (average value is 62°). The same value for [6]helicene **12** is equal to 56.7° [42]. The helicity of pyrazine-fused [6]helicene **10b** and quinoxaline-fused hybrid **10a** is intermediate (52.8° and 50.5°). Comparing the sum of the inner helix torsion angles of the helicenes **10a–c** and **12** reveals the same patterns. Evidently, the repulsive interaction of the H(14) and H(g) atoms of **10c** is the main reason for the observed extra twisting, whereas relative planarization of **10a** and **10b** is the result of attractive interactions of the aza groups N(f) and N(g) with the H(9) and H(14) atoms, respectively. The short intramolecular contacts H(9)···N(f) (2.457 Å for **10a**, 2.454 Å for **10b**) and H(14)···N(g) (2.517 Å for **10a**, 2.477 Å for **10b**) support this opinion. Apparently, interaction of this type is also realized in solution since the signal of the inner helix proton in the ^1^H NMR spectra of azine-fused [6]helicenes appeared in the low field at δ 9.3–9.7 ppm. A similar pattern was observed by us in the structures of the azine-fused [5]helicenes [72].

It is well-known that the inner helix and outer helix C–C bonds of helicenes differ in their length [9]. Deviations of some of them from the standard aromatic C–C bond of benzene (1.393 Å) are significant. The length of the inner helix C–C bonds of [6]helicene **12** varies from 1.400 to 1.459 Å. On the contrary, the outer helix bonds of **12** are noticeably shortened (1.330–1.368 Å). The same tendency is observed in cases of azine-fused [6]helicenes **10**. Their inner helix bonds are somewhat lengthened: 1.403–1.470 Å (for **10a**), 1.408–1.462 Å (for **10b**), 1.402–1.466 Å (for **10c**). The shortest outer helix bonds of helicenes **10** are 1.369 Å (**10a**), 1.369 Å (**10b**) and 1.364 Å (**10c**). The maximum deviation from the standard value was recorded for the C–C bonds of the central D ring of the [6]helicenes **10a**–**c**. In particular, the C(c)–C(d) length of quinoxaline-fused helicene **10a** (1.470 Å) practically does not differ in its length from the standard single C(sp^2^)–C(sp^2^) bond (ca. 1.48 Å).

**Figure 2:**
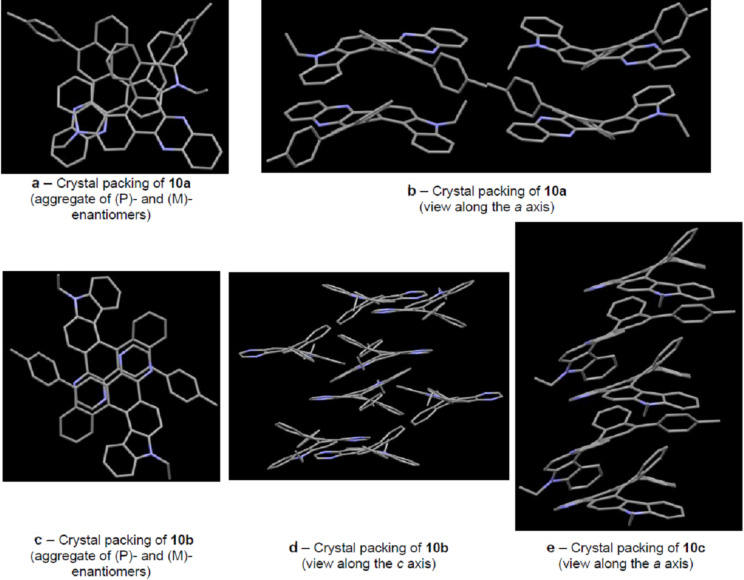
Crystal packing of carbazole-based [6]helicenes **10a** (a, b), **10b** (c,d) and **10c** (e). Hydrogen atoms are hidden.

The X-ray analysis of quinoxaline-fused [6]helicene **10a** revealed the presence of the face-to-face π–π interaction between the helicene aggregates. The racemic aggregation was composed by (*P*)*-* and (*M*)*-*enantiomers on the manner of embrace: π-deficient pyrazine ring of one enantiomer of **10a** is located over the π-excessive pyrrole ring of another enantiomer (Figure 2a and Figure 2b). An intermolecular distance between the centroids of these rings was found to be equal to 3.74 Å making the π overlapping possible. In the case of helicene **10b**, the enantiomeric molecules are aggregated into pairs differently: the pyrazine ring of one enantiomer is located over the E ring of another enantiomer and the distance between the layers is ca. 3.4 Å (Figure 2c and Figure 2d). The crystal packing of helicene **10c** (Figure 2e) is peculiar: the alternating enantiomers form a screw along the *a* axis.

### Optical properties

All helicenes **10** are well soluble in dichloromethane and chloroform. Solubility in other common solvents such as acetonitrile, DMSO, tetrahydrofuran, ethanol and hexane is markedly lower. Carbazole-based [6]helicene **12** was described as yellow solid (the longest λ_max_ 414 nm, CH_2_Cl_2_) [42]. All azine-fused analogs of **12** are yellow-orange. The annelation of the pyridine or pyrazine ring to the skeleton of **12** only slightly changed the wavelength of the absorption maximum (the longest λ_max_ 411 and 418 nm, respectively), whereas the absorption of quinoxaline-fused [6]helicene **10a** was red-shifted by 19 nm (Table 7). Compounds **10** exhibited almost a solvent independence of UV–vis absorption spectra (Supporting Information File 1, Figure S41). The optical band gaps (*E*_g_^opt^), estimated from the onset point of the absorption spectra, for [6]helicene **12** was equal to 2.92 eV [42]. The *E*_g_^opt^ values for its π-extended analogs were 2.45 eV (**10a**), 2.76 eV (**10b**) and 2.85 eV (**10c**), suggesting a higher HOMO and lower oxidation potential, which are typically desired characteristics when designing organic materials. Unfortunately, for all azine-fused [6]helicenes **10** only weak fluorescence in the solution under UV irradiation was observed (Table 7, see also Supporting Information File 1, Figures S42–S45). Helicenes **10b** and **10c** exhibited blue emission with emission peaks at 481 and 440 nm, respectively. Quinoxaline-fused helicene **10a** demonstrated a yellow emission with the highest in the series λ_em_ = 561 nm and Stokes shift 128 nm.

**Table 7:** Photophysical properties of carbazole-based [6]helicenes **10**.

Compd.	Absorption (CH_2_Cl_2_)^a^		*E*_g_^opt^, eV^b^	Absorption and emission^c^ (CH_3_CN)		
	
	λ_max_, nm	λ_onset_, nm		λ_abs_, nm^d^	λ_em_, nm	Stokes shift, nm

**12** [42]	282, 320, sh 347, 393, 414	425	2.92	414	426	12
**10a**	264, 303, sh 324, 357, 374, 433	507	2.45	433	561	128
**10b**	294, 323, 359, 397, 418	449	2.76	418	481	63
**10c**	265, 285, sh 306, 324, 371, sh 388, 411	435	2.85	410	440	30

^a^Absorption maxima measured in ≈10^−5^ M solution, abbreviation “sh” means shoulder. ^b^The optical gap was estimated from the onset point of the absorption spectra: *E*_g_^opt^ = 1240/λ_onset._. ^c^Excited at the longest absorption maxima. ^d^The only longest absorption maxima is shown.

## Conclusion

In summary, novel carbazole-based [6]helicenes fused with an azine ring (pyridine, pyrazine or quinoxaline) have been prepared from commercially available 2,3-dihaloazines via a five-step synthetic sequence. Two key steps of the method are electrophile-induced *6-endo-dig* cyclizations of *ortho*-alkynylated biaryls. The overall yields of helicenes in five stages of the synthesis exceed 30%.

The single-crystal X-ray diffraction analysis revealed the non-planar crystal structures of the synthesized helicenes responsible for reducing close-packing arrangement, though, in cases of pyrazine- and quinoxaline-fused helicenes, moderate π overlap in pairs of enantiomeric molecules was observed. Carbazole-based [6]helicene, fused with the pyridine ring, is more twisted than the parent carbazole-based [6]helicene (7*H*-phenanthro[3,4-*c*]carbazole). The interplanar angle between the two terminal benzene rings of the latter is equal to 56.7°, whereas the same value for the pyridine-fused analog is 62°. The pyrazine-fused [6]helicene demonstrates intermediate helicity (52.8°). In the case of the quinoxaline-fused analog the distortion angle of 50.5° is the smallest in the series.

The photophysical properties of the synthesized [6]helicenes were compared to the parent carbazole-based [6]helicene. A spectrophotometric analysis of the quinoxaline-fused helicene displayed a moderate absorption red-shift (19 nm) and reduced optical band gaps (by ≈0.5 eV). In cases of pyrazine and pyridine-fused analogs, differences are not so noticeable.

## Experimental

The synthetic procedures, HPLC, X-ray studies and spectra (^1^H and ^13^C NMR) of all new compounds can be found in Supporting Information File 1.

## Supporting Information

File 1 Experimental procedures and analytical data, copies of ^1^H and ^13^C NMR spectra of all new compounds, X-ray data for **9c** and **10a–c**, HPLC spectra of helicenes **10a–c**, UV–vis and fluorescence spectra of **10a–c**.
