# Supplementary material for: Synthesis, crystal structures and properties of carbazole-based [6]helicenes fused with an azine ring
Source: Beilstein J Org Chem. 2021 Jan 4;17:11–21. doi: 10.3762/bjoc.17.2 (PMC7801798; doi:10.3762/bjoc.17.2)
Supplement: File 1 — Experimental procedures and analytical data, copies of 1H and 13C NMR spectra of all new compounds, X-ray data for 9c and 10a–c, HPLC spectra of helicenes 10a–c, UV–vis and fluorescence spectra of 10a–c. [file Beilstein_J_Org_Chem-17-11-s001.pdf]

BEILSTEIN JOURNAL OF ORGANIC CHEMISTRY

Supporting Information

for

Synthesis, crystal structures and properties of carbazole-based [6]helicenes fused with an azine ring

Daria I. Tonkoglazova, Anna V. Gulevskaya, Konstantin A. Chistyakov and Olga I. Askalepova

Beilstein J. Org. Chem. **2021,** *17,* 11–21. doi:10.3762/bjoc.17.2

Experimental procedures and analytical data, copies of ^{1}H and ^{13}C NMR spectra of all new compounds, X-ray data for 9c and 10a–c, HPLC spectra of helicenes 10a–c, UV–vis and fluorescence spectra of 10a–c

License and Terms: This is a supporting information file under the terms of the Creative Commons Attribution License (https://creativecommons.org/licenses/by/4.0). Please note that the reuse, redistribution and reproduction in particular requires that the author(s) and source are credited and that individual graphics may be subject to special legal provisions.
The license is subject to the *Beilstein Journal of Organic Chemistry* terms and conditions: (https://www.beilstein-journals.org/bjoc/terms)

Table of contents

Experimental section………......S3

General information……...S3

Experimental procedures…………………………………………………………………………………………………...….S4

Copies of ^{1}H and ^{13}C NMR spectra (Figs. S1-S33)……………………………………………………………………..…….S12

X-ray data (Figs. S34-S37, Table S1)………………………………………………………………………………………………S45

HPLC separation (Figs. S38-S40) ………………………………………………………………………………… ……...S48

Absorption and fluorescence spectra (Figs. S41-S45)…………………………………………………………………………...S52

References……..S54

Experimental section

General information: ^{1}H and ^{13}C NMR spectra were recorded on a 250 MHz spectrometer (Bruker DPX-250). Chemical shifts were reported in ppm relative to Me_4Si. The UV-vis spectra were recorded on a Varian Cary 50 Probe spectrophotometer. Fluorescence spectra were recorded on a Varian Cary Eclipse Fluorescence Spectrophotometer. The HR-ESI mass-spectra were obtained on a BRUKER maXis spectrometer equipped with an electrospray ionization (ESI) source. Melting points were determined on a Stuart SMP30 instrument in glass capillaries and are uncorrected. Flash column chromatography was performed on silica gel (70–230 mesh, Aldrich). Reactions were monitored by thin layer chromatography (silica gel 60 F_{254}) and visualized using UV. Commercial alkynes, 9-ethyl-3-(4,4,5,5-tetramethyl-1,3,2-dioxaborolan-2-yl)-9*H*-carbazole, catalysts, ICl, 2,3-dihaloazines, diisopropylamine, triethylamine, PPh_3, TFA, triflic acid, anhydrous DMSO, THF were used as received.

9-Ethyl-3-(3-(phenylethynyl)quinoxalin-2-yl)-9*H*-carbazole (2a): A stirred mixture of 2-chloro-3-(phenylethynyl)quinoxaline **1a** [1] (132 mg, 0.5 mmol), 9-ethyl-3-(4,4,5,5-tetramethyl-1,3,2-dioxaborolan-2-yl)-9*H*-carbazole (161 mg, 0.5 mmol), $Pd(PPh_3)_4$ (58 mg, 0.05 mmol), K_2CO_3 (345 mg, 2.5 mmol), 1,4-dioxane (8 mL) and water (4 mL) was heated at 100 ^{o}C for 17 h under argon. After evaporatation of the reaction mixture the residue was diluted with water (50 mL) and extracted with CH_2Cl_2 (3 × 15 mL). The extract was dried over Na_2SO_4. Flash column chromatography was carried out on silica gel (3.5 × 50 cm) using CH_2Cl_2 as the eluent. The yellow fraction with R_f 0.45 gave compound **2a** (203 mg, 96%). Compound **2a** was obtained as lemon yellow needles with mp 158‒160 ^{o}C (EtOH). ^{1}H NMR (250 MHz, $CDCl_3$): δ = 1.47 (t, J = 7.1 Hz, 3 H), 4.42 (q, J = 7.1 Hz, 2 H), 7.21‒7.35 (m, 4 H), 7.43‒7.57 (m, 5 H), 7.70‒7.78 (m, 2 H), 8.12‒8.18 (m, 3 H), 8.29 (dd, J = 8.6, 1.4 Hz, 1 H), 9.03 (d, J = 1.0 Hz, 1 H) ppm. ^{13}C NMR (62.9 MHz, $CDCl_3$) δ 13.9, 37.8, 89.3, 94.9, 108.4, 108.8, 119.4, 120.8, 121.9, 122.4, 122.7, 123.2, 126.1, 127.9, 128.2, 128.5, 128.8, 129.2, 129.5, 129.8, 130.6, 132.3, 138.3, 140.6, 140.7, 140.9, 141.1, 155.5 ppm. HRMS (ESI): MH^+, found 424.1815. $C_{30}H_{22}N_3$ requires 424.1808. $M+Na^+$, found 446.1633. $C_{30}H_{21}N_3Na$ requires 446.1628.

Another catalytic systems, *e.g.* $Pd(PPh_3)_4/K_3PO_4$/THF (80 ^{o}C, 24 h, 36% yield) and $Pd(PPh_3)_4/K_3PO_4$/1,4-dioxane (100 ^{o}C, 24 h, 44% yield), were less effective.

9-Ethyl-3-(3-(phenylethynyl)pyrazin-2-yl)-9H-carbazole (2b): Synthesis of compound **2b** was carried out similarly to **2a** from 2-chloro-3-(phenylethynyl)pyrazine **1b** [1] (108 mg, 0.5 mmol). Flash column chromatography was performed on silica gel (2 × 20 cm) using ethylacetate - petrolium ether (1:3, v/v) as the eluent. From the yellow fraction with R_f 0.2–0.3 compound **2b** was isolated (153 mg, 82%). Compound **2b** was obtained as yellowish solid with mp 133–135 °C (CH_3CN). ^{1}H NMR (250 MHz, $CDCl_3$): δ = 1.52 (t, J = 7.2 Hz, 3 H), 4.47 (q, J = 7.2 Hz, 2 H), 7.26–7.40 (m, 4 H), 7.48–7.59 (m, 5 H), 8.14 (d, J = 7.7 Hz, 1 H), 8.28 (dd, J = 8.6, 1.7 Hz, 1 H), 8.54 (d, J = 2.3 Hz, 1 H), 8.64 (d, J = 2.3 Hz, 1 H), 9.02 (d, J = 1.6 Hz, 1 H) ppm. ^{13}C NMR (62.9 MHz, $CDCl_3$) δ 11.1, 35.0, 85.5, 91.5, 105.4, 106.0, 116.6, 117.8, 119.1, 119.2, 119.9, 120.4, 123.2, 124.5, 124.7, 125.6, 126.5, 129.2, 134.2, 137.7, 138.1, 138.6, 139.5, 153.3 ppm. HRMS (ESI): MH^+, found 374.1666. $C_{26}H_{20}N_3$ requires 374.1652. $M+Na^+$, found 396.1484. $C_{26}H_{19}N_3Na$ requires 396.1471.

3-(3-Chloroquinoxalin-2-yl)-9-ethyl-9*H*-carbazole (4a) was synthesized in a similar manner as descibed in [1]**.** A stirred mixture of 2,3-dichloroquinoxaline **3a** (100 mg, 0.5 mmol), 9-ethyl-3-(4,4,5,5-tetramethyl-1,3,2-dioxaborolan-2-yl)-9*H*-carbazole (193 mg, 0.6 mmol), 5% Pd/C (32 mg, 0.015 mmol), PPh_3 (16 mg, 0.06 mmol), 2M aqueous solution K_2CO_3 (2 mL) and toluene (1 mL) was heated at 100 °C for 24 h under argon. The reaction mixture was then extracted with $CHCl_3$ (3 × 20 mL). The extract was dried over Na_2SO_4 and purified by flash column chromatography on silica gel (3.5 × 45 cm) with $CHCl_3$ as the eluent. The first and second fractions were recovered starting materials: 43 mg (43%) of **3a** and 142 mg (73%) of the boronic acid. The fraction with R_f 0.6 gave compound **4a** (28 mg, 15%). Compound **4a** was obtained as lemon yellow crystals with mp 120–122 °C (EtOH). ^{1}H NMR (250 MHz, $CDCl_3$): δ = 1.51 (t, J = 7.2 Hz, 3 H), 4.47 (q, J = 7.2 Hz, 2 H), 7.31–7.34 (m, 1 H), 7.47–7.59 (m, 3 H), 7.78–7.85 (m, 2 H), 8.04–8.12 (m, 2 H), 8.19–8.22 (m, 2 H), 8.70 (d, J = 1.2 Hz, 1 H) ppm. ^{13}C NMR (62.9 MHz, $CDCl_3$) δ 13.9, 37.8, 108.3, 108.8, 119.5, 120.8, 122.5, 122.9, 123.1, 126.2, 126.4, 127.3, 127.5, 128.1, 129.2, 130.4 (2C), 140.6, 140.7, 140.8, 141.3, 153.8 ppm. HRMS (ESI): MH^+, found 358.1107 (^{35}Cl); 360.1081 (^{37}Cl). $C_{22}H_{17}ClN_3$ requires 358.1106 (^{35}Cl); 360.1078 (^{37}Cl). $M+Na^+$, found 380.0924 (^{35}Cl); 382.0902 (^{37}Cl). $C_{22}H_{16}ClN_3Na$ requires 380.0925 (^{35}Cl); 382.0897 (^{37}Cl).

3-(3-Bromopyridin-2-yl)-9-ethyl-9*H*-carbazole (4b) and 3,3'-(pyridine-2,3-diyl)bis(9-ethyl-9*H*-carbazole) (5b). A stirred mixture of 2,3-dibromopyridine **3b** (119 mg, 0.5 mmol), 9-ethyl-3-(4,4,5,5-tetramethyl-1,3,2-dioxaborolan-2-yl)-9*H*-carbazole (161 mg, 0.5 mmol), $Pd(PPh_3)_4$ (58

mg, 0.05 mmol), K_2CO_3 (345 mg, 2.5 mmol), 1,4-dioxane (8 mL) and water (4 mL) was heated at 100 °C for 17 h under argon. After evaporation of the reaction mixture the residue was diluted with water (100 mL) and extracted with CH_2Cl_2 (3 × 15 mL). The extract was dried over Na_2SO_4. Flash column chromatography on silica gel (3.5 × 55 cm) was then carried out using CH_2Cl_2 as the eluent. From the colorless fraction with R_f 0.3 compound **4b** was isolated (142 mg, 80%). The yellowish fraction with R_f 0.2 gave compound **5b** (20 mg, 8%).

3-(3-Bromopyridin-2-yl)-9-ethyl-9H-carbazole (**4b**). Yellowish oil. ^{1}H NMR (250 MHz, $CDCl_3$): δ = 1.52 (t, J = 7.1 Hz, 3 H), 4.46 (q, J = 7.1 Hz, 2 H), 7.17 (dd, J = 8.0, 4.6 Hz, 1 H), 7.28–7.34 (m, 1 H), 7.47–7.58 (m, 3 H), 7.91 (d, J = 8.5 Hz, 1 H), 8.07 (d, J = 8.0 Hz, 1 H), 8.20 (d, J = 7.7 Hz, 1 H), 8.55 (s, 1 H), 8.72 (d, J = 4.6 Hz, 1 H) ppm. ^{13}C NMR (62.9 MHz, $CDCl_3$): δ = 13.9, 37.7, 107.9, 108.7, 119.2, 120.1, 120.7, 121.9, 122.6, 122.7, 123.3, 125.9, 127.3, 130.4, 140.2, 140.5, 141.4, 148.1, 159.0 ppm. HRMS (ESI): MH^+ (^{81}Br), found 353.0476; MH^+ (^{79}Br), found 351.0497. $C_{19}H_{16}BrN_2$ requires 353.0472 (^{81}Br), 351.0491 (^{79}Br).

3,3'-(Pyridine-2,3-diyl)bis(9-ethyl-9H-carbazole) (**5b**). Yellowish oil. ^{1}H NMR (250 MHz, $CDCl_3$): δ = 1.38 (t, J = 7.2 Hz, 3 H), 1.41 (t, J = 7.2 Hz, 3 H), 4.29 (q, J = 7.2 Hz, 2 H), 4.33 (q, J = 7.2 Hz, 2 H), 7.12–7.27 (m, 5 H), 7.34–7.52 (m, 6 H), 7.90 (dd, J = 7.7, 1.6 Hz, 1 H), 7.97 (d, J = 7.7 Hz, 1 H), 8.05 (d, J = 7.7 Hz, 1 H), 8.12 (br s, 1 H), 8.38 (d, J = 1.3 Hz, 1 H), 8.77 (dd, J = 4.7, 1.6 Hz, 1 H) ppm. ^{13}C NMR (62.9 MHz, $CDCl_3$): δ = 13.7(8), 13.8(0), 37.5(8), 37.6(2), 107.6, 108.2, 108.4, 108.6, 118.8, 118.9, 120.5, 120.6, 121.2, 121.5, 122.3, 122.9, 123.0, 123.2, 123.4, 125.5, 125.8, 127.8, 128.2, 131.4, 131.5, 136.7, 139.1, 139.2, 139.6, 140.3, 147.9, 158.1 ppm. HRMS (ESI): MH^+, found 466.2287. $C_{33}H_{28}N_3$ requires 466.2278.

9-Ethyl-3-(3-(phenylethynyl)pyridin-2-yl)-9*H*-carbazole (6) was synthesized in a similar manner as descibed in [1]. A stirred mixture of 3-(3-bromopyridin-2-yl)-9-ethyl-9*H*-carbazole **4b** (92 mg, 0.25 mmol), $Pd(PPh_3)_2Cl_2$ (18 mg, 0.025 mmol), CuI (2 mg, 0.01 mmol), *i*-Pr_2NH (0.5 mL) and DMSO (2.5 mL) was heated at 80 °C for 20 min under argon. A solution of phenylacetylene (93 mg, 0.1 mL, 0.75 mmol) in *i*-Pr_2NH (1 mL) was then added by portions for 1 h. The reaction mixture was stirred at 80 °C for 24 h, evaporated without heating to remove *i*-Pr_2NH, treated with H_2O (50 mL) and extracted with CH_2Cl_2 (3 × 15 mL). The extract was dried over Na_2SO_4 and evaporated to dryness. The residue was purified by flash column chromatography on silica gel (3.5 × 30 cm) with CH_2Cl_2 as the eluent. The fraction with R_f 0.2 and violet fluorescence gave compound **6** (69 mg, 74%). Compound **6** was obtained as a yellow brown oil. ^{1}H NMR (250 MHz, $CDCl_3$): δ = 1.55 (t, J = 7.2 Hz, 3 H), 4.50 (q, J = 7.2 Hz, 2 H), 7.27–7.37

(m, 5 H), 7.48–7.60 (m, 5 H), 8.04 (dd, J = 7.8, 1.7 Hz, 1 H), 8.19 (d, J = 7.8 Hz, 1 H), 8.30 (dd, J = 8.6, 1.7 Hz, 1 H), 8.76 (dd, J = 4.8, 1.7 Hz, 1 H), 9.01 (d, J = 1.4 Hz, 1 H) ppm. ^{13}C NMR (62.9 MHz, $CDCl_3$): δ = 13.9, 37.8, 88.5, 94.4, 108.0, 108.7, 117.5, 119.2, 120.6, 120.7, 121.8, 122.6, 123.1, 123.4, 125.7, 127.5, 128.4, 128.5, 130.2, 131.5, 140.4(9), 140.5(2), 141.0, 148.6, 160.2 ppm. HRMS (ESI): MH^+, found 373.1711. $C_{27}H_{21}N_2$ requires 373.1699.

1-Ethyl-7-iodo-6-phenyl-1*H*-carbazolo[3,4-*a*]phenazine (7a) was synthesized in a similar manner as descibed in [1]. To a stirred suspension of 9-ethyl-3-(3-(phenylethynyl)quinoxalin-2-yl)-9*H*-carbazole **2a** (85 mg, 0.2 mmol) in dry CH_3CN (17 mL) a solution of ICl (33 mg, 0.2 mmol) in dry CH_3CN (2 mL) was added. The reaction mixture was kept at room temperature for 24 h in the dark. The yellow orange needles precipitate of **7a** (48 mg) was filtered off and washed on the filter with CH_3CN (2 mL). The filtrate was then evaporated to dryness. The residue was extracted with CH_2Cl_2 (15 mL) and saturated $Na_2S_2O_3$ solution (5 mL). The organic layer was separated and dried over Na_2SO_4 and purified by flash column chromatography on silica gel (2.5 × 55 cm) with CH_2Cl_2 as the eluent. The bright yellow orange fraction with R_f 0.85 gave 24 mg of cyclization product **7a**. Total yield was 72 mg (65%). 1-Ethyl-7-iodo-6-phenyl-1*H*-carbazolo[3,4-*a*]phenazine **7a** was obtained as yellow orange needles with mp 248–250 °C (EtOH). ^{1}H NMR (250 MHz, $CDCl_3$): δ = 1.50 (t, J = 7.2 Hz, 3 H), 4.52 (q, J = 7.2 Hz, 2 H), 6.29 (d, J = 8.4 Hz, 1 H), 6.63 (ddd, J = 8.2, 7.0, 1.2 Hz, 1 H), 7.27–7.30 (m, 1 H), 7.37 (d, J = 7.9 Hz, 1 H), 7.43–7.52 (m, 3 H), 7.63–7.66 (m, 2 H), 7.82–7.92 (m, 2 H), 7.95 (d, J = 9.0 Hz, 1 H), 8.38–8.47 (m, 2 H), 9.81 (d, J = 9.0 Hz, 1 H) ppm. ^{13}C NMR (62.9 MHz, $CDCl_3$) δ 13.8, 37.8, 107.9, 110.8, 112.2, 118.0, 118.8, 123.3, 124.4, 124.8, 125.4, 128.5, 128.8, 128.9, 129.5, 129.6, 129.9, 130.6, 132.5, 139.5, 141.5, 141.6, 142.5, 142.6, 143.0 (2C), 146.4, 149.0 ppm. HRMS (ESI): MH^+, found 550.0785. $C_{30}H_{21}IN_3$ requires 550.0775. $M+Na^+$, found 572.0584. $C_{30}H_{20}IN_3Na$ requires 550.0594.

When using a 1.5-fold excess of ICl, a hardly separable mixture of **7a** and its 4-iodo derivative **8a** in a 7.7:1 ratio was obtained.

7-Ethyl-13-iodo-12-phenyl-7*H*-quinoxalino[5,6-*c*]carbazole (7b) was synthesized in a similar manner as descibed in [1]. To a stirred suspension of 9-ethyl-3-(3-(phenylethynyl)pyrazin-2-yl)-9*H*-carbazole **2b** (59 mg, 0.16 mmol) in dry CH_3CN (2 mL) a solution of ICl (15 mg, 0.09 mmol) in dry CH_3CN (1.5 mL) was added. After 1 h, the next portion of the solution of ICl (5 mg, 0.03 mmol) in dry CH_3CN (1.5 mL) was added. The stirred

reaction mixture was kept at room temperature for 20 h in the dark and then evaporated to dryness. The residue was extracted with CH_2Cl_2 (30 mL) and saturated $Na_2S_2O_3$ solution (5 mL). The organic layer was separated and dried over Na_2SO_4. The extract was purified by flash column chromatography on silica gel (2 × 25 cm) with ethyl acetate - petroleum ether (1:3, v/v) as the eluent. The yellow fraction with R_f 0.45 gave 46 mg (75%) of cyclization product **7b**. 7-Ethyl-13-iodo-12-phenyl-7*H*-quinoxalino[5,6-*c*]carbazole **7b** was obtained as yellow needles with mp 217–219 ^{o}C (EtOH). ^{1}H NMR (250 MHz, $CDCl_3$): δ = 1.49 (t, *J* = 7.2 Hz, 3 H), 4.51 (q, *J* = 7.2 Hz, 2 H), 6.20 (d, *J* = 8.5 Hz, 1 H), 6.67 (ddd, *J* = 8.3, 7.0, 1.2 Hz, 1 H), 7.31 (ddd, *J* = 8.0, 7.0, 0.9 Hz, 1 H), 7.42 (d, *J* = 7.9 Hz, 1 H), 7.48–7.65 (m, 5 H), 7.98 (d, *J* = 9.0 Hz, 1 H), 8.90 (d, *J* = 1.8 Hz, 1 H), 8.97 (d, *J* = 1.8 Hz, 1 H), 9.62 (d, *J* = 9.0 Hz, 1 H) ppm. ^{13}C NMR (62.9 MHz, $CDCl_3$): δ = 13.9, 37.7, 107.9, 111.3, 111.7, 116.8, 118.8, 123.4, 123.6, 124.5, 125.3, 126.0, 128.6, 128.8, 130.0, 132.5, 139.1, 140.4, 141.3, 141.7, 143.9, 144.0, 146.7, 147.4 ppm. HRMS (ESI): MH^+, found 500.0625. $C_{26}H_{19}IN_3$ requires 500.0618.

When using equimolar amount of ICl, a hardly separable mixture of **7b** and its 4-iodo derivative **8b** in a 10:1 ratio (69% total yield) was obtained.

7-Ethyl-10,13-diiodo-12-phenyl-7*H*-quinoxalino[5,6-*c*]carbazole (8b): To a suspension of 7-ethyl-13-iodo-12-phenyl-7*H*-quinoxalino[5,6-*c*]carbazole **7b** (25 mg, 0.05 mmol) in dry CH_3CN (4 mL), a solution of ICl (24 mg, 0.15 mmol) in dry CH_3CN (2 mL) was added. The reaction mixture was stirred at room temperature for 24 h in the dark. After evaporation in air without heating the residue was extracted with CH_2Cl_2 (30 mL) and saturated $Na_2S_2O_3$ solution (5 mL). The extract dried over Na_2SO_4 and purified by flash column chromatography on silica gel (2 × 25 cm) using a mixture of ethyl acetate - petroleum ether (1:3, v/v) as the eluent. The cyclization product **8b** (30 mg, 97%) was isolated from the yellow fraction with R_f 0.5. Compound **8b** was obtained as a yellow solid with mp 254–255 ^{o}C (EtOH). ^{1}H NMR (250 MHz, $CDCl_3$): δ = 1.51 (t, *J* = 7.2 Hz, 3 H), 4.53 (q, *J* = 7.2 Hz, 2 H), 6.44 (d, *J* = 1.4 Hz, 1 H), 7.18 (d, *J* = 8.7 Hz, 1 H), 7.55–7.62 (m, 5 H), 7.66–7.75 (m, 1 H), 7.97 (d, *J* = 9.0 Hz, 1 H), 8.92 (d, *J* = 1.8 Hz, 1 H), 8.99 (d, *J* = 1.8 Hz, 1 H), 9.66 (d, *J* = 9.0 Hz, 1 H) ppm. ^{13}C NMR (62.9 MHz, $CDCl_3$): δ = 13.8, 37.8, 83.0, 109.9, 111.3, 111.9, 115.8, 124.4, 125.1, 126.4, 128.9, 129.9, 130.0, 132.1, 133.1, 133.5, 138.2, 140.4, 141.1, 141.6, 144.0, 144.2, 145.9, 147.2 ppm. HRMS (ESI): MH^+, found 625.9576. $C_{26}H_{18}I_2N_3$ requires 625.9585.

7-Ethyl-13-iodo-12-phenyl-7*H*-quinolino[8,7-*c*]carbazole (7c) was synthesized in a similar manner as descibed in **[1].** To a stirred suspension of 9-ethyl-3-(3-(phenylethynyl)pyridin-2-yl)-9*H*-carbazole **6** (74 mg, 0.2 mmol) in dry CH_3CN (6 mL) a solution of ICl (52 mg, 0.3 mmol) in dry CH_3CN (3 mL) was added. The reaction mixture was kept at room temperature for 24 h in the dark. The yellow orange needles precipitate of **7c** (51 mg) was filtered off and washed on the filter with CH_3CN (2 mL). The filtrate was then evaporated to dryness. The residue was extracted with CH_2Cl_2 (20 mL) and saturated $Na_2S_2O_3$ (5 mL). The organic layer was separated and dried over Na_2SO_4 and purified by flash column chromatography on silica gel (2.5 × 25 cm) with CH_2Cl_2 as the eluent. The yellow fraction with R_f 0.7 gave 11 mg of cyclization product **7c**. Total yield of **7c** was 62 mg (62%). Compound **7c** was obtained as yellow needles with mp 196–197 °C. ^{1}H NMR (250 MHz, $CDCl_3$): δ = 1.55 (t, J = 7.0 Hz, 3 H), 4.58 (q, J = 7.0 Hz, 2 H), 6.25 (d, J = 8.4 Hz, 1 H), 6.68 (t, J = 7.6 Hz, 1 H), 7.28–7.34 (m, 1 H), 7.44 (d, J = 8.0 Hz, 1 H), 7.52–7.65 (m, 6 H), 7.99 (d, J = 9.0 Hz, 1 H), 8.81 (d, J = 8.2 Hz, 1 H), 9.01 (d, J = 3.4 Hz, 1 H), 9.78 (d, J = 9.0 Hz, 1 H) ppm. ^{13}C NMR (62.9 MHz, $CDCl_3$): δ = 13.9, 37.7, 107.8, 108.6, 110.9, 116.5, 118.5, 122.0, 123.6, 124.0, 124.1, 125.2, 126.8, 127.3, 128.5, 128.6, 129.9, 132.8, 139.0, 141.4, 142.8, 143.7, 146.4, 147.7, 149.4 ppm. HRMS (ESI): MH^+, found 499.0681. $C_{27}H_{19}IN_2$ requires 499.0666.

1-Ethyl-6-phenyl-7-(*p*-tolylethynyl)-1*H*-carbazolo[3,4-*a*]phenazine (9a): A stirred mixture of 1-ethyl-7-iodo-6-phenyl-1*H*-carbazolo[3,4-*a*]phenazine **7a** (110 mg, 0.2 mmol), $Pd(PPh_3)_2Cl_2$ (14 mg, 0.02 mmol), CuI (2 mg, 0.01 mmol), Et_3N (3 mL) and dry THF (5 mL) was heated at 85 °C for 20 min under argon. A solution of *p*-tolylacetylene (35 mg, 0.3 mmol) and Et_3N (2 mL) in dry THF (2 mL) was then added by portions for 3 h. The reaction mixture was stirred for total 24 h at 85 °C. After subsequent evaporation, the residue was treated with H_2O (100 mL) and extracted with CH_2Cl_2 (2 × 20 mL). The extract was dried over Na_2SO_4 and concentrated. Flash column chromatography on silica gel (3.5 × 55 cm) was then carried out using CH_2Cl_3 as the eluent. The yellow orange fraction with R_f 0.6 (yellow fluorescence under UV 356 nm) gave 90 mg (84%) of compound **9a**. The product was then heated with hexane (3 mL) for crystallization and filtered off. 1-Ethyl-7-iodo-6-phenyl-1*H*-carbazolo[3,4-*a*]phenazine **9a** was obtained as orange needles with orange fluorescence in the solid state under UV (356 nm) and mp 214–217 °C (ethyl acetate - petroleum ether, 1:3, v/v). ^{1}H NMR (250 MHz, $CDCl_3$): δ = 1.54 (t, J = 7.1 Hz, 3 H), 2.41 (s, 3 H), 4.56 (q, J = 7.1 Hz, 2 H), 6.42 (d, J = 8.4 Hz, 1 H), 6.70 (ddd, J = 8.0, 6.7, 1.0 Hz, 1 H), 7.19 (d, J = 7.9 Hz, 2 H), 7.27–7.34 (m, 1 H), 7.36–7.45 (m, 3 H), 7.46–7.55 (m, 3 H), 7.82–8.00 (m, 5 H), 8.39–8.44 (m, 1 H),

8.46–8.50 (m, 1 H), 9.81 (d, *J* = 8.9 Hz, 1 H) ppm. ^{13}C NMR (62.9 MHz, $CDCl_3$): δ = 13.8, 21.7, 37.7, 87.8, 100.1, 107.9, 110.7, 118.5, 118.8, 120.9, 121.6, 123.4, 123.9, 124.7, 125.5, 125.6, 128.2, 128.4, 129.0, 129.2, 129.3, 129.6, 130.0, 130.1, 131.7, 131.9, 138.4, 139.5, 141.5, 141.8, 142.2, 142.4, 142.5, 142.6, 146.3 ppm. HRMS (ESI): MH^+, found 538.2265. $C_{39}H_{28}N_3$ requires 538.2278.

7-Ethyl-12-phenyl-13-(*p*-tolylethynyl)-7*H*-quinoxalino[5,6-*c*]carbazole (9b): A stirred mixture of 7-ethyl-13-iodo-12-phenyl-7*H*-quinoxalino[5,6-*c*]carbazole **7b** (50 mg, 0.1 mmol), $PdCl_2(PPh_3)_2$ (7 mg, 0.01 mmol), CuI (2 mg, 0.01 mmol), Et_3N (3 mL) was heated at 85 °C for 20 min under argon. A solution of *p*-tolylacetylene (23 mg, 0.2 mmol) in Et_3N (1.6 mL) was then dropped for 3 h. The reaction mixture was stirred at 85 °C for total 24 h. followed by evaporation. The residue was then diluted with H_2O (50 mL) and extracted with $CHCl_3$ (3 × 10 mL). The extract was dried over Na_2SO_4. Flash column chromatography on silica gel (2 × 20 cm) was carried out using a mixture of ethyl acetate - petroleum ether (1:3, v/v) as the eluent. Compound **9b** (40 mg, 82%) was isolated from the yellow fraction with R_f 0.3 (yellow-green fluorescence under UV 356 nm). Compound **9b** was synthesized as yellow plates with green fluorescence in the solid state under UV (356 nm) and mp 152–154 °C (hexane). ^{1}H NMR (250 MHz, $CDCl_3$): δ = 1.54 (t, *J* = 7.2 Hz, 3 H), 2.38 (s, 3 H), 4.57 (q, *J* = 7.2 Hz, 2 H), 6.28 (d, *J* = 8.5 Hz, 1 H), 6.69 (ddd, *J* = 8.1, 7.3, 0.8 Hz, 1 H), 7.14 (d, *J* = 7.9 Hz, 2 H), 7.28–7.36 (m, 3 H), 7.42–7.53 (m, 4 H), 7.78–7.82 (m, 2 H), 7.99 (d, *J* = 9.0 Hz, 1 H), 9.03 (dd, *J* = 4.1, 1.9 Hz, 2 H), 9.59 (d, *J* = 9.0 Hz, 1 H) ppm. ^{13}C NMR (62.9 MHz, $CDCl_3$): δ = 14.0, 21.6, 37.8, 88.5, 101.2, 108.0, 111.6, 117.3, 118.9, 120.3, 121.4, 123.1, 123.3, 124.6, 125.4, 125.7, 128.4, 128.6, 129.0, 129.6, 131.9, 132.0, 138.9, 139.2, 140.6, 141.4, 141.6, 141.9, 143.4, 144.0, 145.6 ppm. HRMS (ESI): MH^+, found 488.2099. $C_{35}H_{26}N_3$ requires 488.2121. $M+Na^+$, found 510.1916. $C_{35}H_{25}N_3Na$ requires 510.1946.

7-Ethyl-12-phenyl-13-(*p*-tolylethynyl)-7*H*-quinolino[8,7-*c*]carbazole (9c): Compound **9c** was obtained similarly to **9a** starting from **7c** (50 mg, 0.1 mmol). The reaction was carried out at 80–82 °C. Flash column chromatography was carried out on silica gel (3.5 × 45 cm) with CH_2Cl_2 as the eluent. The yellow fraction with R_f 0.6 and light blue fluorescence under UV (356 nm) gave compound **9c** (43 mg, 88%) as yellow prisms (CH_2Cl_2) with mp 210–212 °C (EtOH). ^{1}H NMR (250 MHz, $CDCl_3$): δ = 1.53 (t, *J* = 7.2 Hz, 3 H), 2.40 (s, 3 H), 4.56 (q, *J* = 7.2 Hz, 2 H), 6.36 (d, *J* = 8.3 Hz, 1 H), 6.68

(ddd, J = 8.3, 7.0, 1.1 Hz, 1 H), 7.18 (d, J = 7.9 Hz, 2 H), 7.25–7.35 (m, 3 H), 7.42 (d, J = 8.1 Hz, 1 H), 7.46–7.52 (m, 3 H), 7.63 (dd, J = 8.2, 4.4 Hz, 1 H), 7.77–7.81 (m, 2 H), 7.96 (d, J = 9.1 Hz, 1 H), 8.98 (dd, J = 8.2, 1.7 Hz, 1 H), 9.10 (dd, J = 4.4, 1.7 Hz, 1 H), 9.74 (d, J = 9.1 Hz, 1 H) ppm. ^{13}C NMR (62.9 MHz, $CDCl_3$): δ = 13.9, 21.6, 37.7, 87.6, 99.1, 107.9, 111.0, 117.1, 118.6, 119.8, 120.4, 121.1, 123.5, 123.6, 124.1, 124.5 (2C), 125.4, 128.0, 128.2, 129.2, 129.7, 131.3, 132.1, 135.6, 138.6 (2C), 139.2, 141.3, 141.4, 142.2, 148.7 ppm. HRMS (ESI): MH^+, found 487.2180. $C_{36}H_{27}N_2$ requires 487.2174.

8-Ethyl-17-*p*-tolyl-8*H*-carbazolo[3,4-*a*]naphtho[1,2-*c*]phenazine (10a) was synthesized in a similar manner as descibed in [1, compound **14c**]. To a solution of compound **9a** (54 mg, 0.1 mmol) in CH_2Cl_2 (10 mL) CF_3SO_3H (0.1 mL) was added. The dark red reaction mixture was kept at room temperature for 24 h in the dark. Then it was mixed with saturated aqueous K_2CO_3 (50 mL)and separated in a separating funnel.. The yellow CH_2Cl_2 phase was dried over Na_2SO_4 and purified by flash column chromatography on silica gel (2 × 50 cm) with CH_2Cl_2 as the eluent. The bright yellow fraction with R_f 0.9 gave the cyclization product (50 mg, 92 %). Compound **10a** was obtained as a yellow orange solid with mp 294–295 °C. ^{1}H NMR (250 MHz, $CDCl_3$): δ = 1.61 (t, J = 7.2 Hz, 3 H), 2.58 (s, 3 H), 4.62 (q, J = 7.2 Hz, 2 H), 6.71 (d, J = 8.1 Hz, 1 H), 6.81 (t, J = 7.4 Hz, 1 H), 7.18 (d, J = 7.8 Hz, 1 H), 7.39 (d, J = 7.6 Hz, 1 H), 7.46–7.52 (m, 4 H), 7.77 (d, J = 7.8 Hz, 2 H), 7.81–7.90 (m, 2 H), 7.94 (d, J = 8.9 Hz, 1 H), 8.20 (d, J = 8.3 Hz, 1 H), 8.32–8.39 (m, 3 H), 9.48 (s, 1 H), 9.66 (d, J = 8.9 Hz, 1 H) ppm. ^{13}C NMR (62.9 MHz, $CDCl_3$): δ = 14.0, 21.4, 37.9, 108.2, 109.6, 118.0, 120.2, 122.7, 123.4, 123.8, 124.9, 125.3, 125.4, 125.7, 126.4, 126.9, 127.1, 128.8, 129.0, 129.1, 129.2 (2C), 129.4, 129.5, 129.6, 130.3, 130.7, 132.4, 137.3, 137.8, 140.0, 140.7, 141.7, 141.8, 141.9, 142.2, 143.8 ppm. UV-vis (CH_2Cl_2), λ_{max} nm (lg ε): 264 (4.75), 303 (4.72), sh 324 (4.59), 357 (4.36), 374 (4.46), 433 (4.23), end absorption up to 507 nm. HRMS (ESI): MH^+, found 538.2285. $C_{39}H_{28}N_3$ requires 538.2278.

7-Ethyl-16-*p*-tolyl-7*H*-naphtho[1',2';7,8]quinoxalino[5,6-*c*]carbazole (10b): Compound **10b** was obtained similarly to **10a** starting from 7-ethyl-12-phenyl-13-(*p*-tolylethynyl)-7*H*-quinoxalino[5,6-*c*]carbazole **9b** (49 mg, 0.1 mmol). Flash column chromatography was carried out on silica gel (2 × 40 cm) with CH_2Cl_2 as the eluent. The yellow fraction with R_f 0.3 gave the cyclization product **10b** (40 mg, 82%) as a yellow orange solid with mp 225–227 °C (hexane). ^{1}H NMR (250 MHz, $CDCl_3$): δ = 1.61 (t, J = 7.2 Hz, 3H), 2.56 (s, 3 H), 4.63 (q, J = 7.2 Hz, 2H), 6.73 (d, J = 7.9 Hz, 1H), 6.80–

6.86 (m, 1 H), 7.18 (ddd, *J* = 8.1, 7.2, 0.9 Hz, 1 H), 7.37–7.55 (m, 5 H), 7.76 (d, *J* = 7.9 Hz, 2 H), 7.98 (d, *J* = 8.9 Hz, 1 H), 8.21 (d, *J* = 8.2 Hz, 1 H), 8.37 (d, *J* = 8.4 Hz, 1 H), 8.90 (d, *J* = 2.0 Hz, 1 H), 8.94 (d, *J* = 2.0 Hz, 1 H), 9.30 (s, 1 H), 9.50 (d, *J* = 8.9 Hz, 1 H) ppm. ^{13}C NMR (62.9 MHz, $CDCl_3$): δ = 14.0, 21.4, 37.9, 108.3, 110.1, 118.0, 119.5, 121.8, 122.8, 123.4, 124.6, 125.1, 125.4, 125.7, 126.2, 126.4, 126.7, 127.6, 128.9, 129.0, 129.1, 130.3, 130.7, 131.8, 137.2, 137.7, 139.7, 140.1, 140.6, 141.3, 142.1, 142.7, 143.2 ppm. UV-vis (CH_2Cl_2), λ_{max} (lg ε): 294 (4.57), 323 (4.54), 359 (4.35), 397 (4.03), 418 nm (3.96). HRMS (ESI): MH^+, found 488.2120. $C_{35}H_{26}N_3$ requires 488.2121. $M+Na^+$, found 510.1929. $C_{35}H_{25}N_3Na$ requires 510.1946.

7-Ethyl-16-*p*-tolyl-7*H*-naphtho[2',1';5,6]quinolino[8,7-*c*]carbazole (10c) was synthesized in a similar manner as descibed in [1, compound **14a**]. A dark solution of 7-ethyl-12-phenyl-13-(*p*-tolylethynyl)-7*H*-quinolino[8,7-*c*]carbazole **9c** (49 mg, 0.1 mmol) in CF_3COOH (3 mL) was heated at 85 °C for 24 h. The reaction mixture was evaporated to dryness, treated with saturated K_2CO_3 (2 mL) and CH_2Cl_2 (20 mL), extracted in a separating funnel and separated. The organic phase was dried over Na_2SO_4 and purified by flash column chromatography on silica gel (2.5 × 30 cm) with CH_2Cl_2 as the eluent. The yellow fraction with R_f 0.5 gave cyclization product **10c** (46 mg, 94 %). Compound **10c** was obtained as a yellow orange solid with mp 223–225 °C (EtOH). ^{1}H NMR (250 MHz, $CDCl_3$): δ = 1.60 (t, *J* = 7.2 Hz, 3 H), 2.57 (s, 3 H), 4.62 (q, *J* = 7.2 Hz, 2 H), 6.68 (d, *J* = 7.9 Hz, 1 H), 6.80 (ddd, *J* = 7.9, 7.0, 0.8 Hz, 1 H), 7.16 (ddd, *J* = 8.2, 7.1, 1.1 Hz, 1 H), 7.38 (ddd, *J* = 8.0, 7.0, 1.1 Hz, 1 H), 7.46–7.58 (m, 5 H), 7.73 (d, *J* = 8.0 Hz, 2 H), 7.98 (d, *J* = 8.9 Hz, 1 H), 8.14 (d, *J* = 8.0 Hz, 1 H), 8.37 (d, *J* = 8.2 Hz, 1 H), 8.61 (s, 1 H), 8.96 (dd, *J* = 8.4, 1.4 Hz, 1 H), 9.03 (dd, *J* = 4.4, 1.5 Hz, 1 H), 9.61 (d, *J* = 8.9 Hz, 1 H) ppm. ^{13}C NMR (62.9 MHz, $CDCl_3$): δ = 14.1, 21.4, 37.9, 108.2, 109.8, 117.7, 119.3, 120.7, 120.8, 123.0, 123.3, 123.5, 124.7, 125.1, 125.7, 125.8, 125.9, 126.0, 126.1, 126.2, 127.4, 129.2, 129.3, 130.2, 130.7, 130.9, 131.1, 137.4, 137.9, 139.6, 140.2, 140.8, 147.7, 148.8 ppm. UV-vis (CH_2Cl_2), λ_{max} (lg ε): 265 (4.63), 285 (4.53), sh 306 (4.48), 324 (4.56), 371 (4.08), sh 388 (3.96), 411 nm (3.79). HRMS (ESI): MH^+ found 487.2175. $C_{36}H_{27}N_2$ requires 487.2169.

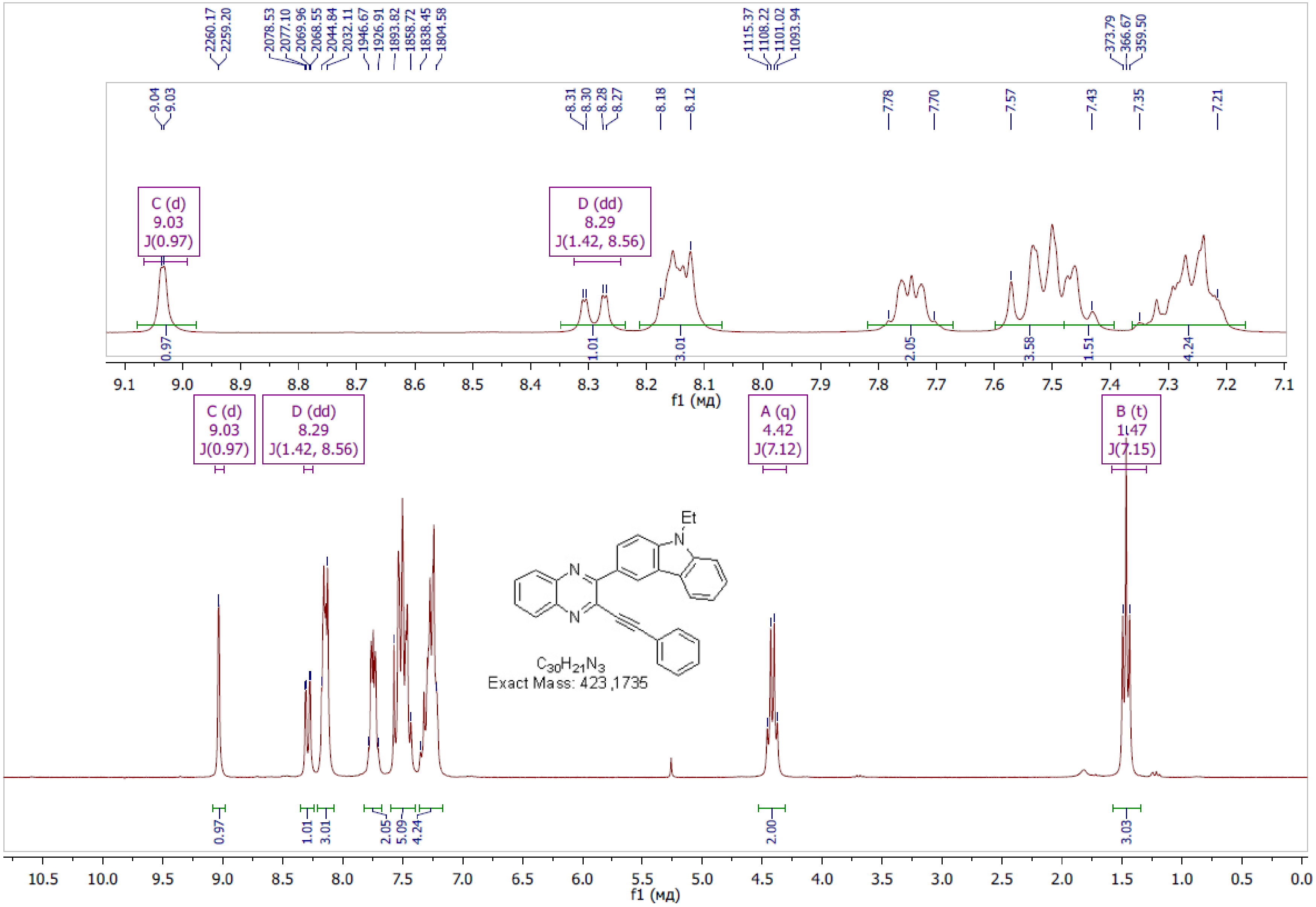

Figure S1. ^{1}H NMR spectrum of **2a** ($CDCl_3$, 250 MHz)

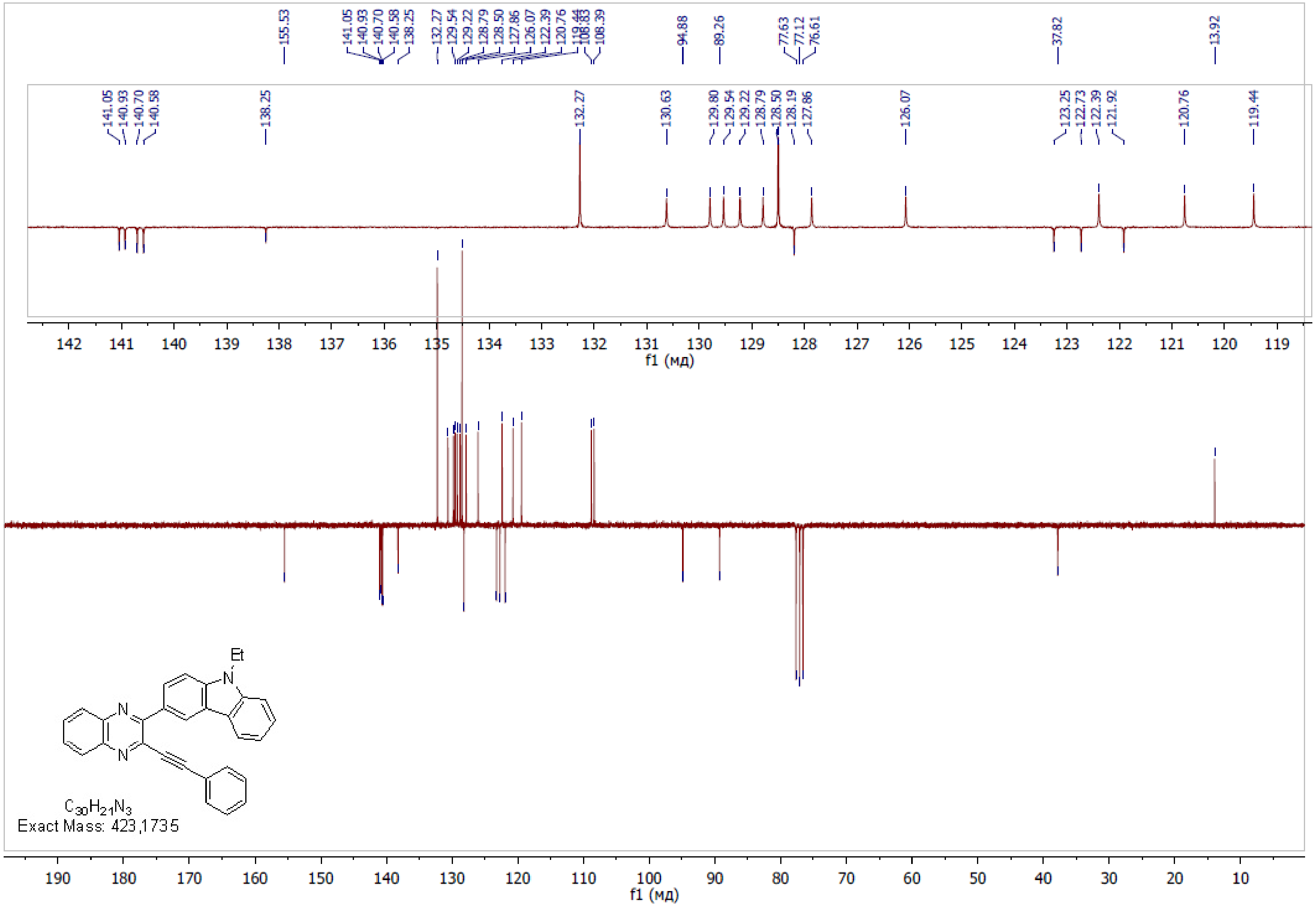

Figure S2. ^{13}C NMR APT spectrum of **2a** ($CDCl_3$, 62.9 MHz)

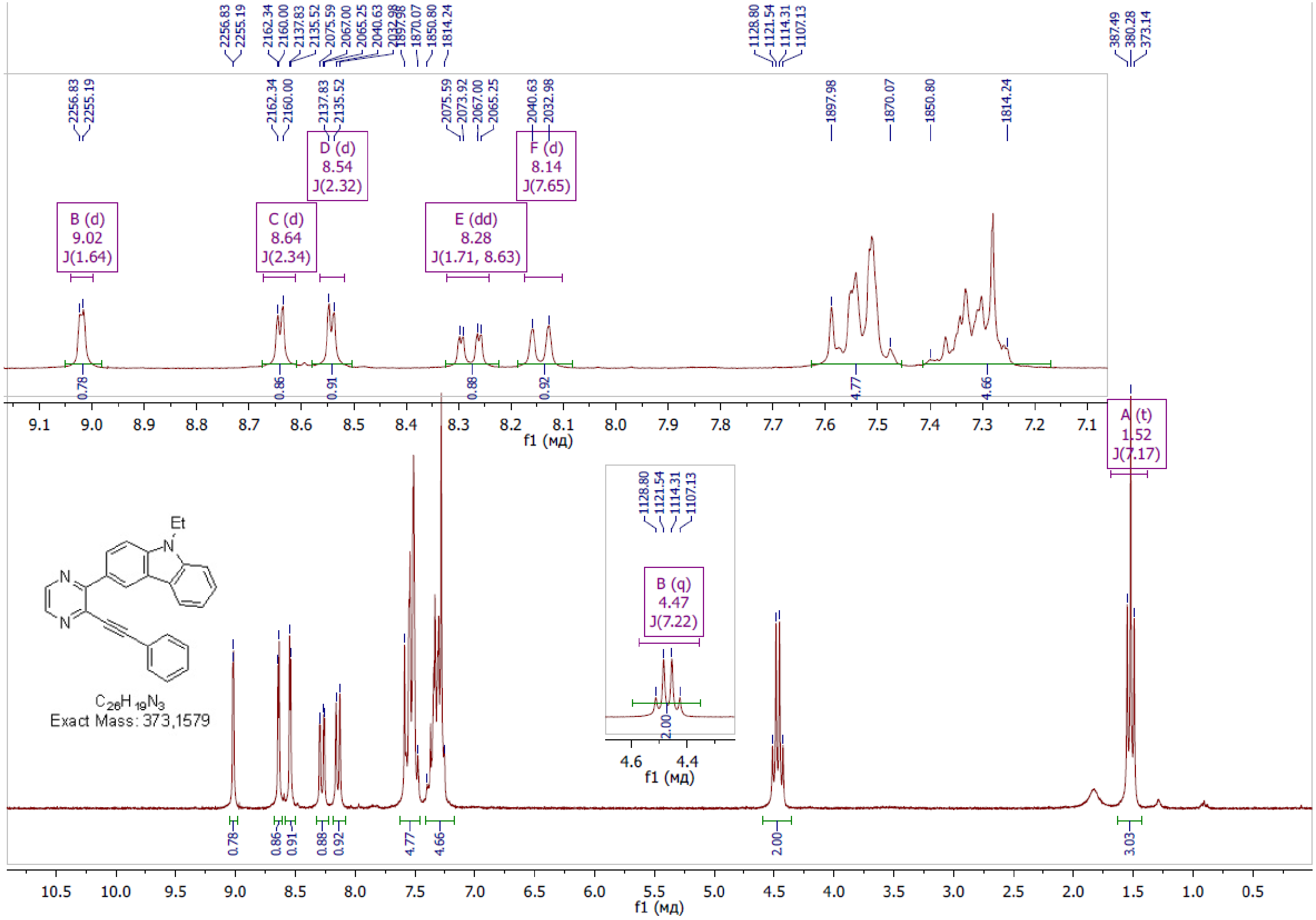

Figure S3. ^{1}H NMR spectrum of **2b** ($CDCl_3$, 250 MHz)

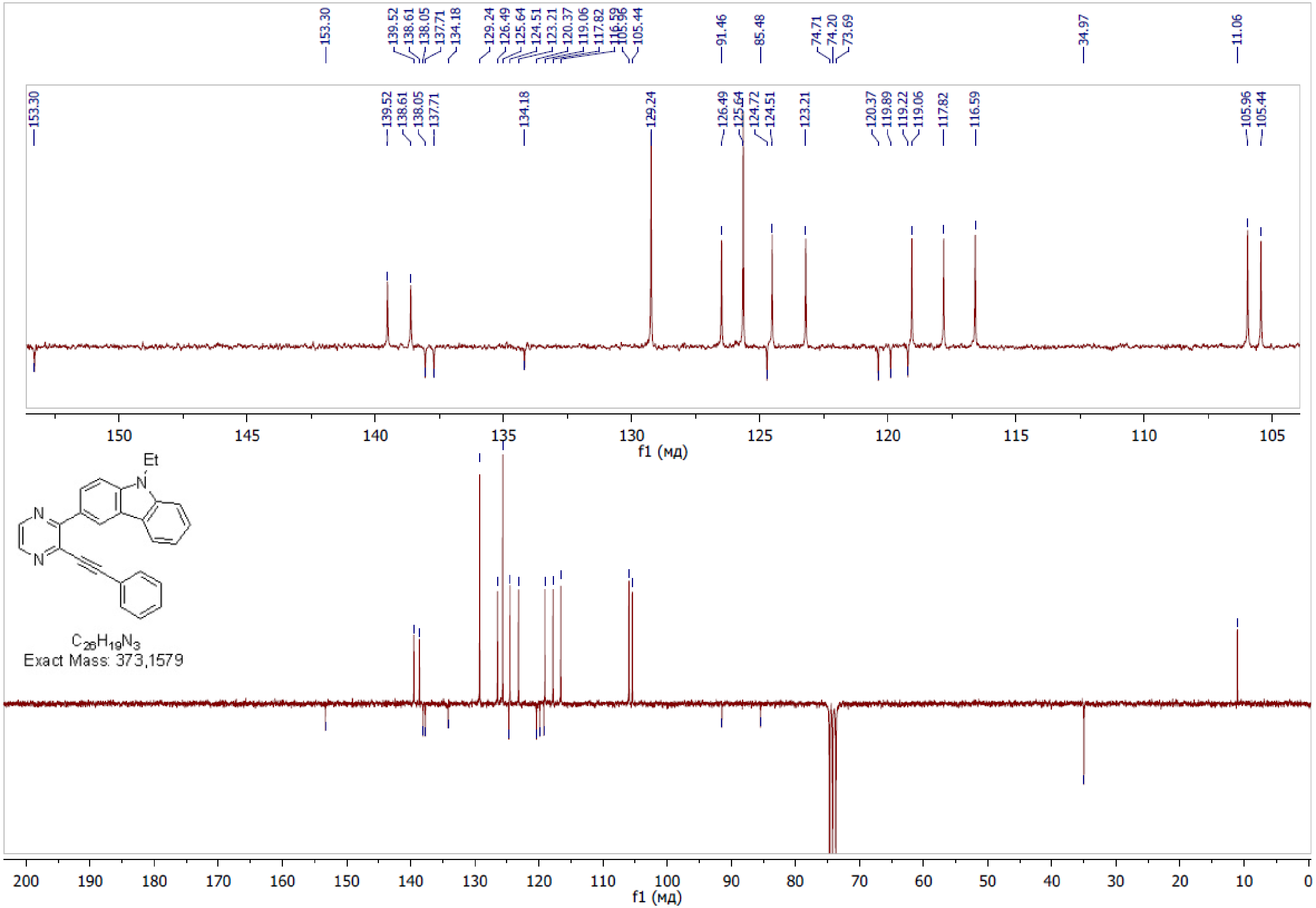

Figure S4. ^{13}C NMR APT spectrum of **2b** ($CDCl_3$, 62.9 MHz)

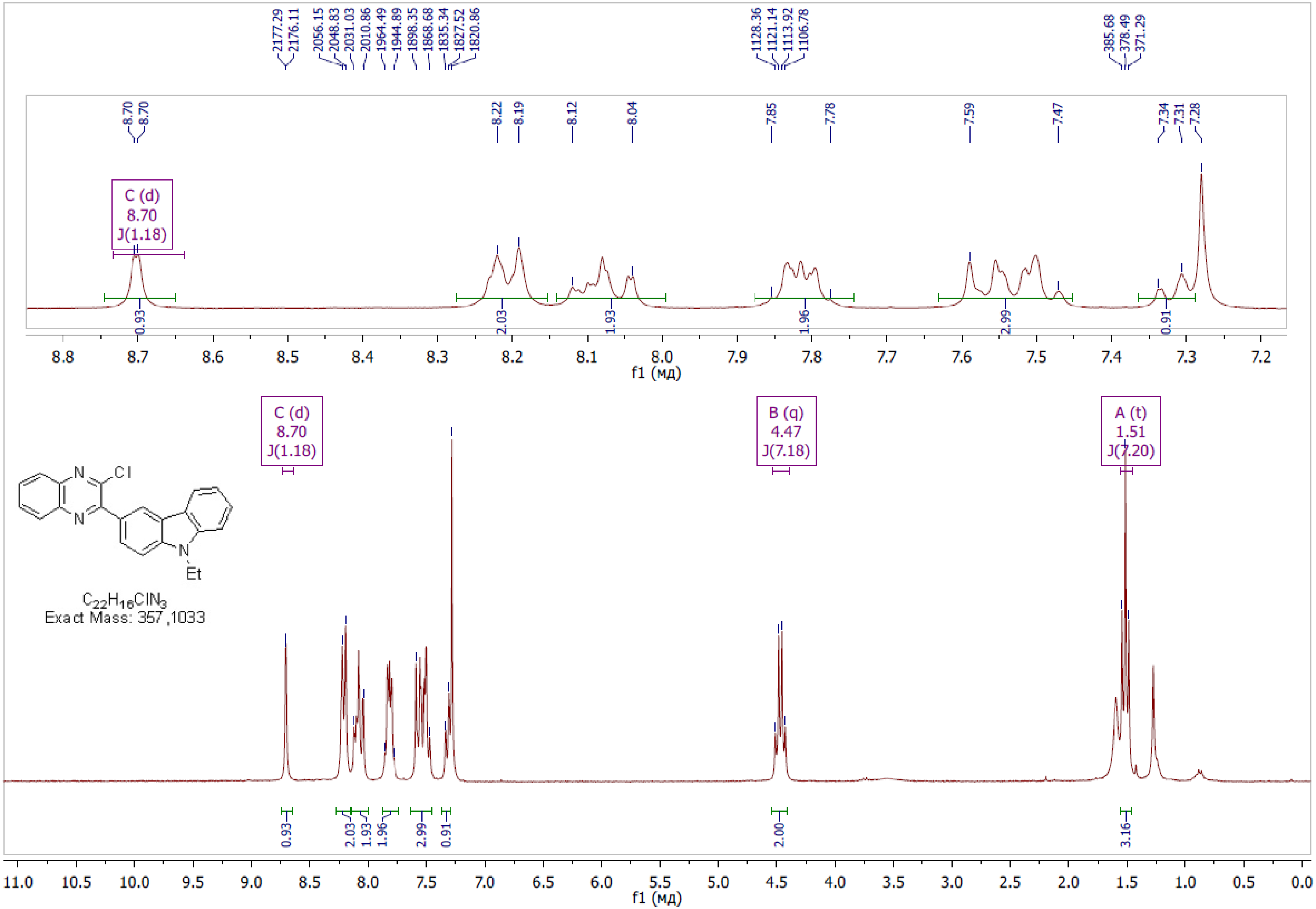

Figure S5. ^{1}H NMR spectrum of **4a** ($CDCl_3$, 250 MHz)

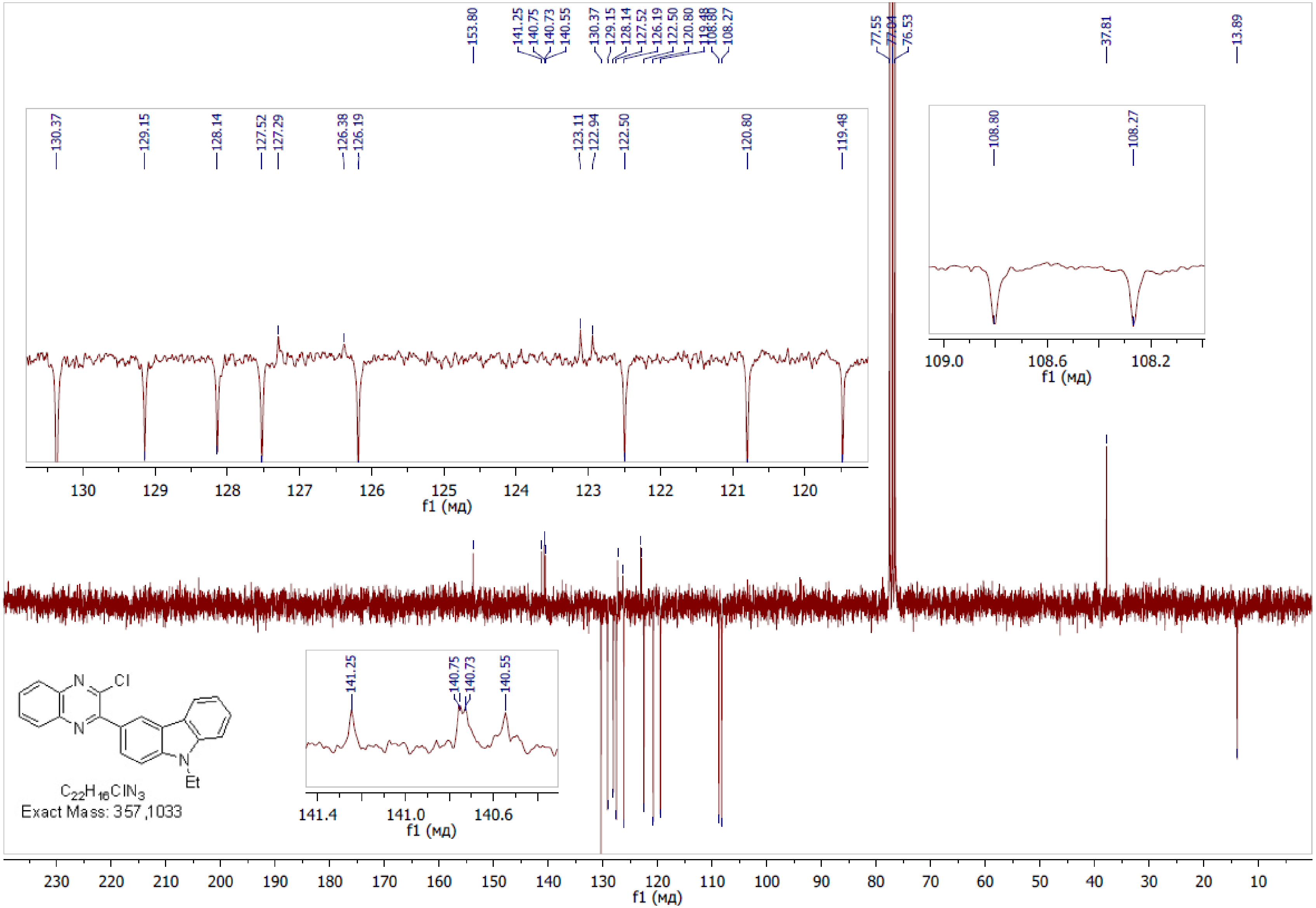

Figure S6. ^{13}C NMR APT spectrum of **4a** ($CDCl_3$, 62.9 MHz)

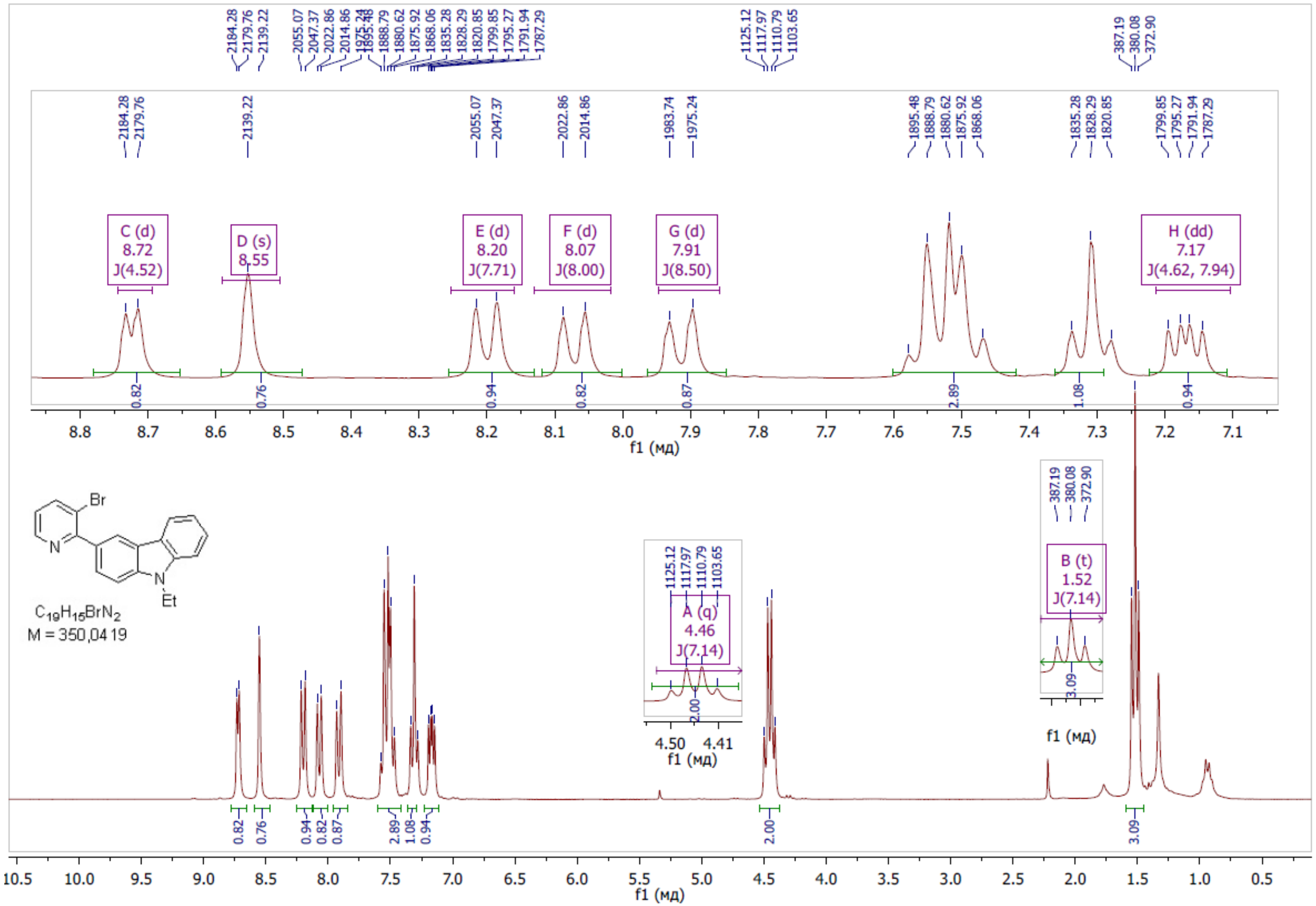

Figure S7. ^{1}H NMR spectrum of **4b** ($CDCl_3$, 250 MHz)

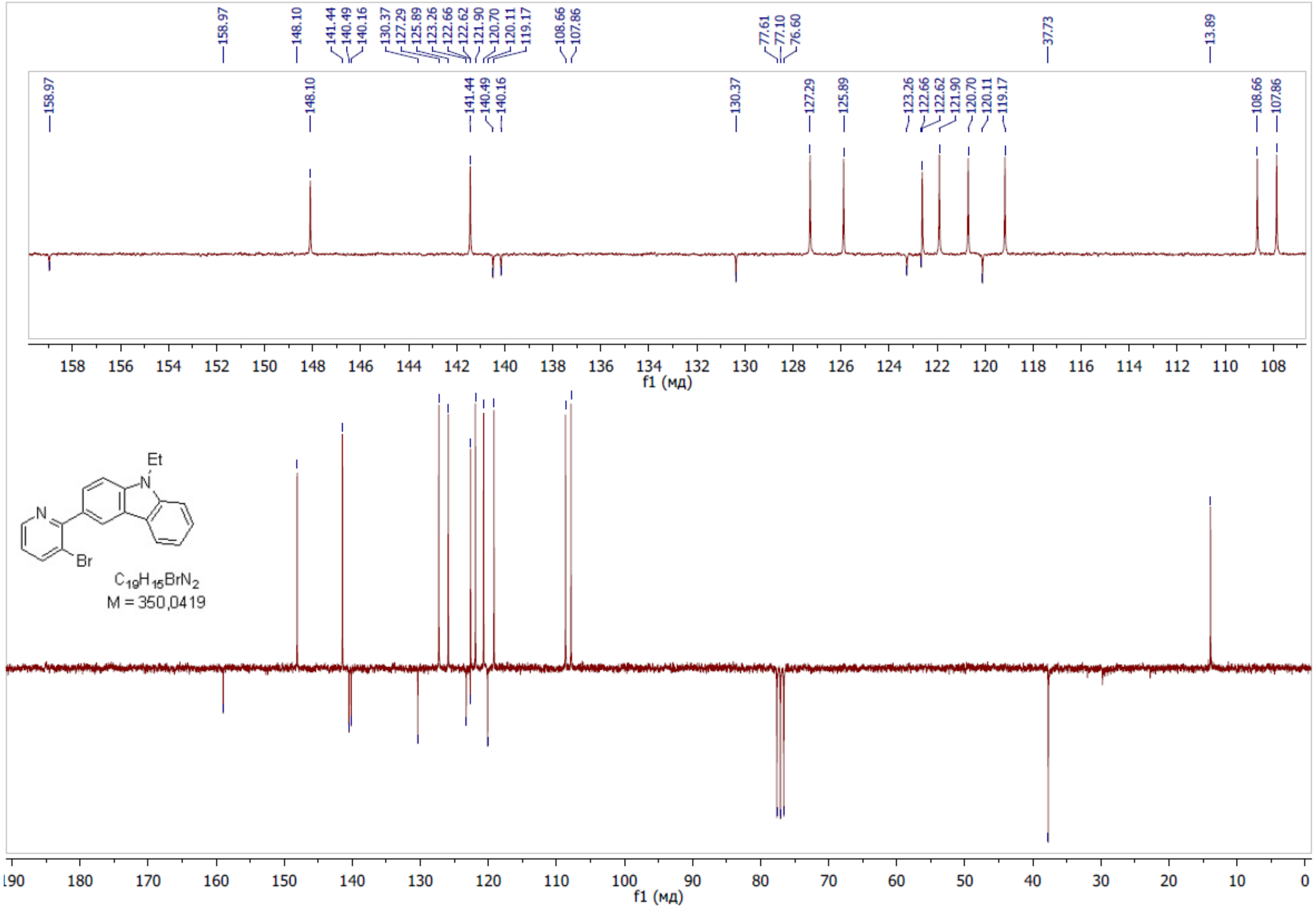

Figure S8. ^{13}C NMR APT spectrum of **4b** ($CDCl_3$, 62.9 MHz)

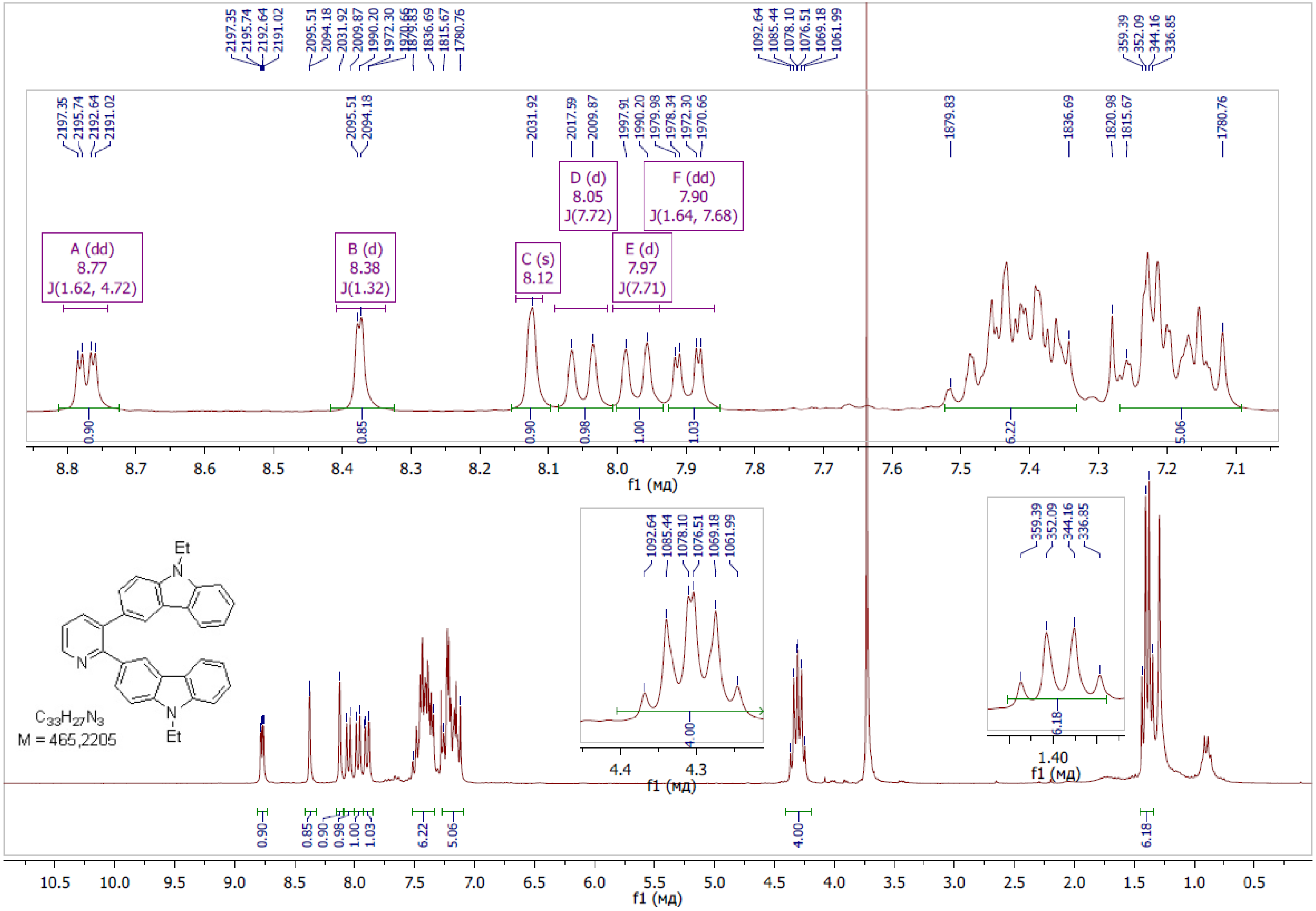

Figure S9. ^{1}H NMR spectrum of **5b** ($CDCl_3$, 250 MHz)

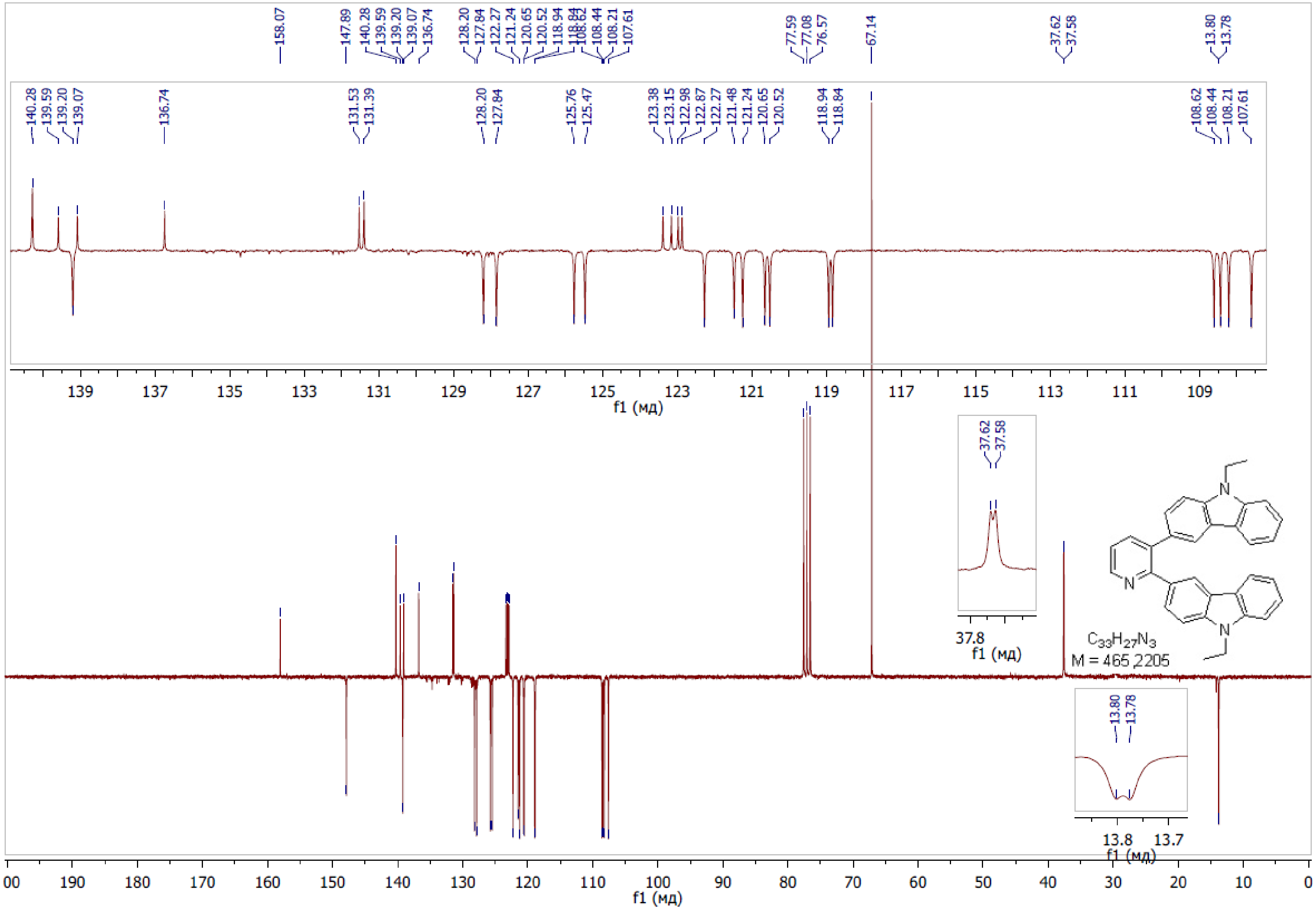

Figure S10. ^{13}C NMR APT spectrum of **5b** ($CDCl_3$, 62.9 MHz)

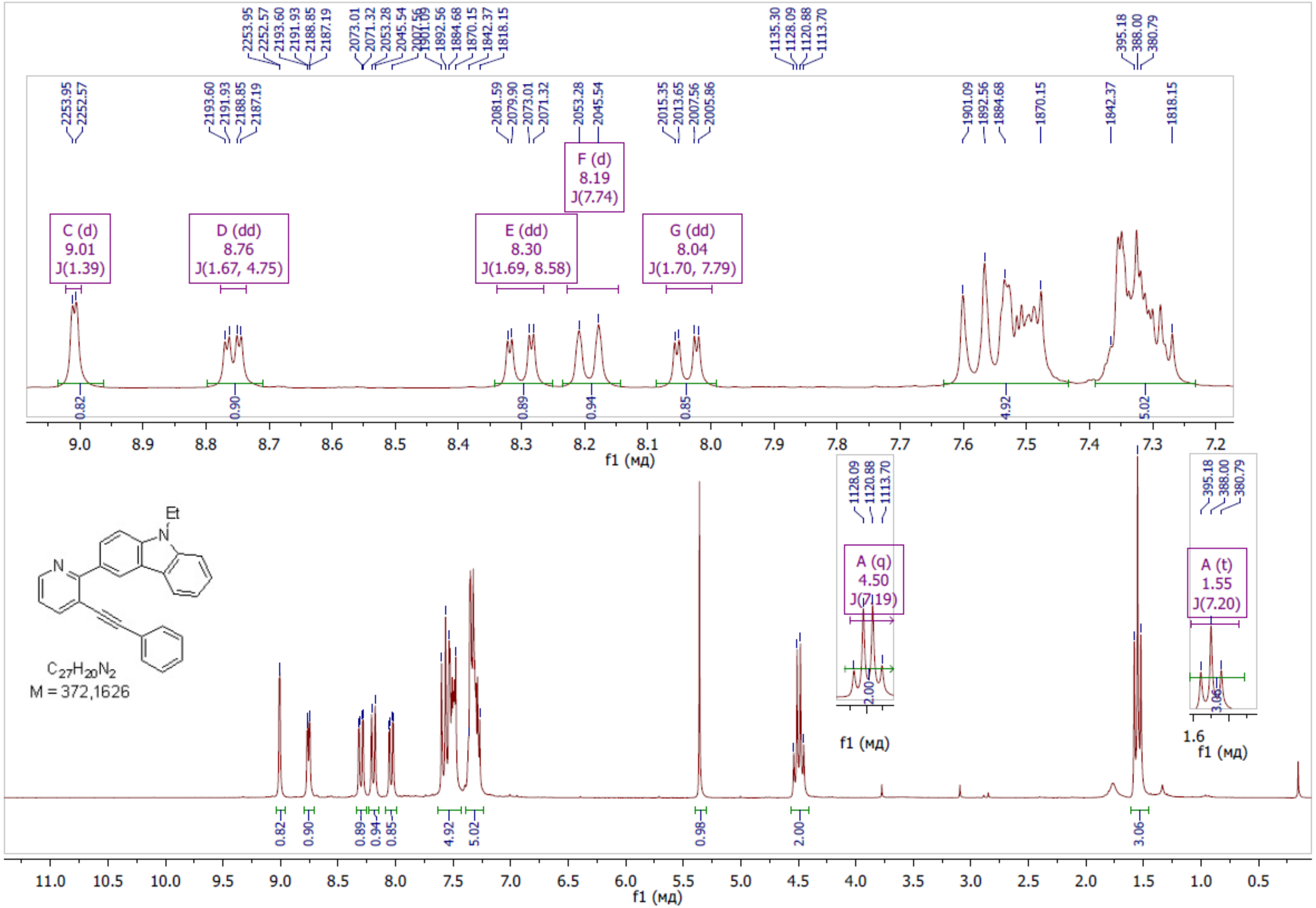

Figure S11. ^{1}H NMR spectrum of **6** ($CDCl_3$, 250 MHz)

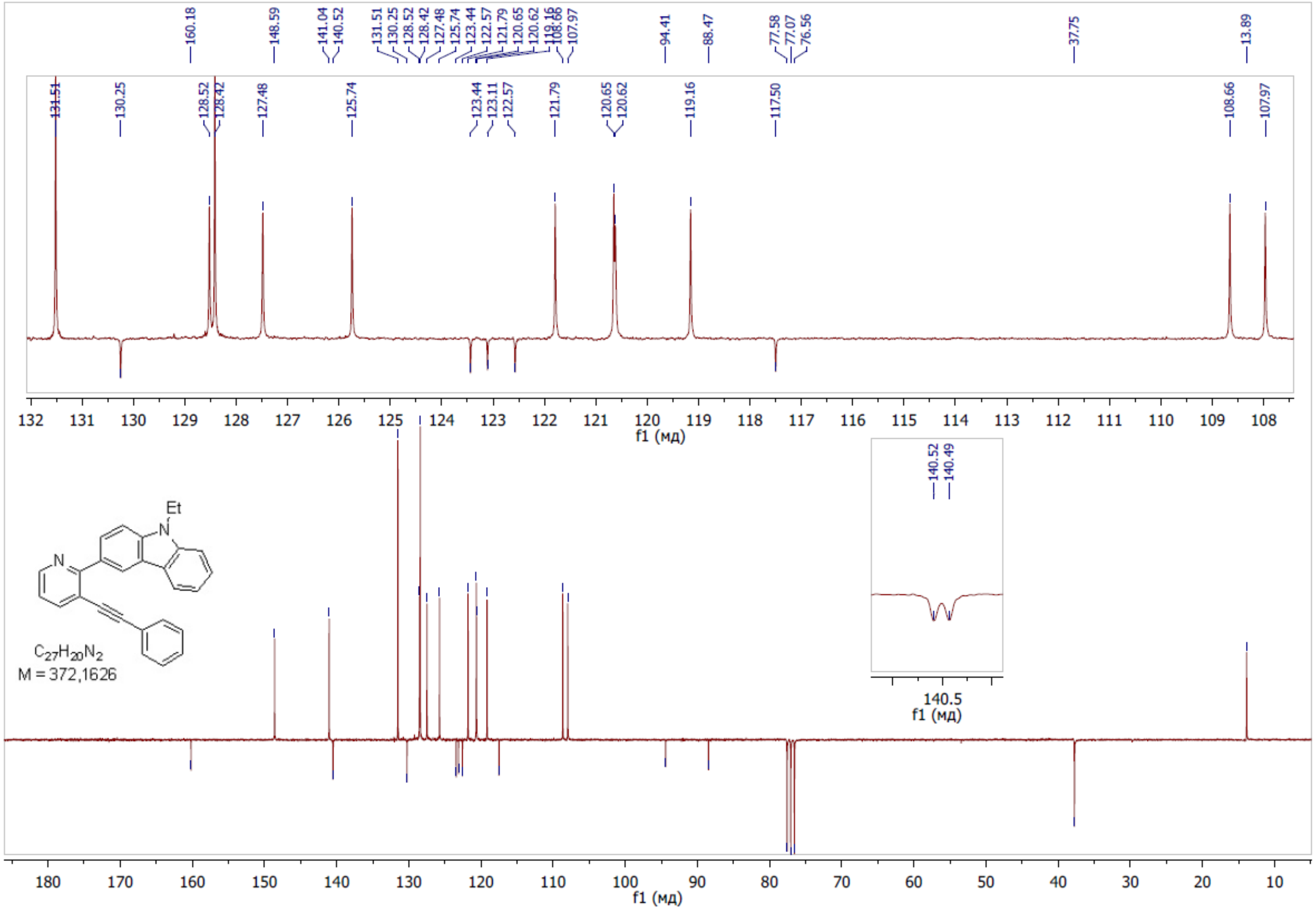

Figure S12. ^{13}C NMR APT spectrum of **6** ($CDCl_3$, 62.9 MHz)

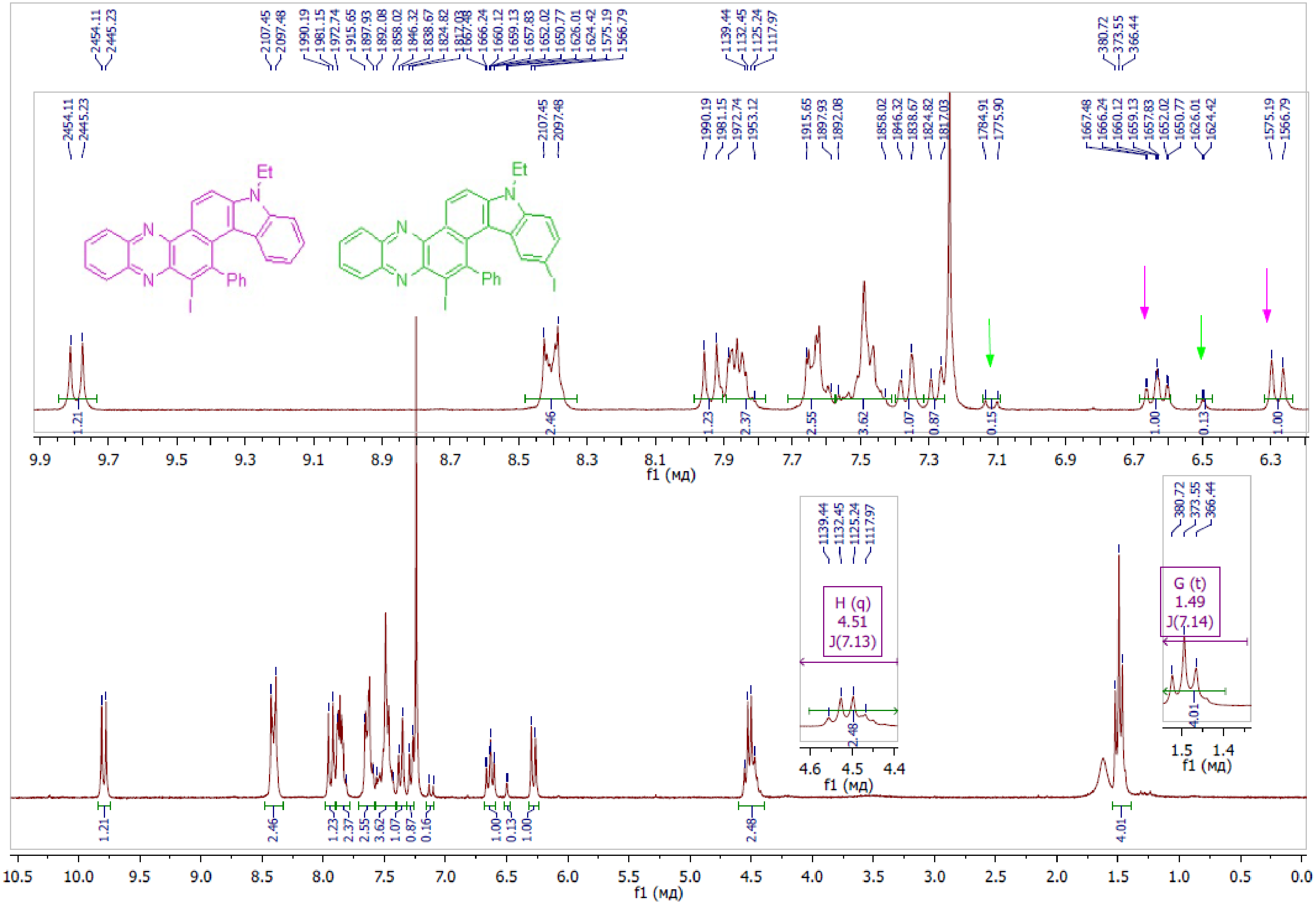

Figure S13. ^{1}H NMR spectrum of the mixture **7a** and **8a** ($CDCl_3$, 250 MHz)

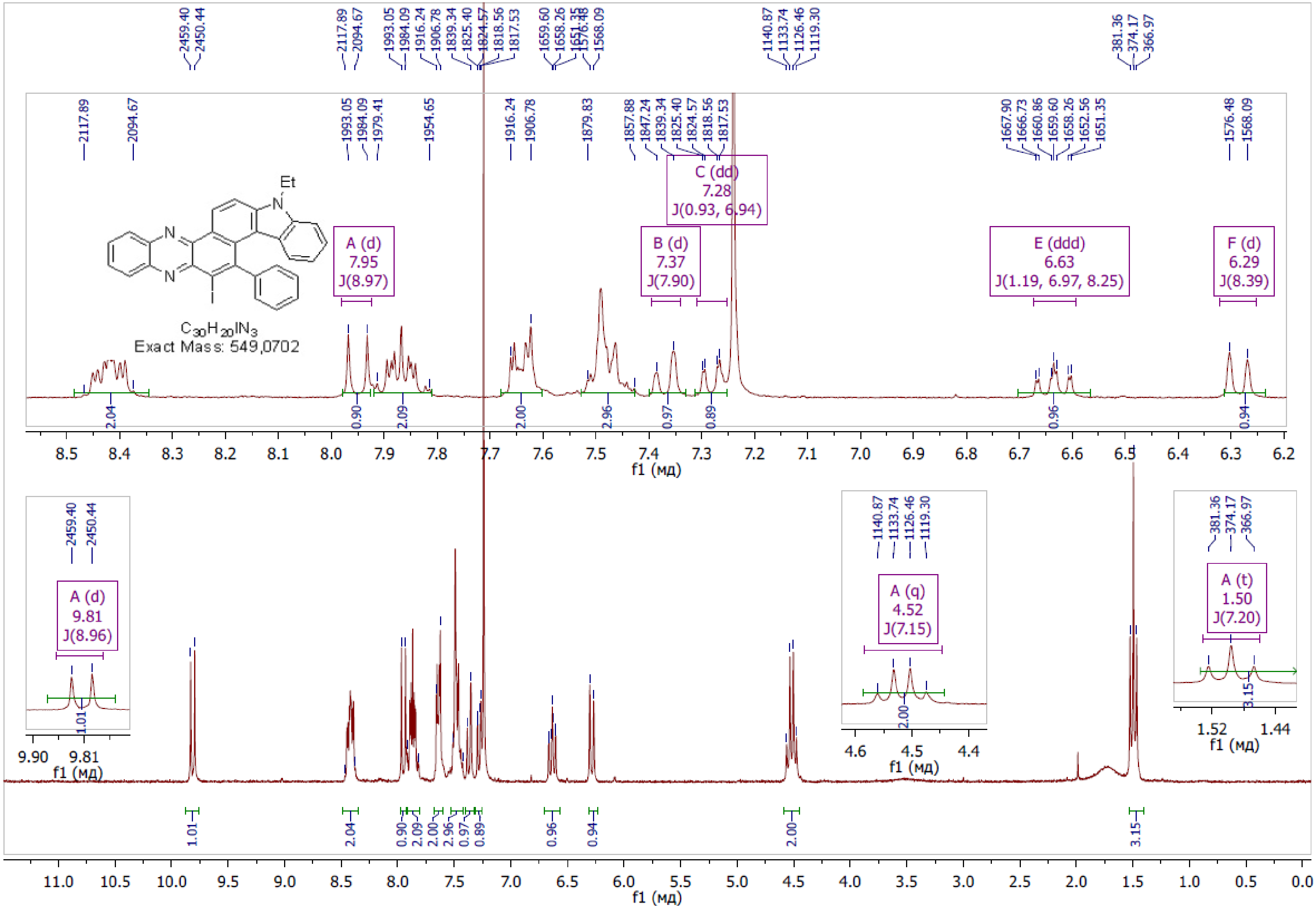

Figure S14. ^{1}H NMR spectrum of **7a** ($CDCl_3$, 250 MHz)

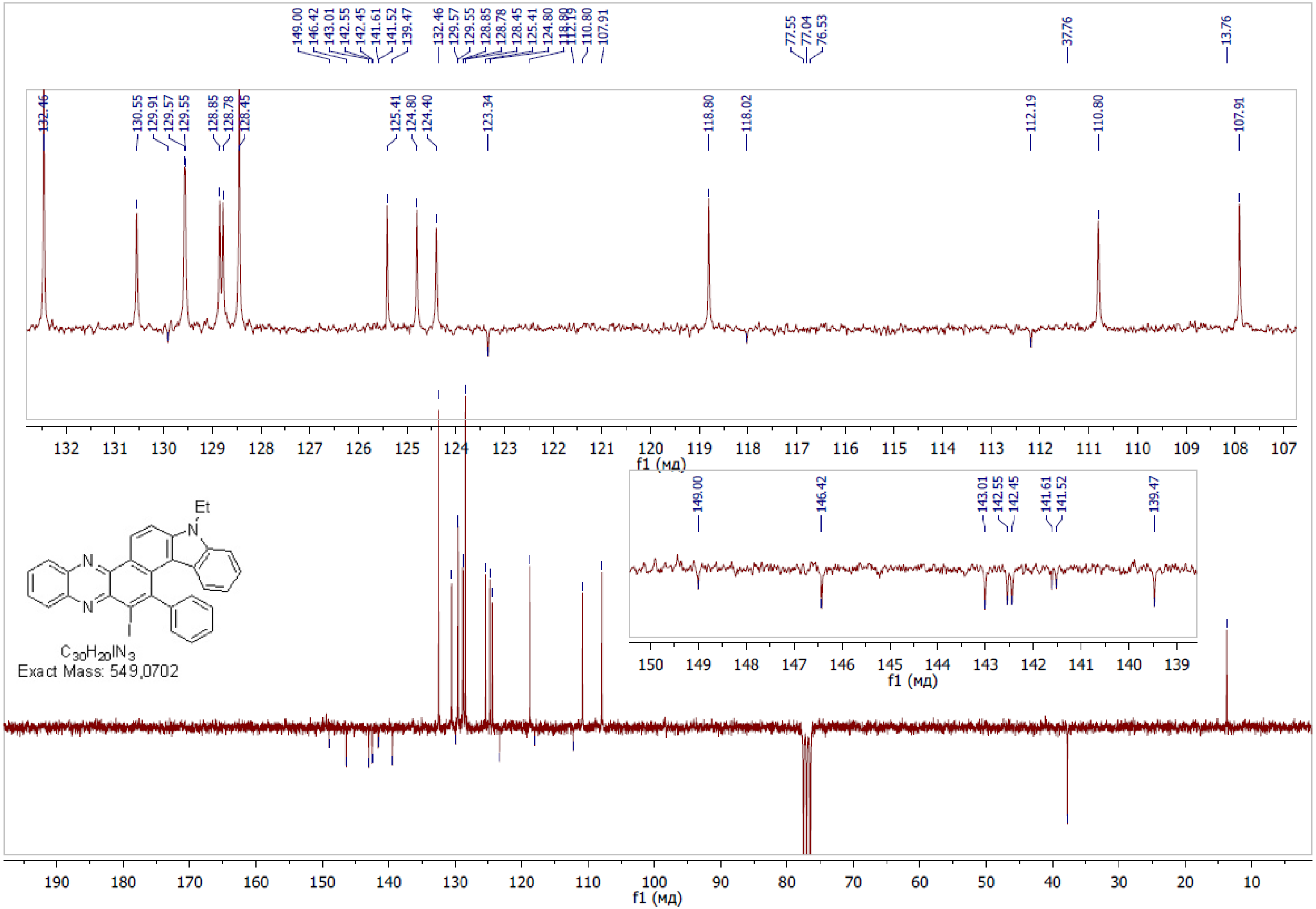

Figure S15. ^{13}C NMR APT spectrum of **7a** ($CDCl_3$, 62.9 MHz)

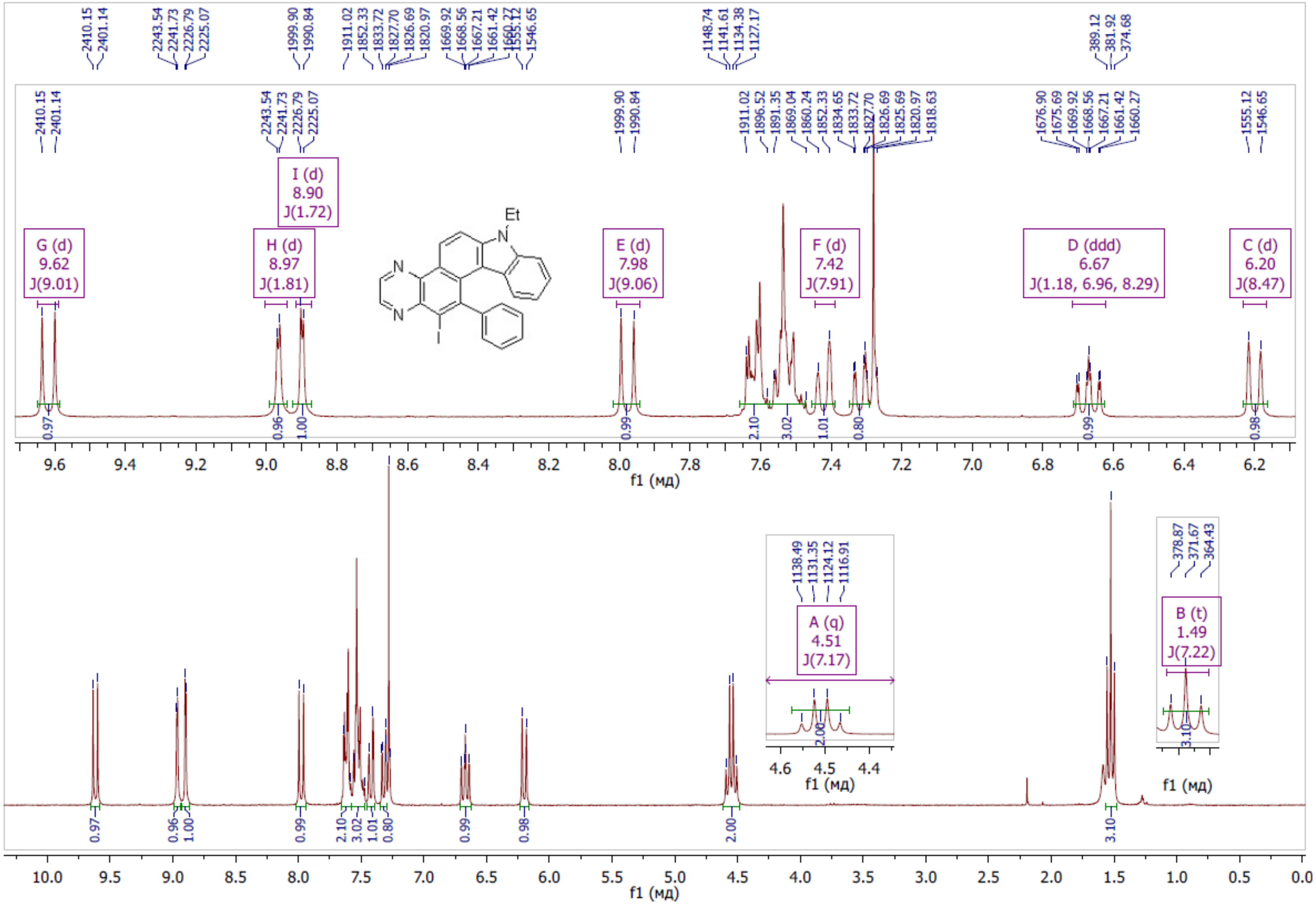

Figure S16. ^{1}H NMR spectrum of **7b** ($CDCl_3$, 250 MHz)

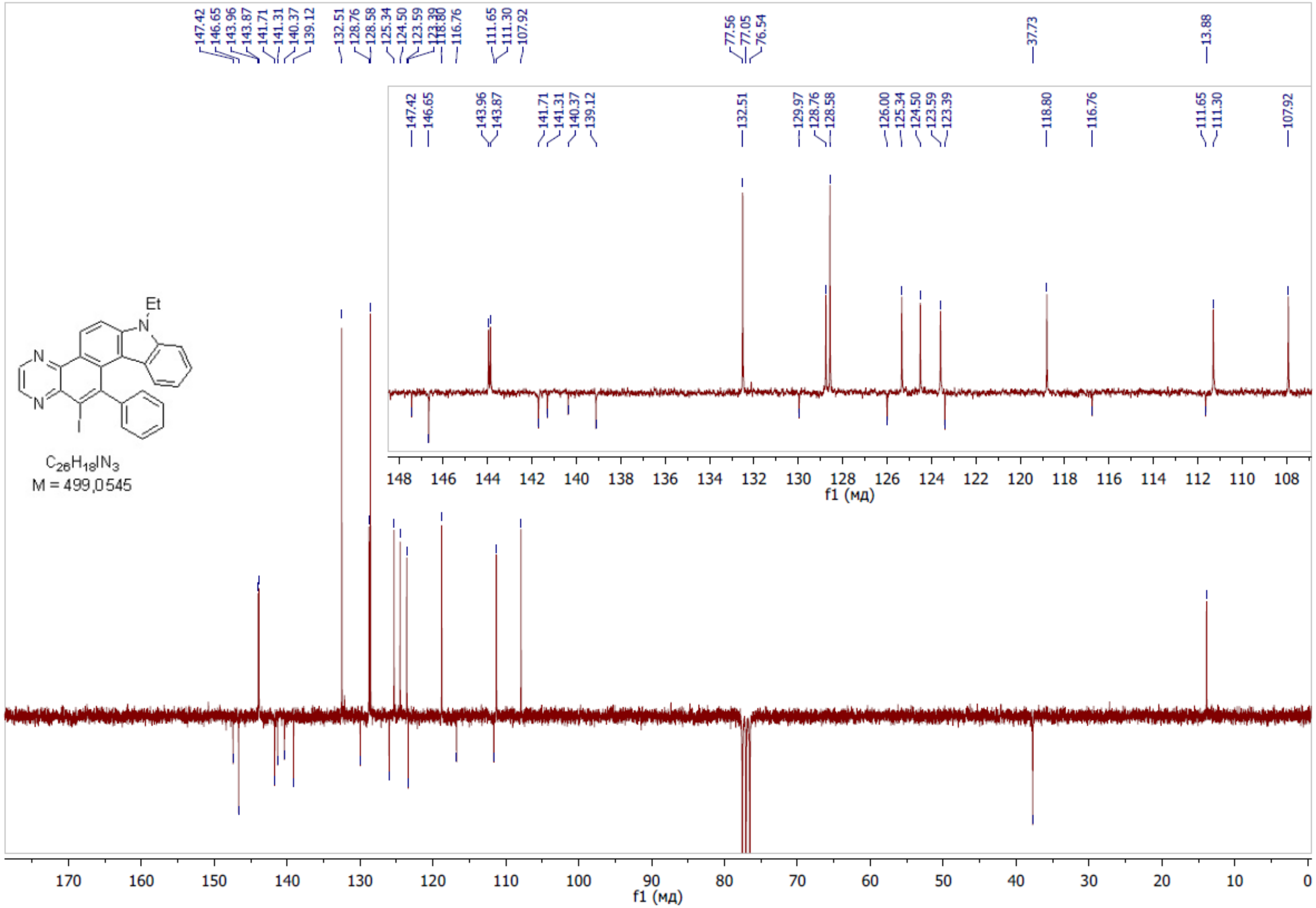

Figure S17. ^{13}C NMR APT spectrum of **7b** ($CDCl_3$, 62.9 MHz)

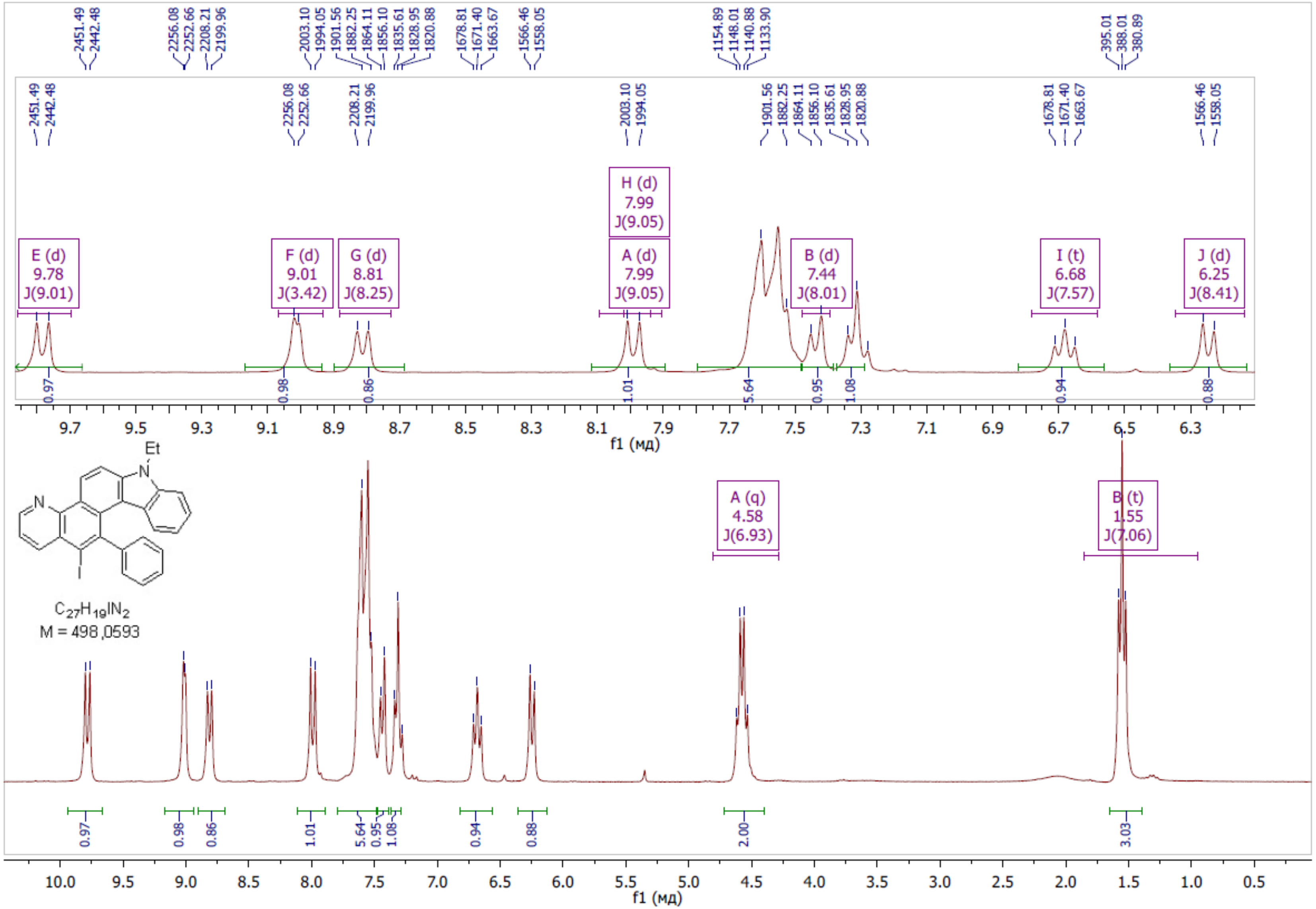

Figure S18. ^{1}H NMR spectrum of **7c** ($CDCl_3$, 250 MHz)

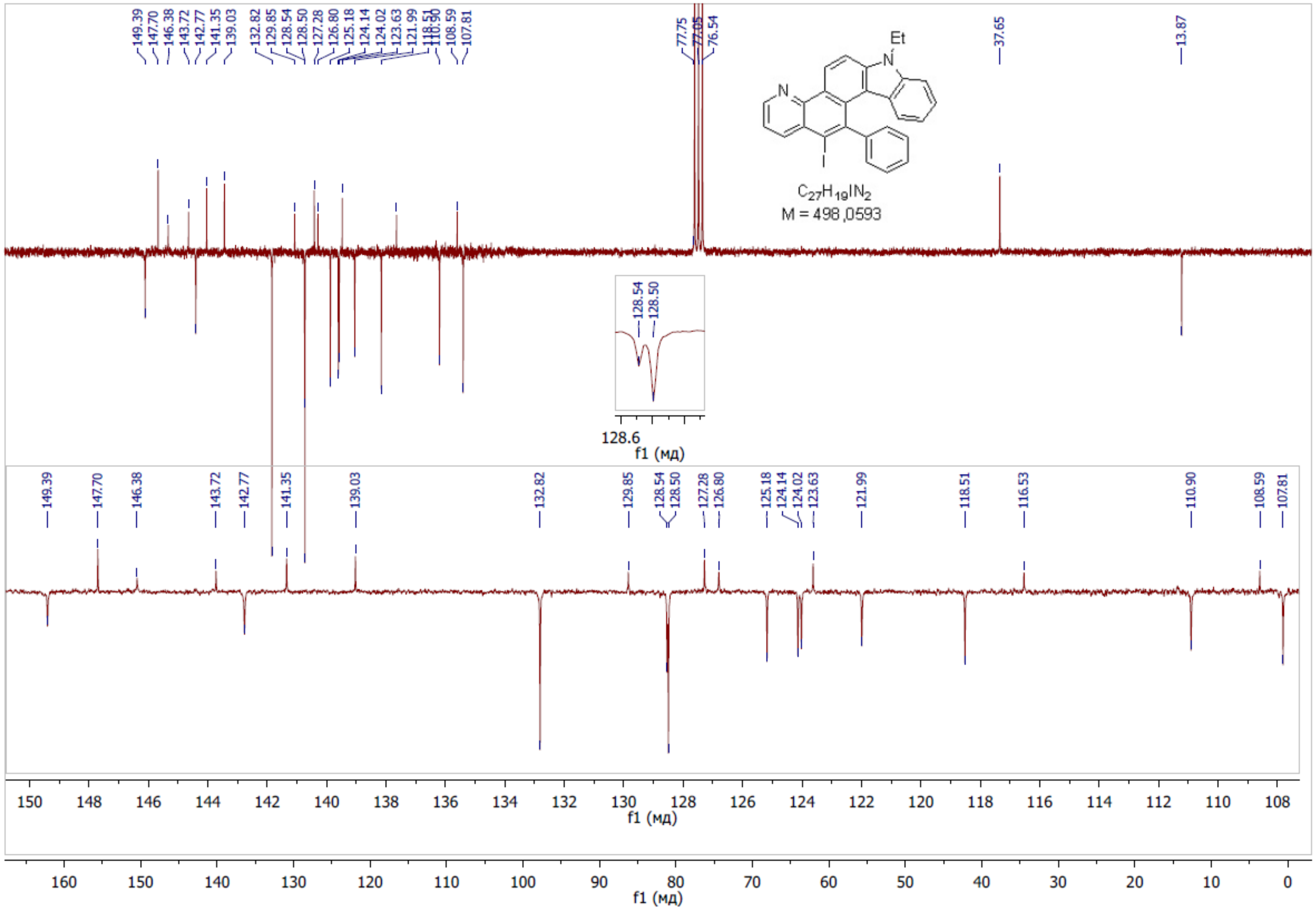

Figure S19. ^{13}C NMR APT spectrum of **7c** ($CDCl_3$, 62.9 MHz)

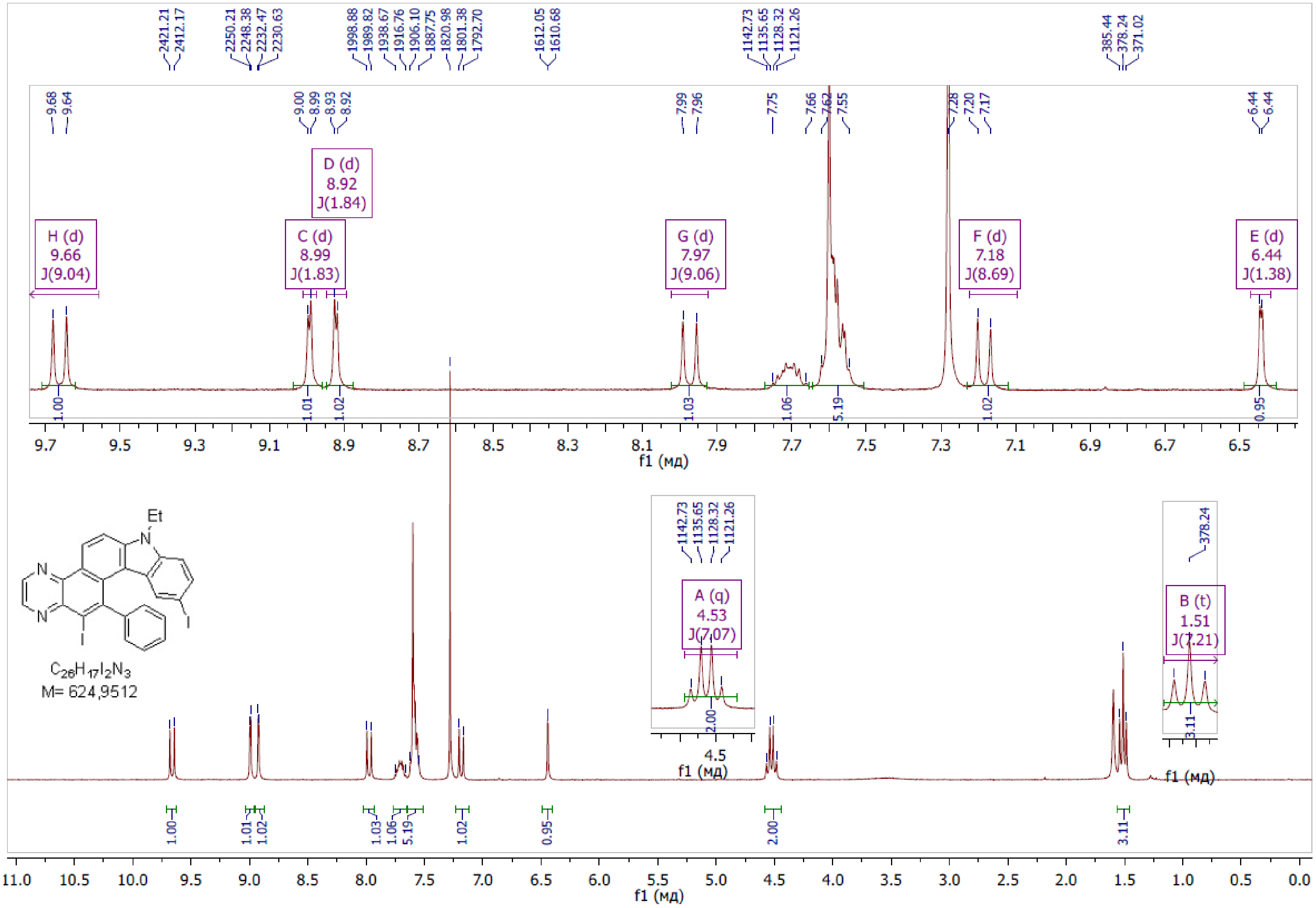

Figure S20. ^{1}H NMR spectrum of **8b** ($CDCl_3$, 250 MHz)

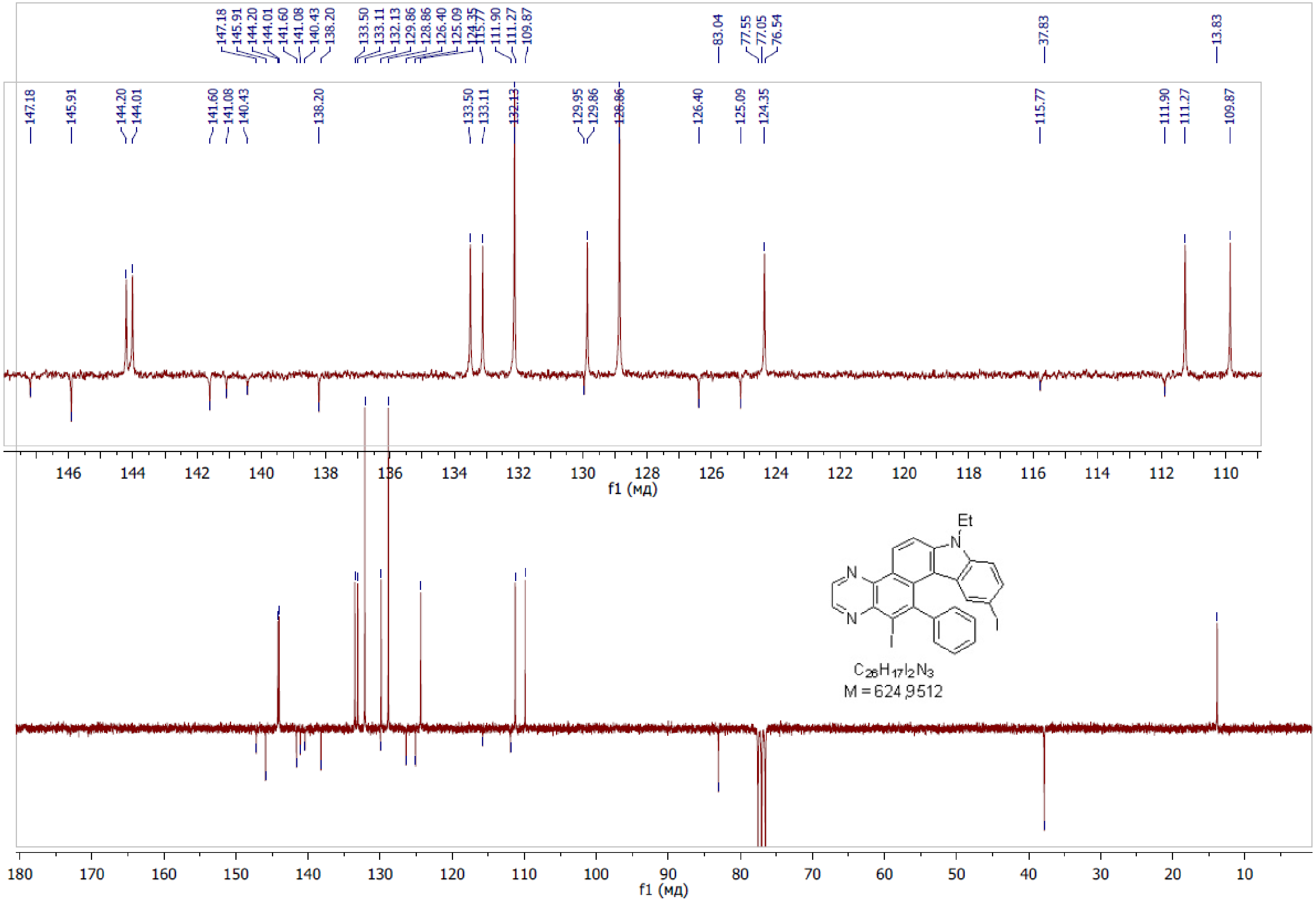

Figure S21. ^{13}C NMR APT spectrum of **8b** ($CDCl_3$, 62.9 MHz)

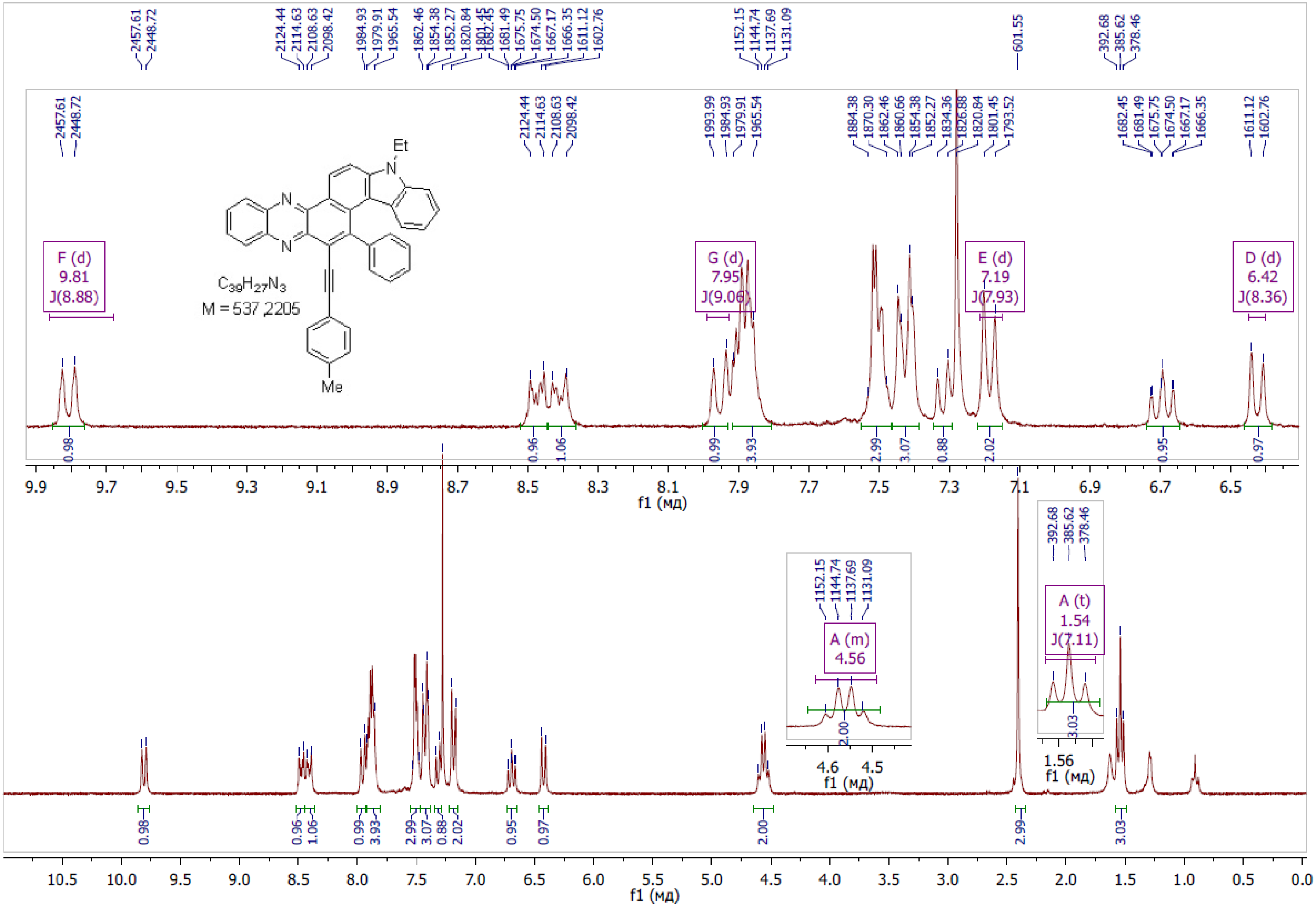

Figure S22. ^{1}H NMR spectrum of **9a** ($CDCl_3$, 250 MHz)

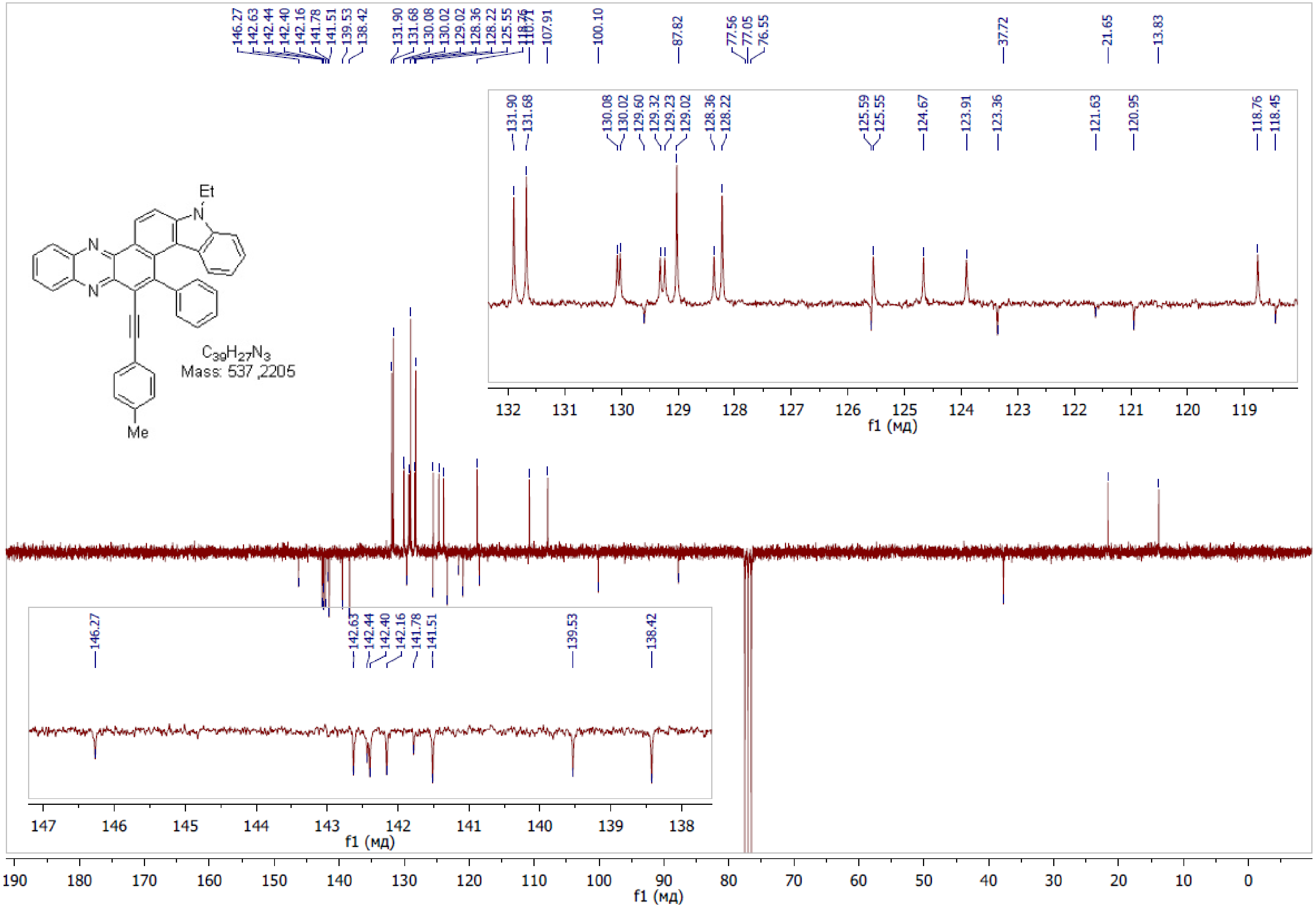

Figure S23. ^{13}C NMR APT spectrum of **9a** ($CDCl_3$, 62.9 MHz)

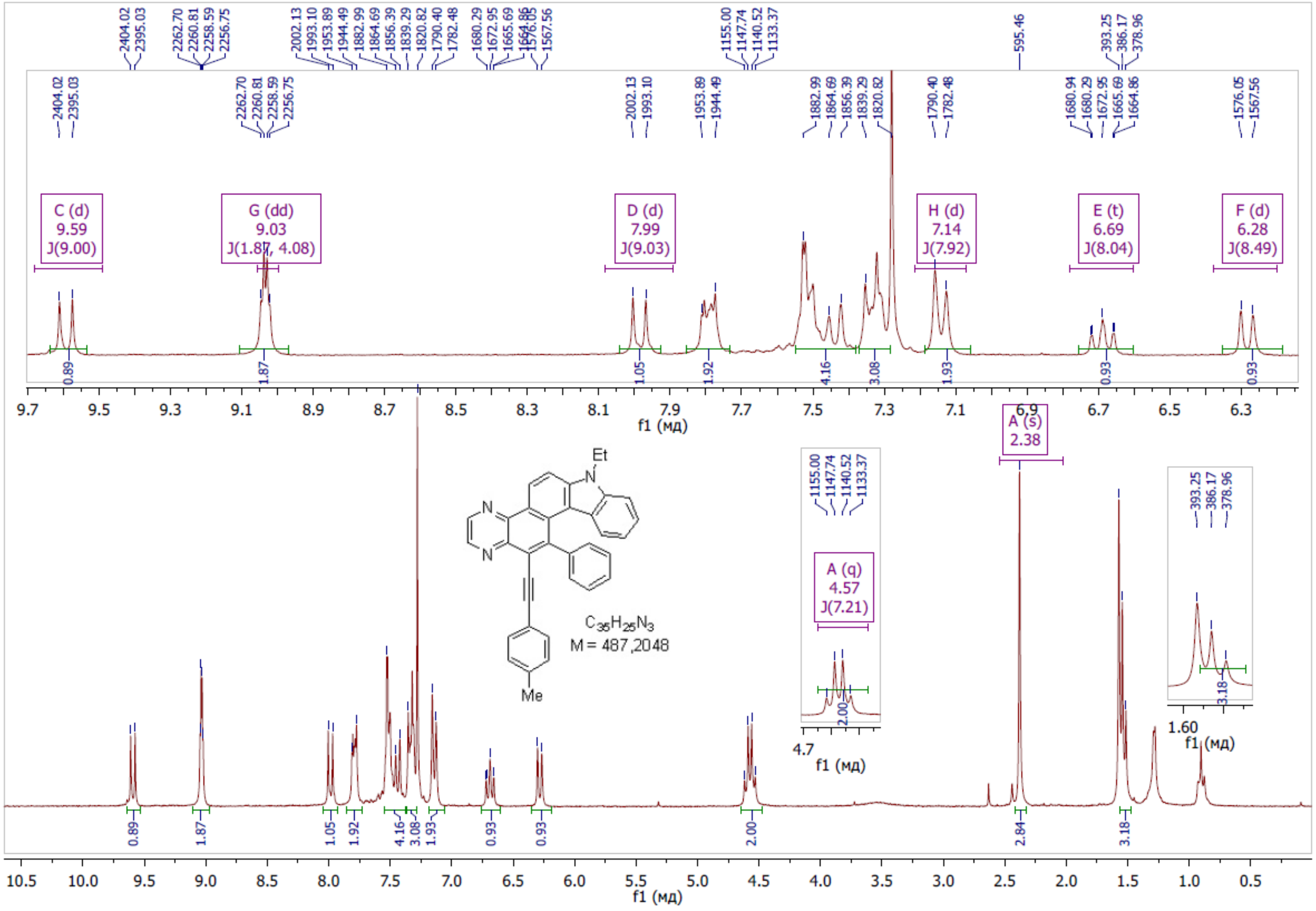

Figure S24. ^{1}H NMR spectrum of **9b** ($CDCl_3$, 250 MHz)

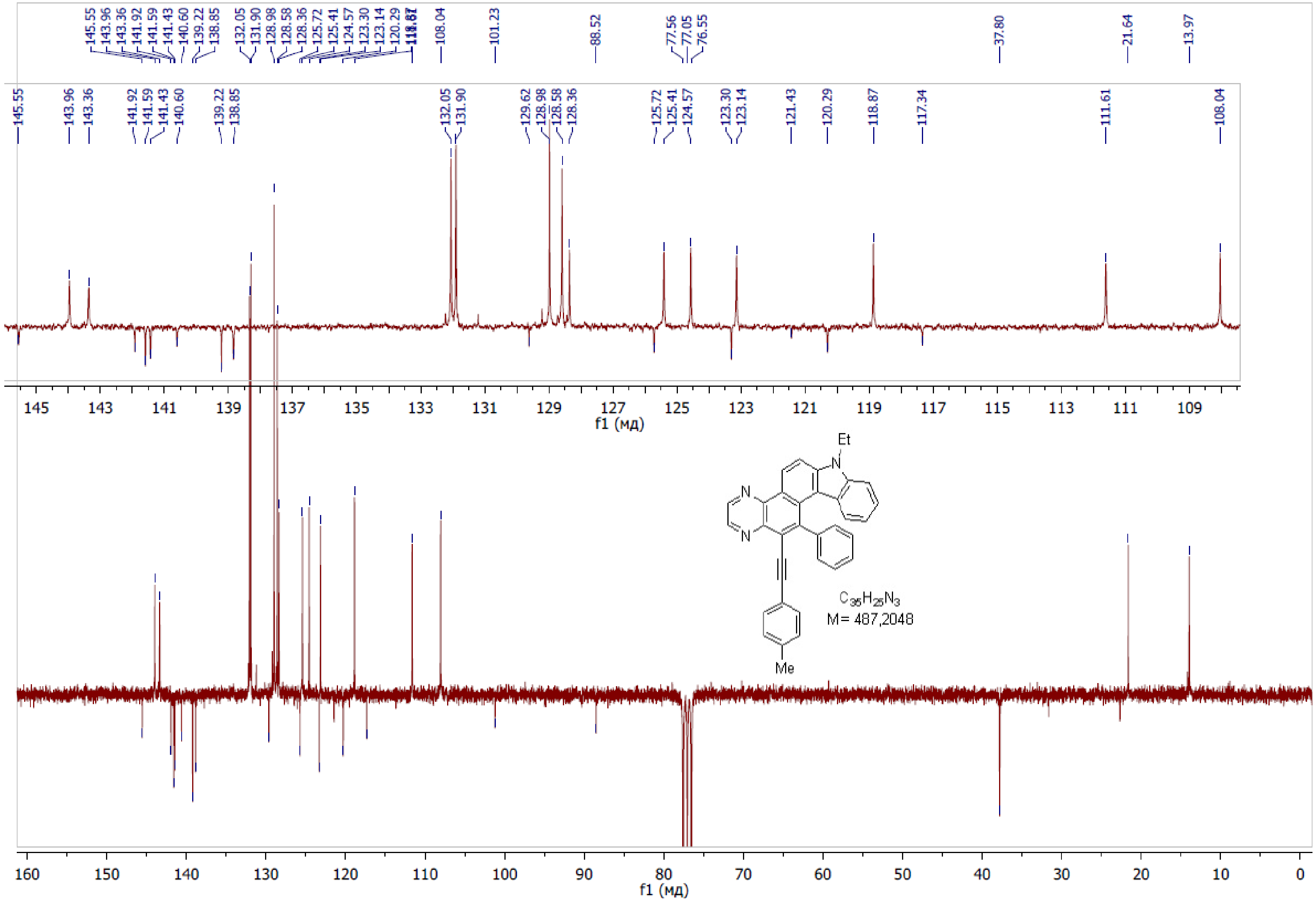

Figure S25. ^{13}C NMR APT spectrum of **9b** ($CDCl_3$, 62.9 MHz)

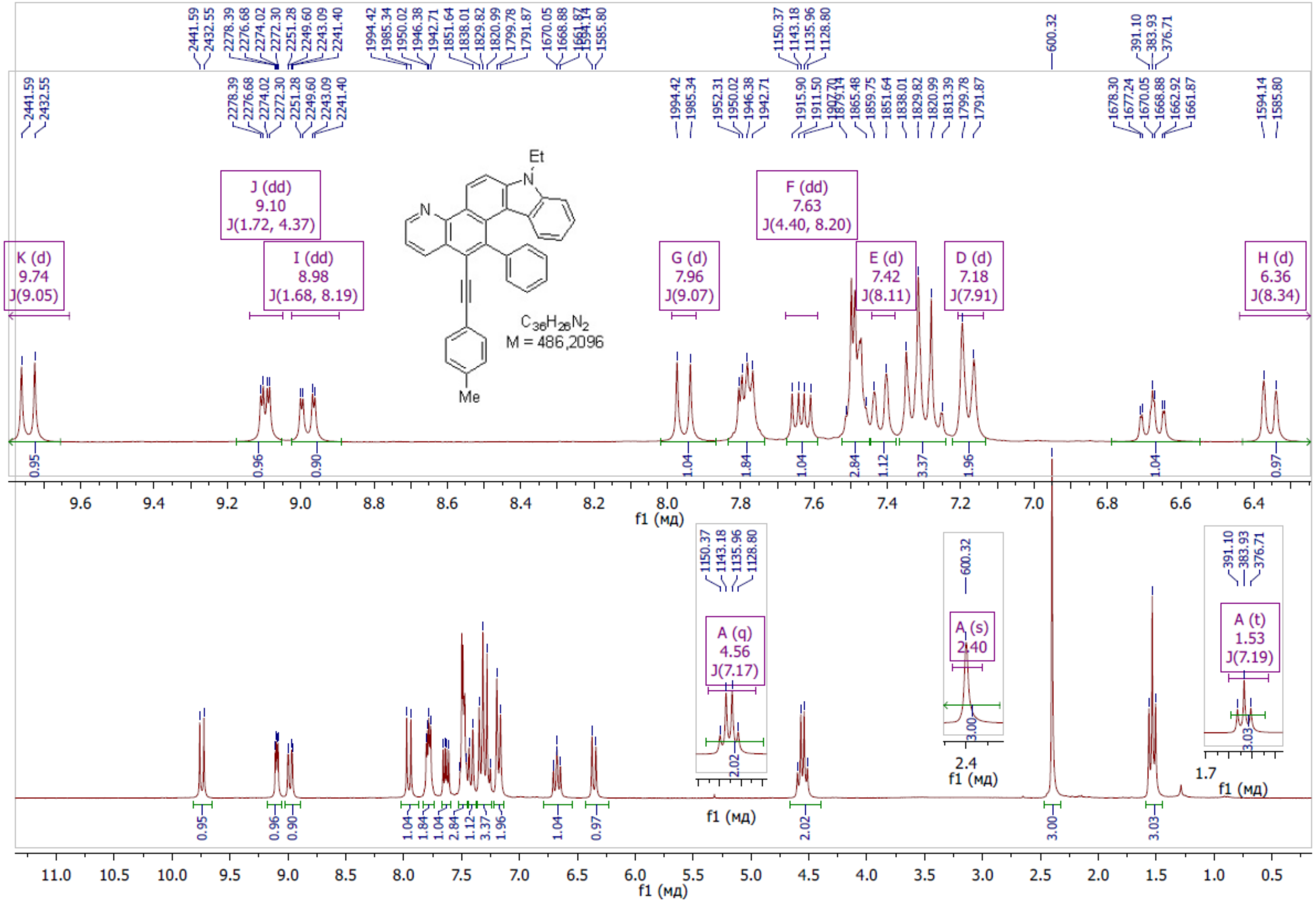

Figure S26. ^{1}H NMR spectrum of **9c** ($CDCl_3$, 250 MHz)

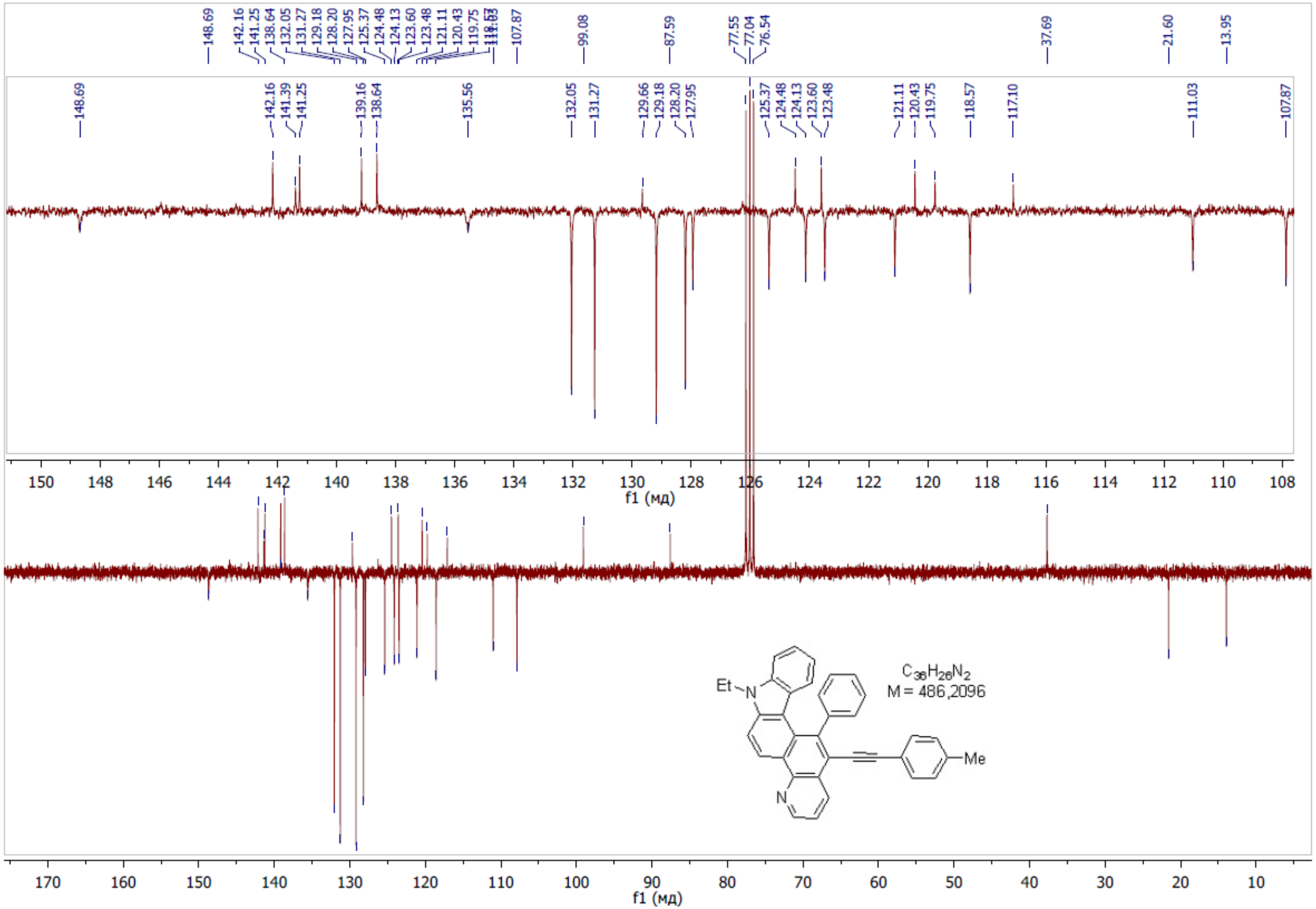

Figure S27. ^{13}C NMR APT spectrum of **9c** ($CDCl_3$, 62.9 MHz)

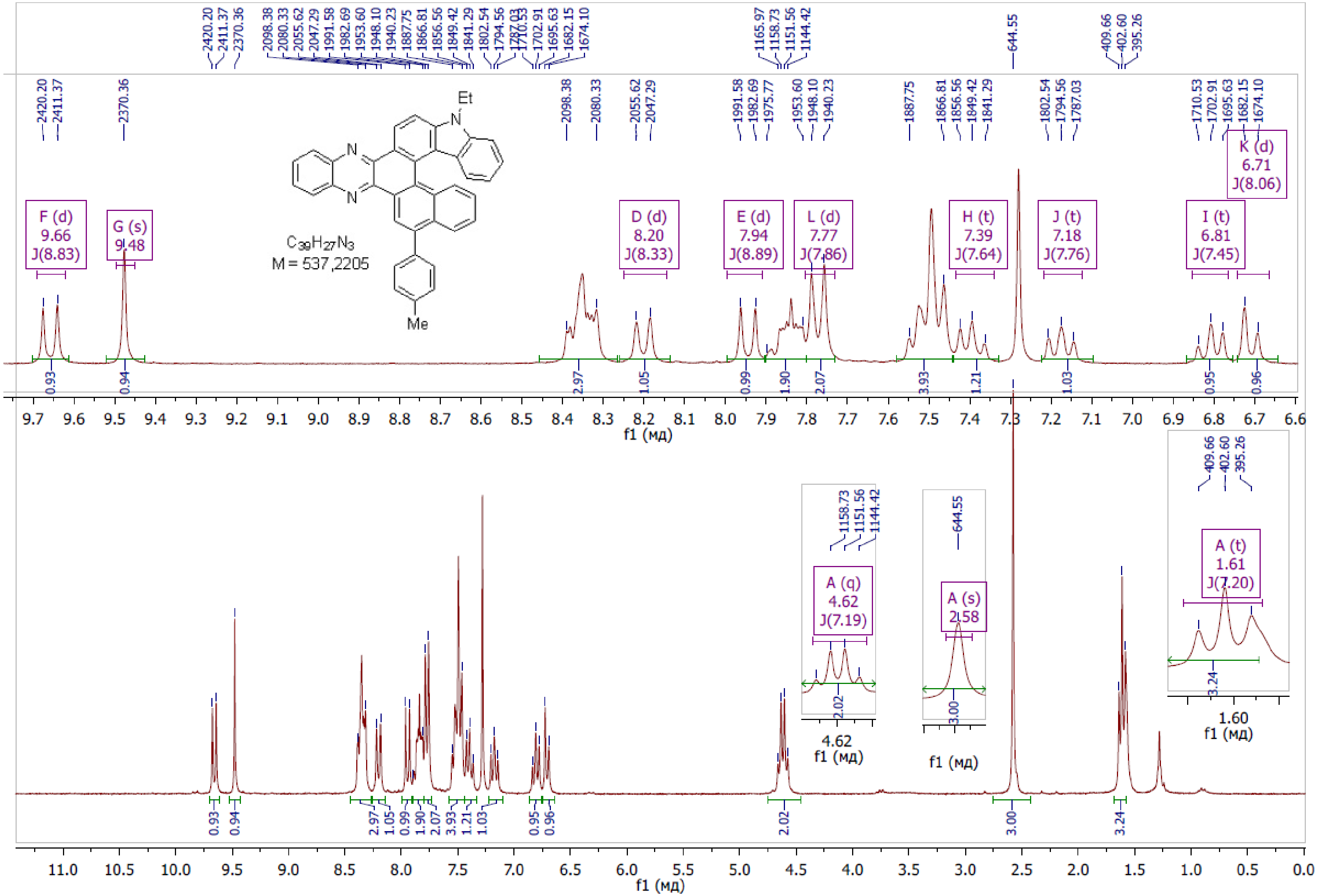

Figure S28. ^{1}H NMR spectrum of **10a** ($CDCl_3$, 250 MHz)

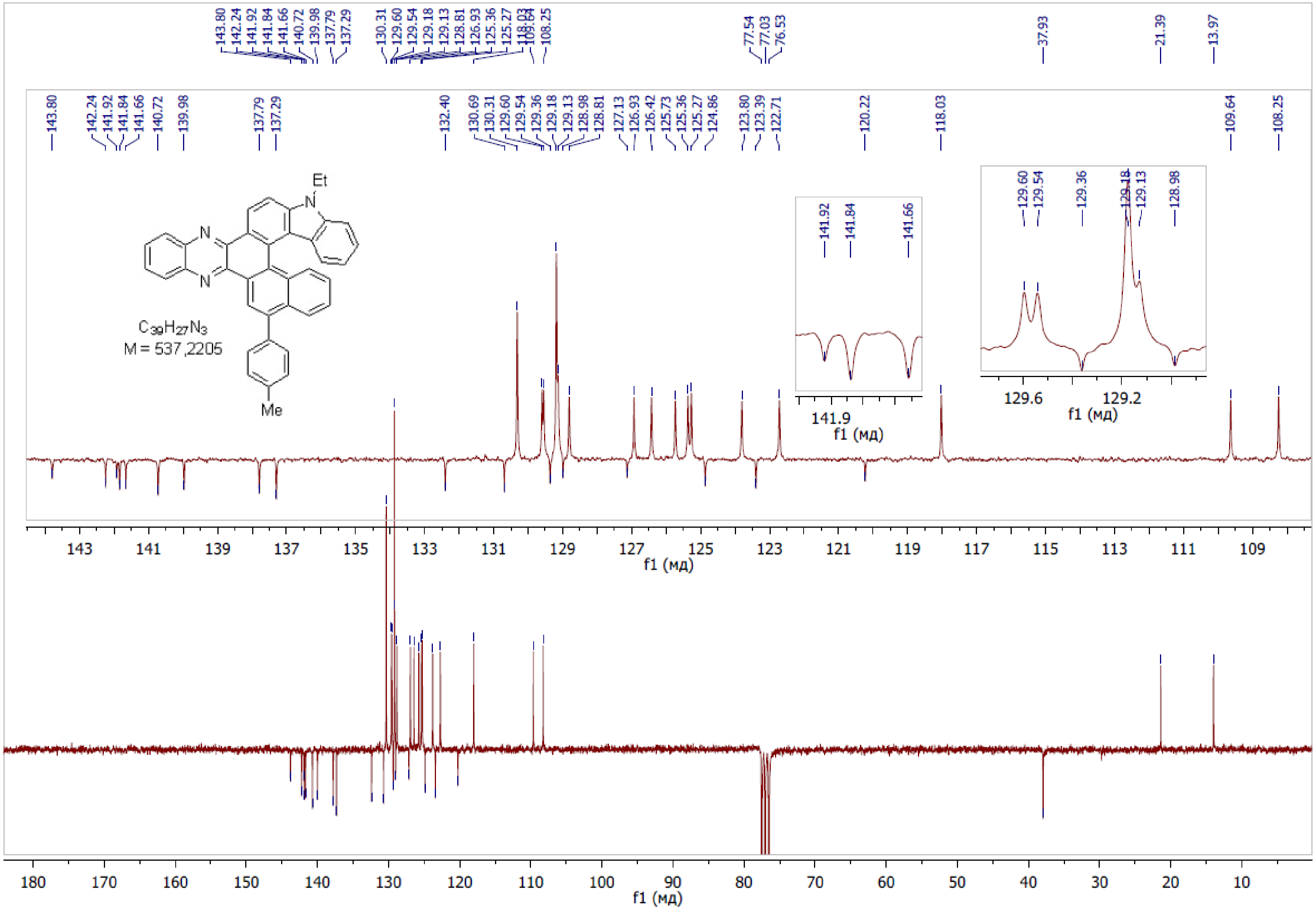

Figure S29. ^{13}C NMR APT spectrum of **10a** ($CDCl_3$, 62.9 MHz)

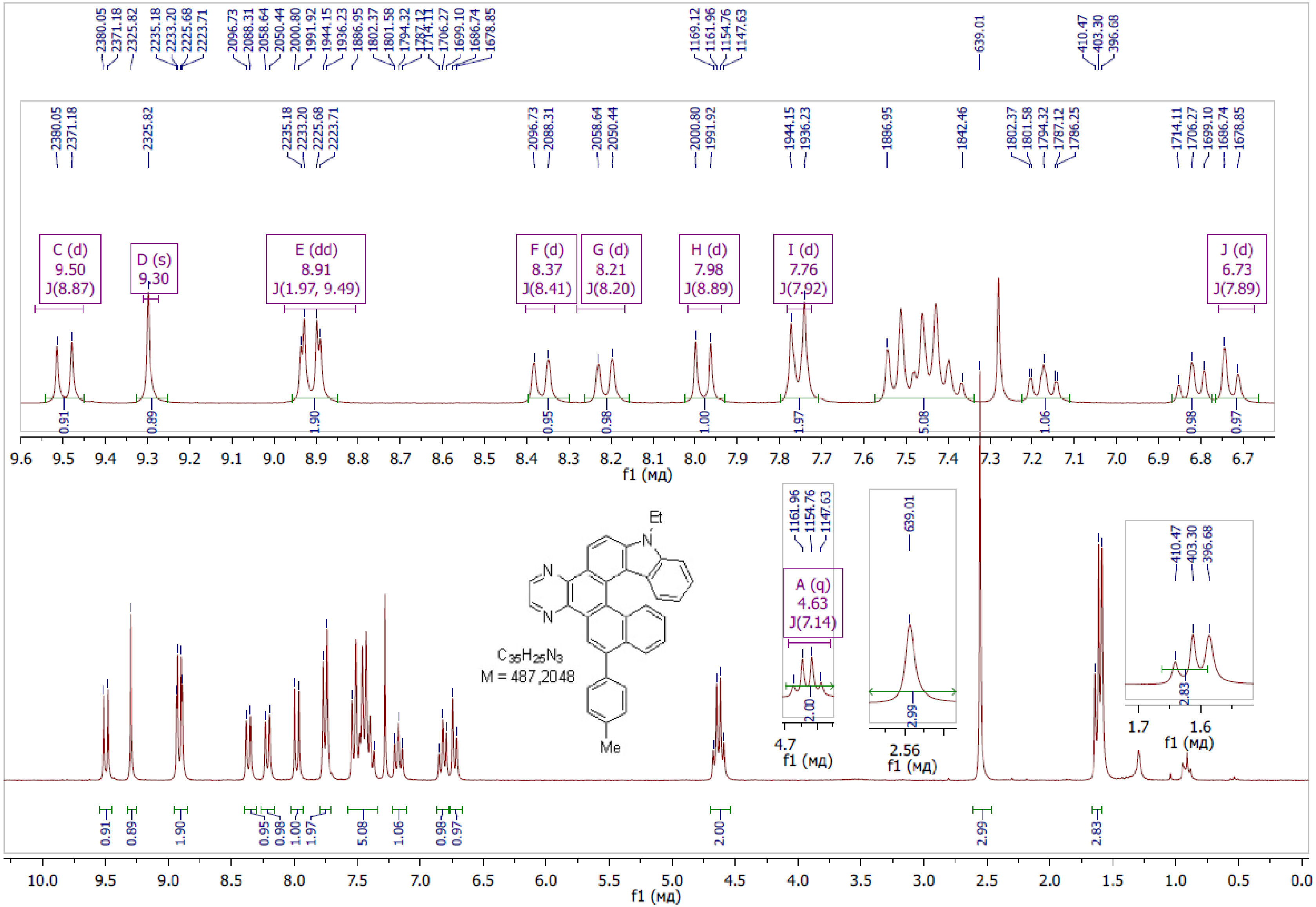

Figure S30. ^{1}H NMR spectrum of **10b** ($CDCl_3$, 250 MHz)

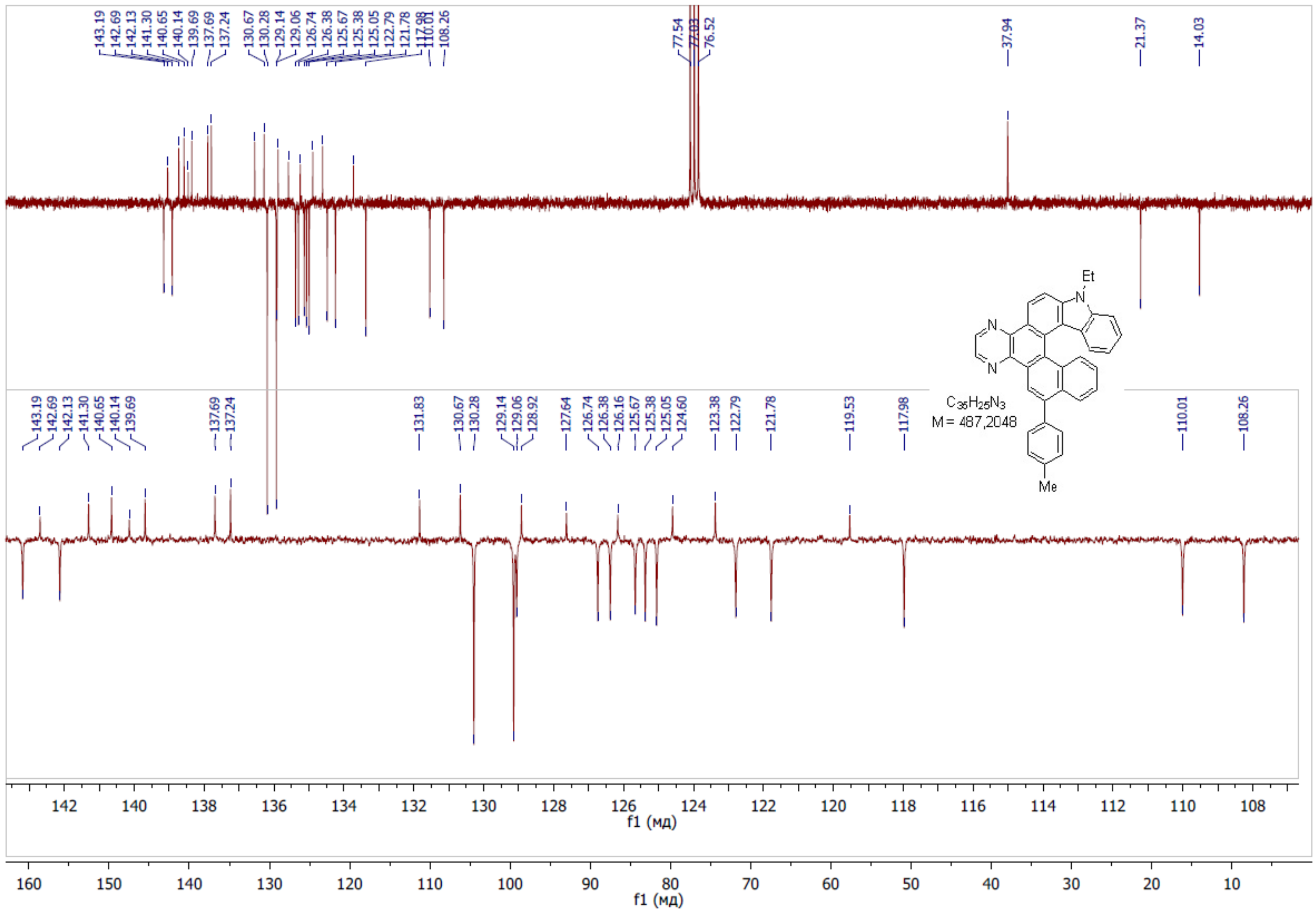

Figure S31. ^{13}C NMR APT spectrum of **10b** ($CDCl_3$, 62.9 MHz)

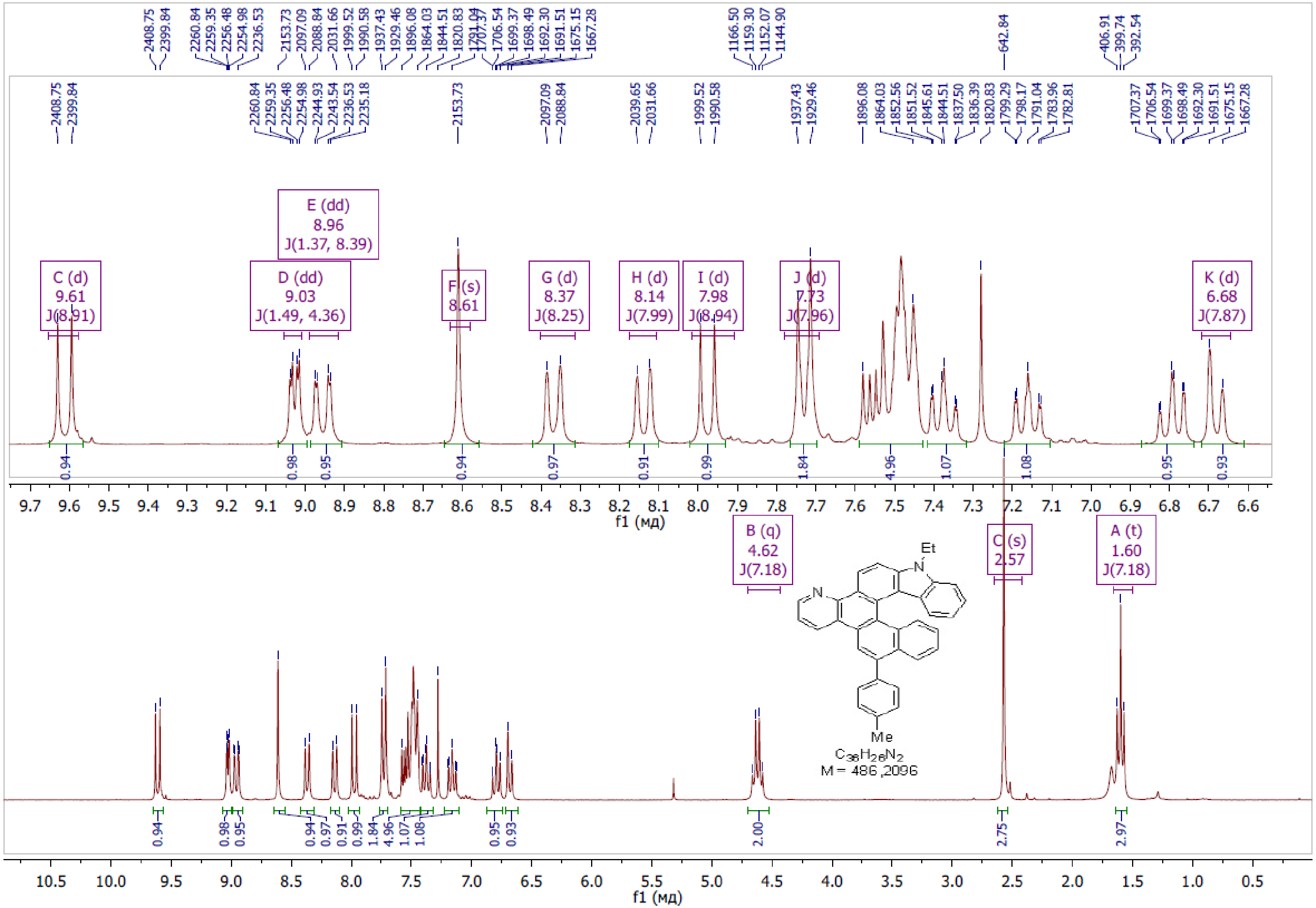

Figure S32. ^{1}H NMR spectrum of **10c** ($CDCl_3$, 250 MHz)

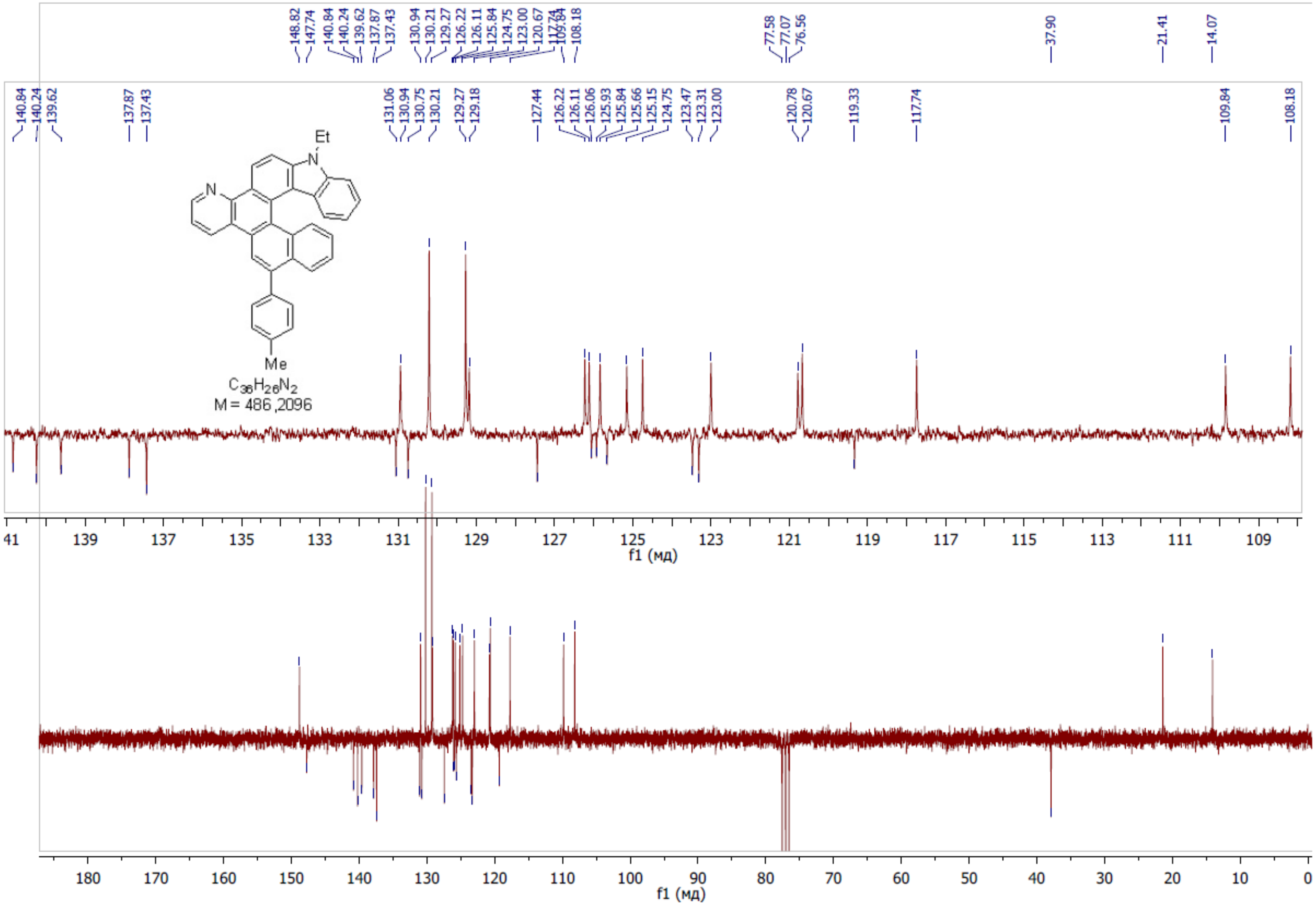

Figure S33. ^{13}C NMR APT spectrum of **10c** ($CDCl_3$, 62.9 MHz)

Crystal structure determination: X-ray measurements were conducted with Bruker APEX II CCD diffractometer and four-circle diffractometer SuperNova, Single source at offset/far, HyPix3000. Atomic coordinates, bond lengths, bond angles and thermal parameters have been deposited at the Cambridge Crystallographic Data Centre (CCDC) and allocated the deposition numbers CCDC 2034941 (**9c**), CCDC 2034943 (**10a**), CCDC 2034944 (**10b**), and CCDC 2034945 (**10c**). These data can be obtained free of charge from the Cambridge Crystallographic Data Centre via www.ccdc.cam.ac.uk/data_request/cif.

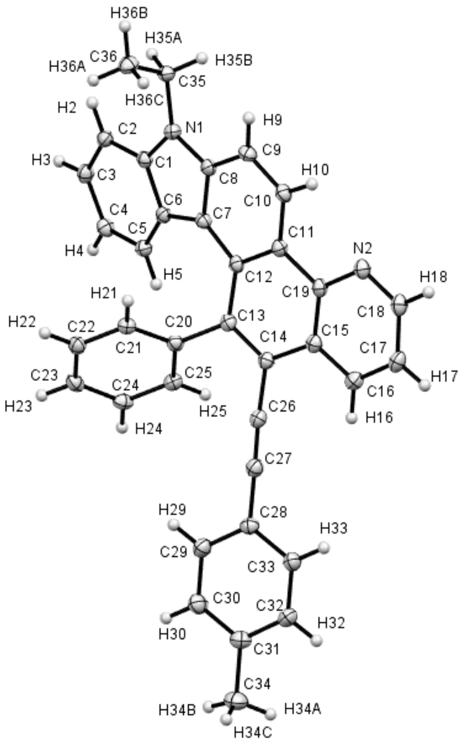

Figure S34. Molecular structure of compound **9c** showing 50% probability amplitude displacement ellipsoids.

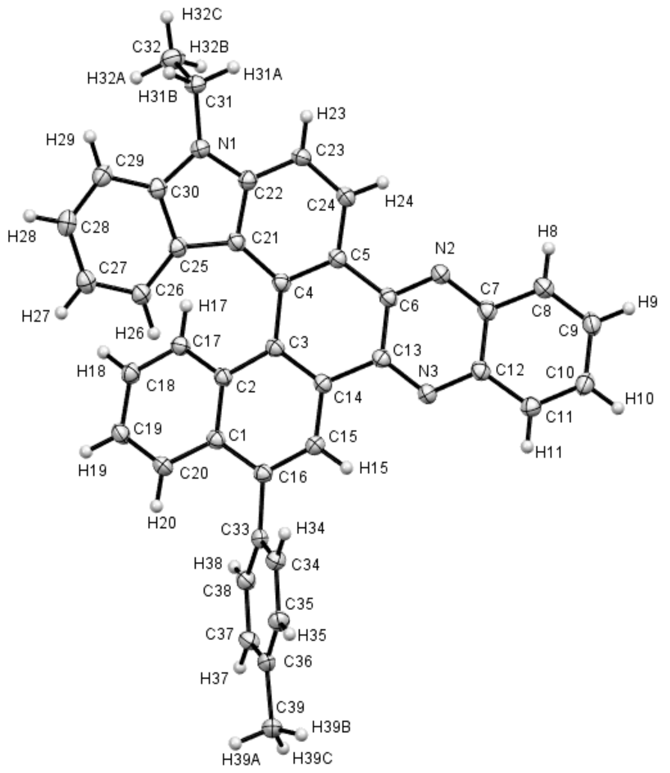

Figure S35. Molecular structure of compound **10a** showing 50% probability amplitude displacement ellipsoids.

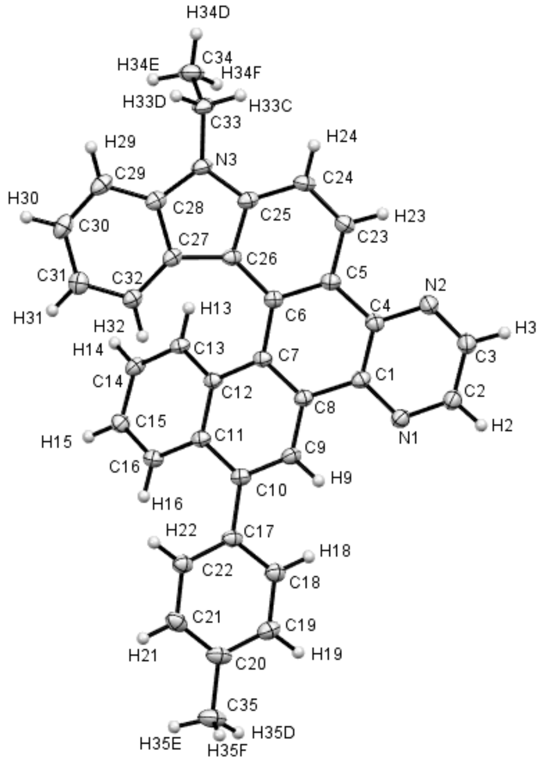

Figure S36. Molecular structure of compound **10b** showing 50% probability amplitude displacement ellipsoids (one of two independent molecules).

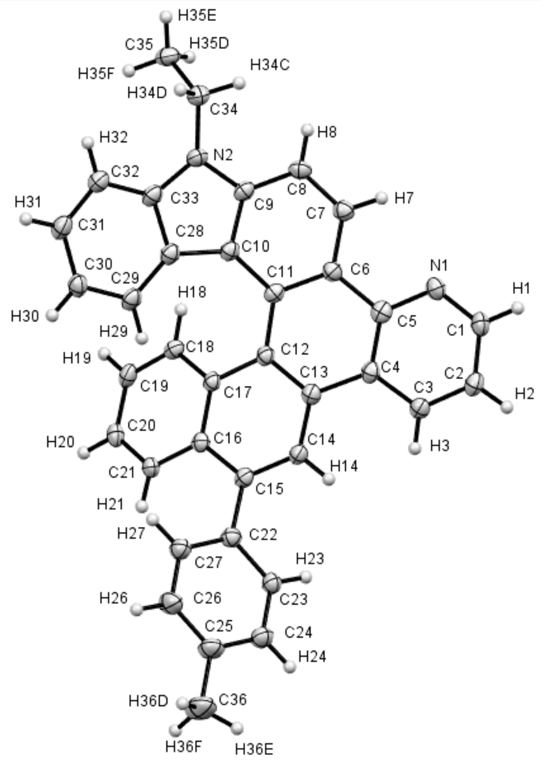

Figure S37. Molecular structure of compound **10c** showing 50% probability amplitude displacement ellipsoids (one of two independent molecules).

Table S1. Crystal data and structure refinement for compounds **9c** and **10a-c**

Compound	**9c**	**10a**	**10b**	**10c**
Empirical formula	C_{36} $H_{26}N_2$	$C_{39}H_{27}N_3$	$2(C_{35}H_{25}N_3)$	$2(C_{36}H_{26}N_2)$
Formula weight	486.59	537.63	975.16	937.17
T [K]	100.01(10)	100.01(16)	100.01(10)	100.00(13)
Crystal system	monoclinic	monoclinic	monoclinic	orthorhombic
Space group	$P2_1/c$	$P2_1/c$	$P2_1/n$	$Pca2_1$
a [Å]	11.61830(10)	9.65530(10)	13.02350(10)	7.97570(10)
b [Å]	8.88780(10)	28.6719(2)	16.31390(10)	14.9619(2)
c [Å]	24.9720(3)	9.54660(10)	23.8982(2)	41.6111(6)
α [°]	90	90	90	90
β [°]	102.2950(10)	92.2720(10)	105.5260(10)	90
γ [°]	90	90	90	90
V [Å^3]	2519.49(5)	2640.76(4)	4892.23(7)	4965.52(12)
Z	4	4	4	4
D_c [g cm^{-3}]	1.283	1.352	1.324	1.302
μ [mm^{-1}]	0.571	0.612	0.602	0.580
No. of refl. collected/ unique	42146/5308 [R_{int} = 0.0342]	35450/5037 [R_{int} = 0.0256]	64970/ 9345 [R_{int} = 0.0285]	32512/8860 [R_{int} = 0.0439]
No. of parameters	346	382	690	690
R indices (all data)	R_1 = 0.0408 wR_2 = 0.1057	R_1 = 0.0357 wR_2 = 0.0883	R_1 = 0.0400 wR_2 = 0.1056	R_1 = 0.0471 wR_2 = 0.1167
R-factor [%]	3.82	3.43	3.66	4.37
CCDC Dep. No.	2034941	2034943	2034944	2034945

The HPLC separations were performed at 25 ºC using Agilent 1200 equipment on the column Kromasil 5-Cellucoat (4.6 mm × 250 mm, particle size 5 μm), photodiode array detector; mobile phase is CH_3CN of HPLC grade; injection of 5 μL of analyte solution in CH_3CN; nominal flow rate is 0.8 mL min^{-1}; UV detection at fixed wavelength 300 nm (for **10a**) and 320 nm (for **10b,c**).

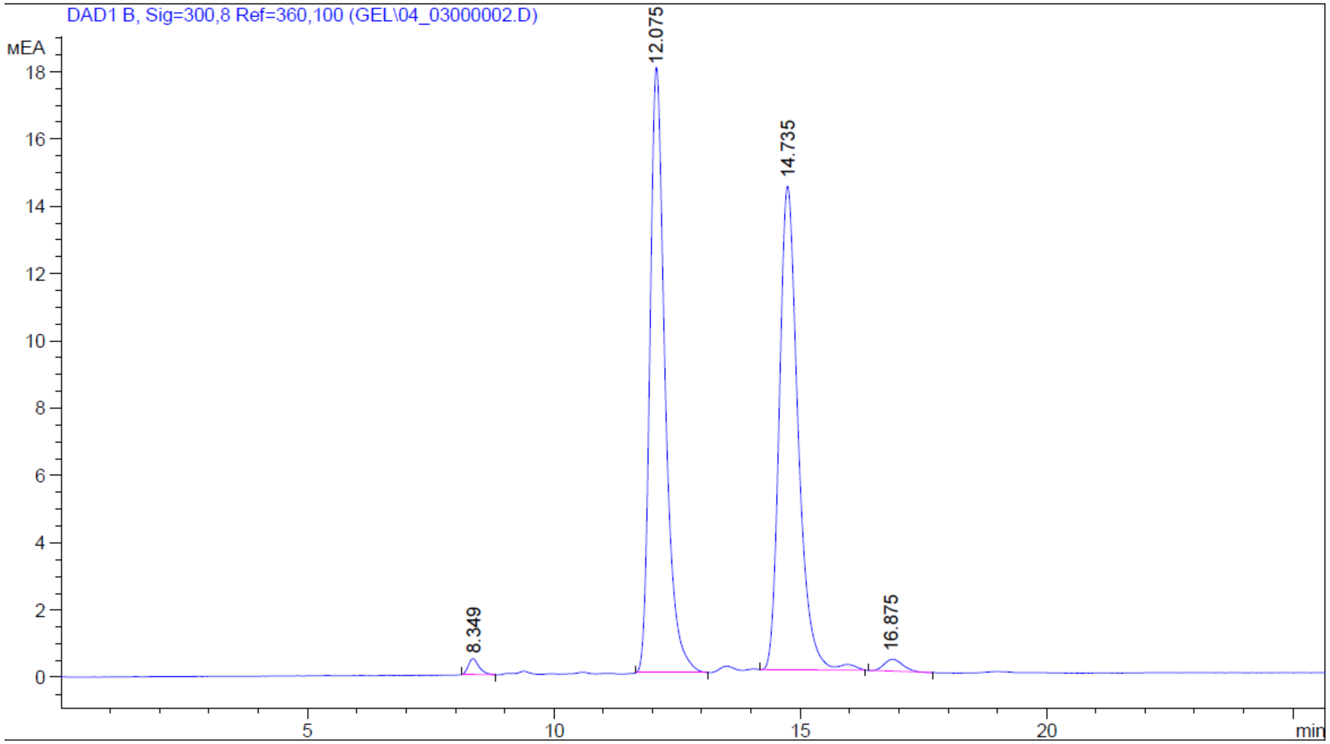

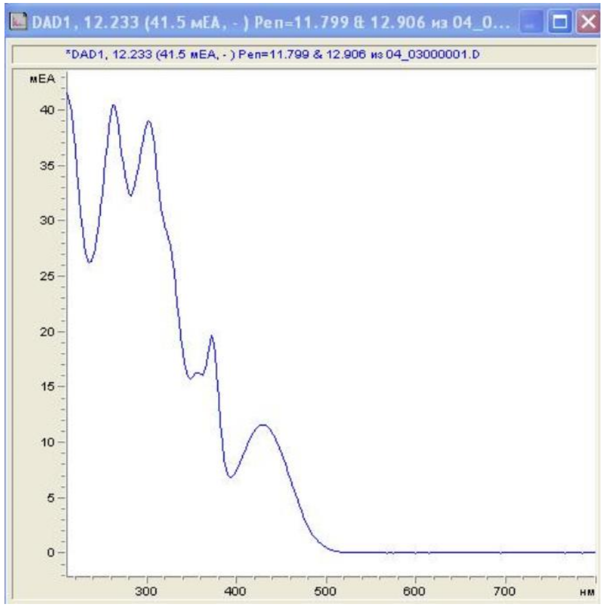

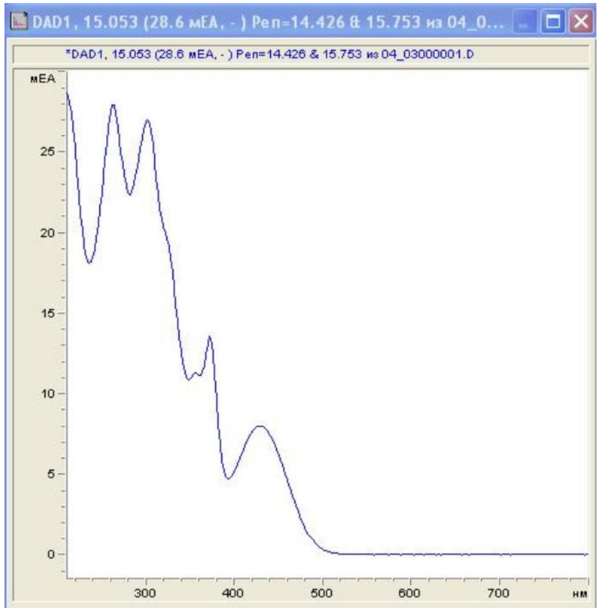

Figure S38. Chromatogram (UV-detection) of (*P*,*M*)-**10a** on Kromasil 5-Cellucoat (mobile phase CH_3CN)

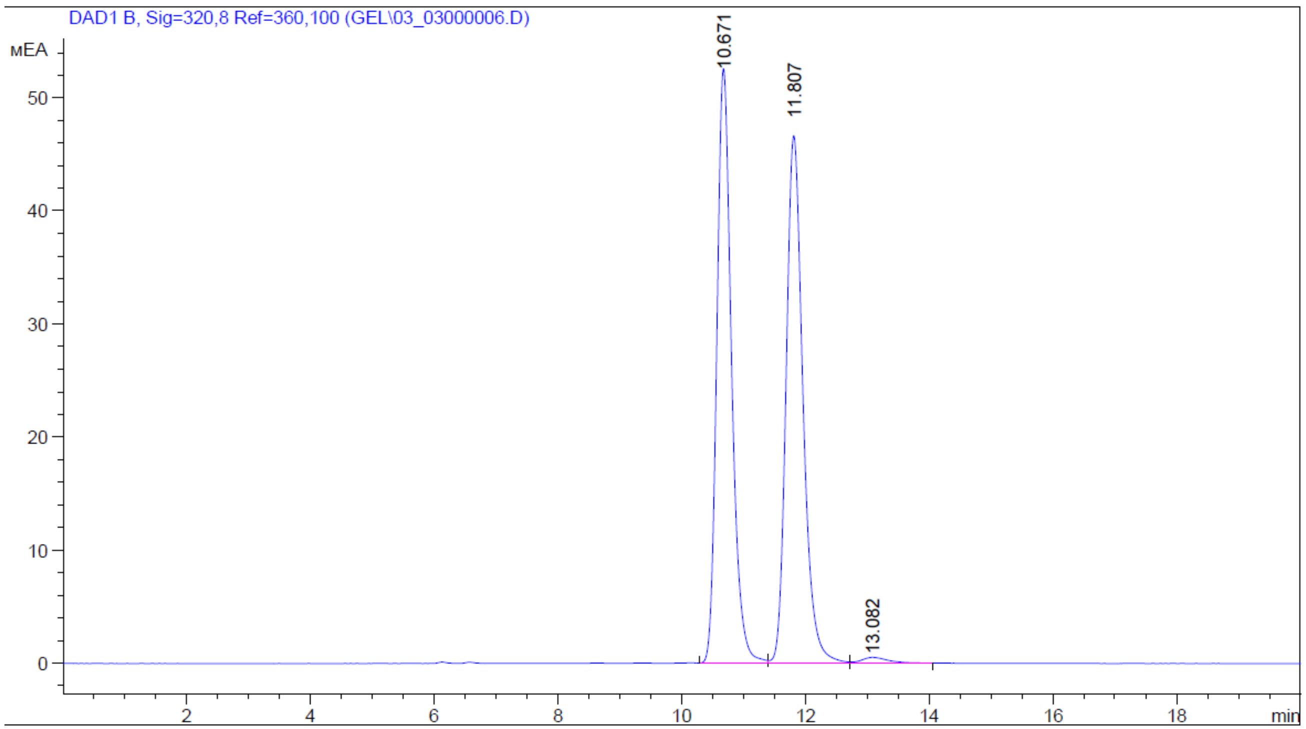

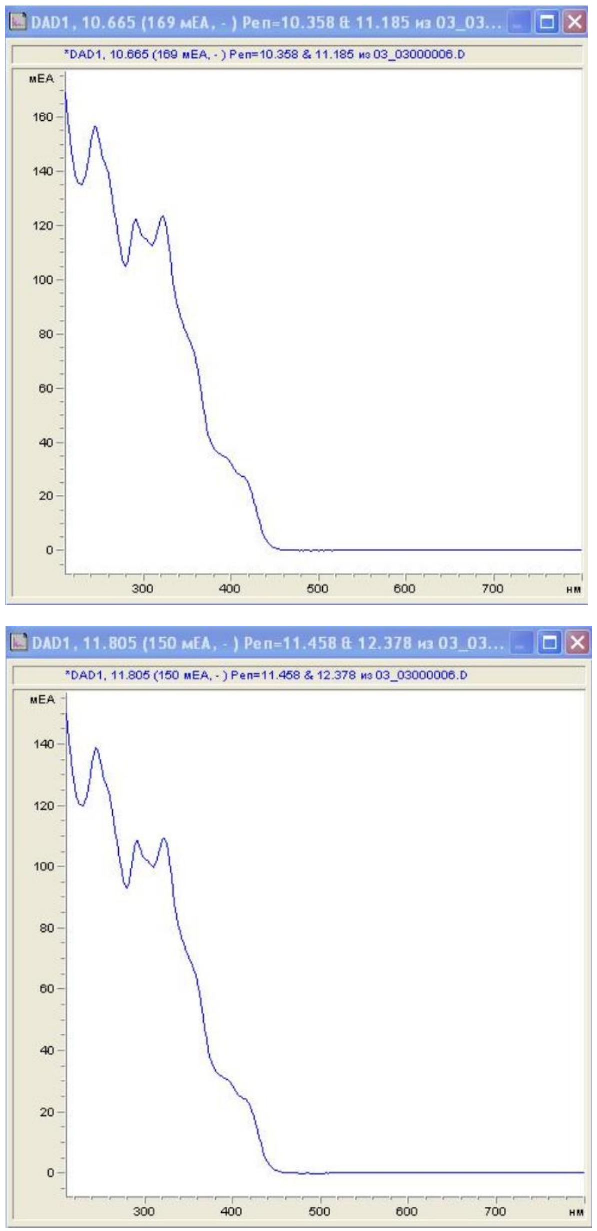

Figure S39. Chromatogram (UV-detection) of (*P*,*M*)-**10b** on Kromasil 5-Cellucoat (mobile phase CH_3CN)

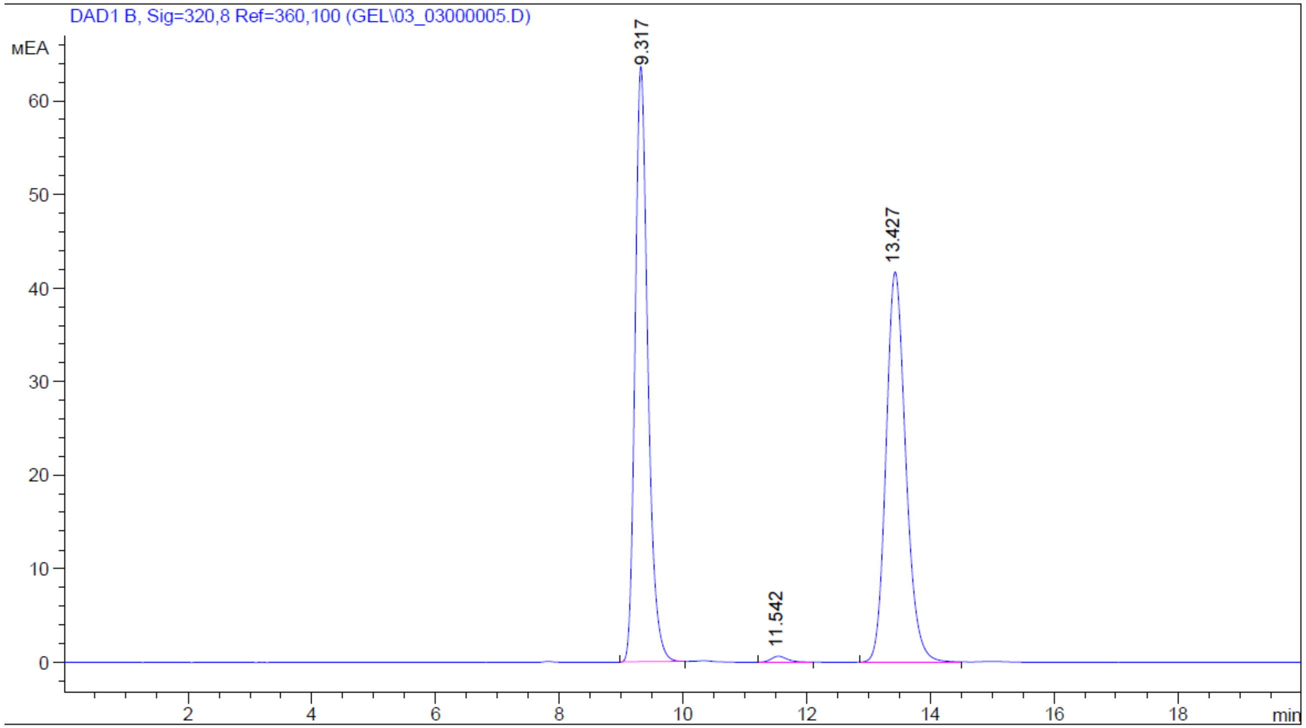

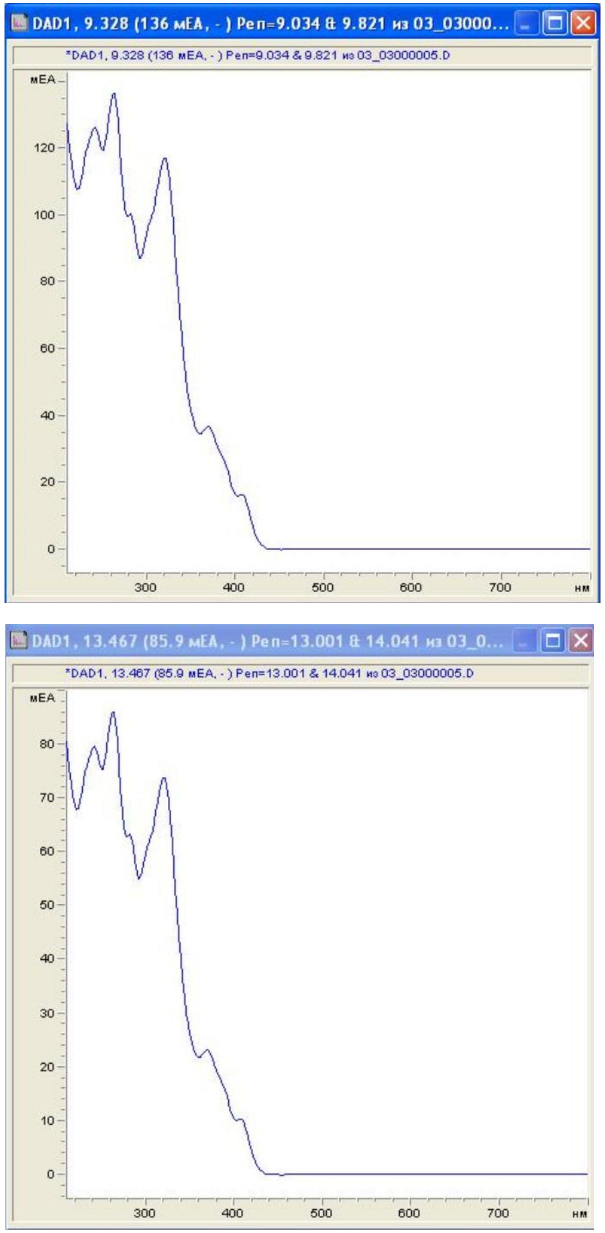

Figure S40. Chromatogram (UV-detection) of (*P*,*M*)-**10c** on Kromasil 5-Cellucoat (mobile phase CH_3CN)

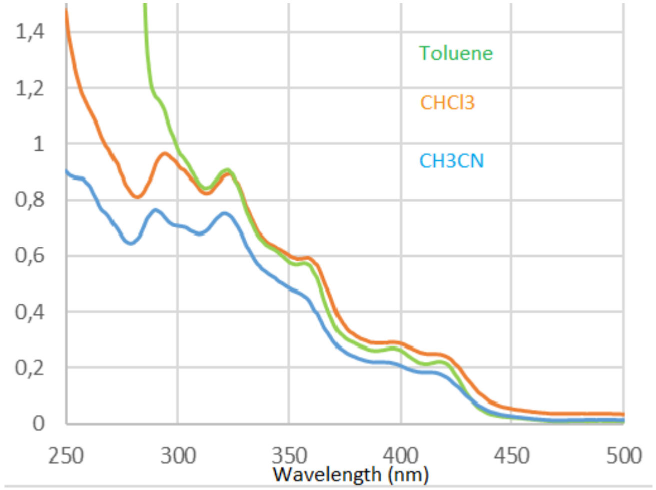

Figure S41: UV–vis spectra of [6]helicene **10b** in different solvents.

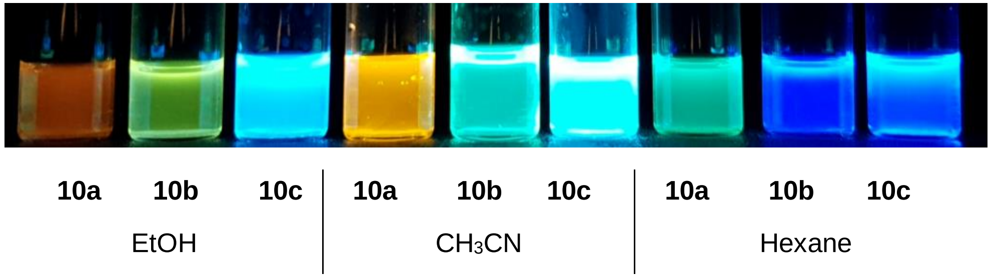

Figure S42: Solutions of [6]helicenes **10** in different solvents under UV irradiation (365 nm).

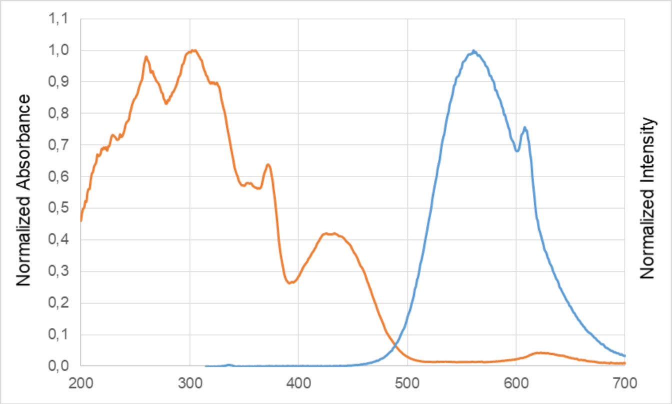

Figure S43: Normalized absorption and fluorescence spectra of **10a** in acetonitrile.

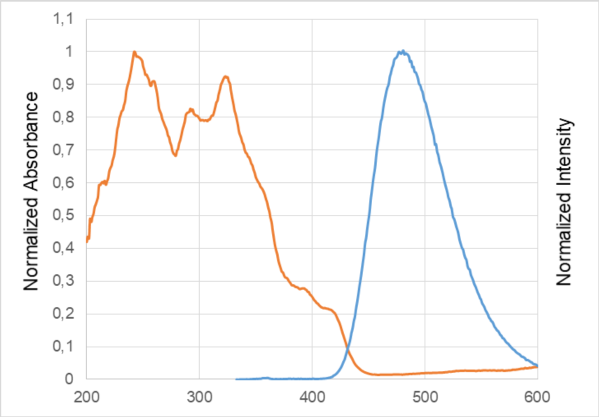

Figure S44: Normalized absorption and fluorescence spectra of **10b** in acetonitrile.

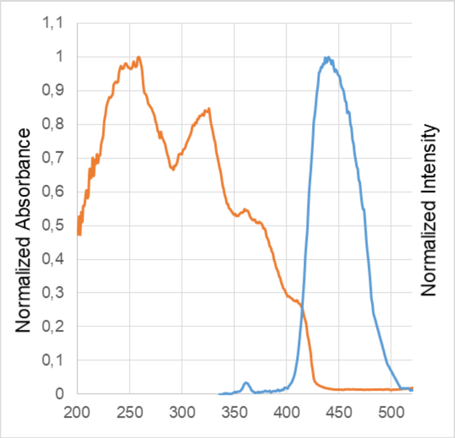

Figure S45: Normalized absorption and fluorescence spectra of **10c** in acetonitrile.

References

1. Gulevskaya, A. V.; Shvydkova, E. A.; Tonkoglazova, D. I. *Eur. J. Org. Chem.* **2018**, 5030–5043. doi:10.1002/ejoc.201800613
